# A comprehensive excitatory input map of the striatum reveals novel functional organization

**DOI:** 10.7554/eLife.19103

**Published:** 2016-11-28

**Authors:** Barbara J Hunnicutt, Bart C Jongbloets, William T Birdsong, Katrina J Gertz, Haining Zhong, Tianyi Mao

**Affiliations:** Vollum Institute, Oregon Health and Science University, Portland, United States; Washington University in St Louis, United States

**Keywords:** striatal circuitry, thalamostriatal projections, mesoscopic connectome, corticostriatal projections, striatal subdivisions, Mouse

## Abstract

The striatum integrates excitatory inputs from the cortex and the thalamus to control diverse functions. Although the striatum is thought to consist of sensorimotor, associative and limbic domains, their precise demarcations and whether additional functional subdivisions exist remain unclear. How striatal inputs are differentially segregated into each domain is also poorly understood. This study presents a comprehensive map of the excitatory inputs to the mouse striatum. The input patterns reveal boundaries between the known striatal domains. The most posterior striatum likely represents the 4th functional subdivision, and the dorsomedial striatum integrates highly heterogeneous, multimodal inputs. The complete thalamo-cortico-striatal loop is also presented, which reveals that the thalamic subregions innervated by the basal ganglia preferentially interconnect with motor-related cortical areas. Optogenetic experiments show the subregion-specific heterogeneity in the synaptic properties of striatal inputs from both the cortex and the thalamus. This projectome will guide functional studies investigating diverse striatal functions.

**DOI:** http://dx.doi.org/10.7554/eLife.19103.001

## eLife digest

To fully understand how the brain works, we need to understand how different brain structures are organized and how information flows between these structures. For example, the cortex and thalamus communicate with another structure known as the basal ganglia, which is essential for controlling voluntary movement, emotions and reward behaviour in humans and other mammals. Information from the cortex and the thalamus enters the basal ganglia at an area called the striatum. This area is further divided into smaller functional regions known as domains that sort sensorimotor, emotion and executive information into the basal ganglia to control different types of behaviour. Three such domains have been identified in the striatum of mice. However, the boundaries between these domains are vague and it is not clear whether any other domains exist or if the domains can actually be divided into even smaller areas with more precise roles.

Information entering the striatum from other parts of the brain can either stimulate activity in the striatum (known as an “excitatory input”) or alter existing excitatory inputs. Now, Hunnicutt et al. present the first comprehensive map of excitatory inputs into the striatum of mice. The experiments show that while many of the excitatory inputs flowing into the striatum from the cortex and thalamus are sorted into the three known domains, a unique combination of the excitatory inputs are sorted into a new domain instead. One of the original three domains of the striatum is known to relay information related to associative learning, for example, linking an emotion to a person or place. Hunnicutt et al. show that this domain has a more complex architecture than the other domains, being made up of many distinct areas. This complexity may help it to process the various types of information required to make such associations.

The findings of Hunnicutt et al. provide a framework for understanding how the striatum works in healthy and diseased brains. Since faulty information processing in the striatum is a direct cause of Parkinson’s disease, Huntington’s disease and other neurological disorders in humans, this framework may aid the development of new treatments for these disorders.

**DOI:** http://dx.doi.org/10.7554/eLife.19103.002

## Introduction

The basal ganglia play an essential role in movement control and action selection (Balleine et al., 2009; Graybiel et al., 1994; Jin and Costa, 2010; Packard and Knowlton, 2002; Wilson, 2004; Yin and Knowlton, 2006). Their primary input station — the striatum — sorts contextual, motor, and reward information from its two major excitatory input sources, the cortex and the thalamus, into specific downstream pathways (Berendse and Groenewegen, 1990; Gerfen, 1992; Gerfen and Wilson, 1996, Gerfen and Bolam, 2010; Smith et al., 2011). The thalamus, which extensively interconnects with the cortex (Sherman and Guillery, 2009), is also a primary output target of the basal ganglia (Haber and Calzavara, 2009; Parent and Hazrati, 1995). Knowledge of the precise circuits and organizational principles between the cortex, thalamus, and striatum is essential for the mechanistic dissection of how these structures orchestrate function.

Neuronal circuits within large brain structures, such as the cortex and the thalamus, are organized around functional subregions. For example, the cortex contains many distinct functional areas, including the sensory subregions, which are defined by specific sensory inputs, and the motor subregions, which are defined by intracortical microstimulation (Li and Waters, 1991). The thalamic subregions have traditionally been defined by cytoarchitectural boundaries to delineate ~40 nuclei (Jones, 2007). In contrast, the spatial organization of the striatum is poorly defined, particularly in mice. The striatum is thought to contain three functional domains: the sensorimotor, associative, and limbic domains, which approximately correspond to the dorsolateral, dorsomedial, and ventral striatum, respectively (Balleine et al., 2009; Belin et al., 2009; Gruber and McDonald, 2012; Thorn et al., 2010; Yin and Knowlton, 2006; Yin et al., 2005). However, the precise demarcations between these striatal domains remain unclear, and it is not known whether each striatal domain contains finer levels of organization. Notably, although the striatum extends a significant length along the anterior-posterior (A-P) axis (~4 mm in mice), the existence of domain heterogeneity along this axis remains elusive.

Although the striatum lacks accepted domain demarcations, it is known to have stereotypic excitatory input patterns (Averbeck et al., 2014; Berendse et al., 1992; Kincaid and Wilson, 1996; Selemon and Goldman-Rakic, 1985). For example, in primates, the motor cortex has been shown to project to the rostral putamen, which corresponds to the rostral dorsolateral striatum, whereas the premotor cortex projects to the rostral caudate, which corresponds to the rostral dorsomedial striatum (Künzle, 1975). Investigation of the topographic arrangement of corticostriatal inputs from selected cortical subregions, or to isolated parts of the striatum have also been initiated in mice (Guo et al., 2015; Pan et al., 2011; Wall et al., 2013). However, the precise projection patterns from most cortical subregions to the entire striatum remain to be systematically characterized. Furthermore, the organization of thalamostriatal inputs, which provide ~1/3 of all glutamatergic synapses in the striatum (Huerta-Ocampo et al., 2014), has been less studied. In primates, the centromedian/parafascicular (CM/Pf) complex of the thalamus has been the main focus in studies of thalamostriatal function (François et al., 1991; Smith et al., 2011), yet less is known about the thalamostriatal projections from other thalamic subregions.

The lack of systematic anatomical maps of corticostriatal and thalamostriatal inputs has stymied efforts to dissect the cortico-thalamo-striatal triangular circuits. For example, recent functional studies suggest that corticostriatal and thalamostriatal axons differ in their release probability and plasticity properties (Ding et al., 2008; Smeal et al., 2007), but the precise differences have been controversial (Ding et al., 2008; Smeal et al., 2007). This controversy raises the possibility of heterogeneity within axons originating from different cortical or thalamic subregions in their synaptic properties (Kreitzer and Malenka, 2008). A comprehensive excitatory input wiring diagram will provide a road map to enable systematic examination of the differential function of individual inputs. In addition, since the excitatory input patterns are thought to be stereotypic in the striatum, we reasoned that the striatal subregions and their boundaries may be revealed by systematic analysis of these input patterns from individual cortical and thalamic subregions.

Here, we provide a quantitative and comprehensive description of cortical and thalamic inputs to the mouse striatum. This is achieved by integrating an in-house comprehensive thalamic anterograde projection dataset (Hunnicutt et al., 2014) and a selected cortical projection dataset from the Allen Institute for Brain Sciences (AIBS) (Oh et al., 2014). Analyses of this striatal excitatory input wiring diagram revealed clear boundaries separating the three traditional striatal domains and uncovered a fourth subdivision in the posterior striatum. The dorsomedial striatum exhibited the highest degree of cortical input heterogeneity, suggesting that this subdivision serves as an information hub. In addition, thalamic subregions receiving basal ganglia outputs are preferentially interconnected with motor-related cortical subregions. With all the pathways tested, the anatomically described corticostriatal and thalamostriatal projections were confirmed to be functional using optogenetic approaches. Importantly, striatal inputs originating from different cortical or thalamic subregions form synapses in the striatum with distinct plasticity properties. Our findings lay the foundation for understanding the function of the striatum and its interactions with the cortex and the thalamus.

## Results

### Integration of cortical and thalamic injection datasets

**Figure 1.**
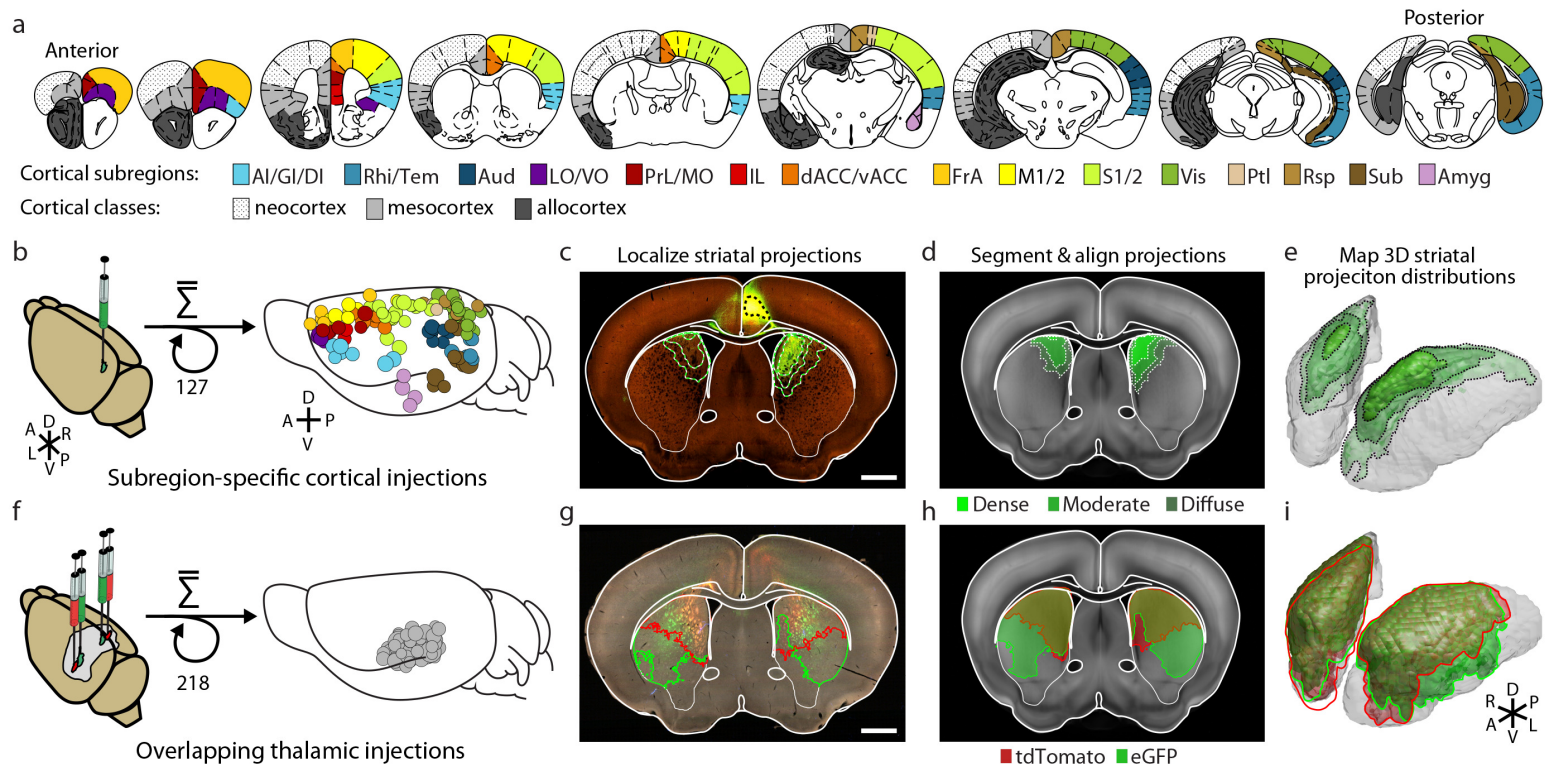
Integration of two large-scale anatomical datasets to investigate whole-brain striatal input convergence. (**a**) Coronal atlas sections showing the 15 cortical subregions targeted by cortical injections (right of each section) and the cortical classes they encompass (left of each section, modified from the Paxinos Mouse Brain Atlas (PMBA) (Paxinos and Franklin, 2001). (**b–e**) Overview of corticostriatal connectivity data generation. (**b**) Unilateral injection of virus expressing eGFP (green) in the mouse cortex (left). A total of 127 injections were used to sample the entire cortex (15 cortical subregions analyzed, right) from AIBS. (**c**) Representative coronal section showing a cortical injection (dashed black line) and segmented striatal projections with three projection density thresholds (green lines). (**d**) Corticostriatal projections localized within the AIBS averaged template brain (gray). (**e**) An example 3D view of corticostriatal projections. (**f–i**) Overview of thalamostriatal connectivity data generation. (**f**) Bilateral injections of virus expressing tdTomato (red) and eGFP (green) in the mouse thalamus (left). A total of 218 injections were localized and aligned within an average model thalamus (Hunnicutt et al., 2014) (right). (**g**) Representative coronal section showing thalamostriatal projection localization in high-resolution images (red and green outlines). (**h**) Each striatum is aligned to the AIBS average template brain. (**i**) Example 3D view of thalamostriatal projections. **DOI:** http://dx.doi.org/10.7554/eLife.19103.003

In order to obtain a comprehensive excitatory map of the striatum, two viral-based anterograde fluorescent-tracing datasets (Hunnicutt et al., 2014; Oh et al., 2014) were analyzed and combined (Figure 1). The cortex has large, well-defined subregions. A relatively sparse set of viral injections confined to individual cortical subregions can therefore be used to localize cortical projections (Figure 1a–b). We visually inspected all (1029 at the time) injections from AIBS Mouse Brain Connectivity Atlas (AMBA, http://connectivity.brain-map.org) (Oh et al., 2014) and identified 127 injections that were well confined to individual cortical subregion boundaries (Figure 1a–c, Supplementary file 1, and Materials and methods). Other injections from original dataset were not included primarily because many of them span two or more cortical subregions (Oh et al., 2014).

**Figure 1—figure supplement 1.**
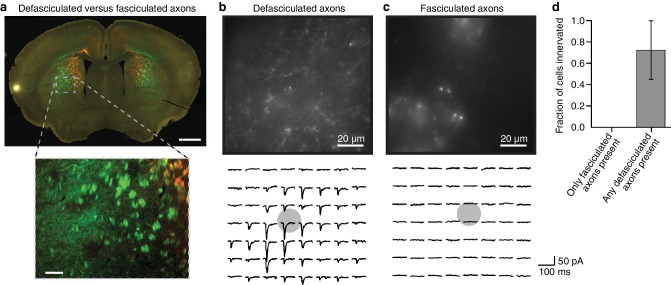
Fasciculated projection axons were excluded from striatal input maps because they do not form functional connections in the striatum. (**a**) An example coronal slice (top) depicting the two primary types of fluorescently labeled thalamostriatal axons in the striatum: defasciculated and fasciculated (zoomed image, bottom). (**b–c**) Top: High-magnification images of striatal areas containing (**b**) defasciculated and (**c**) fasciculated thalamostriatal axons expressing fluorescently tagged Channelrhodopsin (ChR2). Bottom: Current recordings of two example neurons recorded in the field of view shown in each image for (**b**) defasciculated and (**c**) fasciculated axons, showing synaptic currents elicited by light stimulation of thalamic axons. Gray circles indicate the locations of recorded neuronal cell bodies. Each current trace corresponds to the postsynaptic responses of optogenetic activation of the ChR2-expressing thalamostriatal axons using an 8 × 7 grid, 50 µm spacing blue light (as previously described (Hunnicutt et al., 2014; Mao et al., 2011) and briefly summarized in Materials and methods for details). (**d**) Summary results of whole cell recordings in portions of the striatum visually determined to possess either fasciculated axons, or defasciculated axons (mean ± SD, n = 4 slices for each condition, 4–5 cells per slice), and ChR2+ thalamic axons were activated with blue light laser stimulation. **DOI:** http://dx.doi.org/10.7554/eLife.19103.004

**Figure 1—figure supplement 2.**
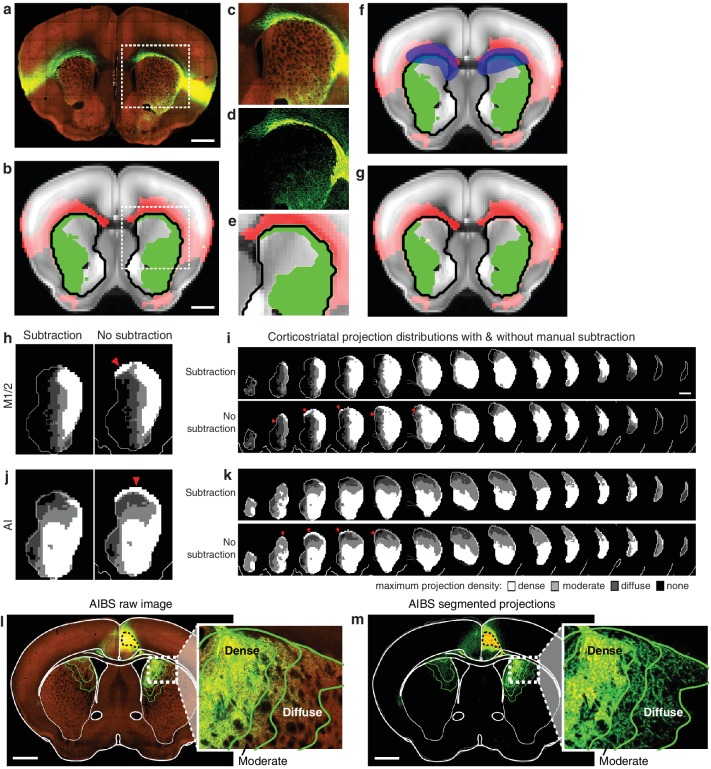
Illustration of method used to subtract traveling corticostriatal axons. (**a–g**) Example of manual processing required to remove aberrantly localized projections in the striatum resulting from traveling axons. (**a**) Fluorescent image of a coronal section showing projections from an injection in AI. (**b**) The striatal projection localization resulting from the image in panel **a** (green), overlaid on the AIBS average template brain (gray), showing projections outside the striatum (red), and the striatum outline (black line) (**c–e**) Axons in the corpus callosum aberrantly localized as striatal projections. The (**c**) fluorescent image, (**d**) segmented projection image, and (**e**) projection mask of voxels with >5% projection density (i.e. the moderate projection threshold) for the area indicated by a dashed box in panels **a** and **b**. (**f**) Manual mask (blue) created to subtract aberrantly localized projections. (**g**) Resulting striatal projection localization after subtraction (green). (**h–k**) Comparison of corticostriatal projection localization before and after subtraction. Example sections and full projection distributions in the striatum from (**h–i**) M1/2 and (**j–k**) AI with and without subtraction of traveling axons. Striatal section positions are the same as Figure 2b. (**l**) The same coronal brain section shown in Figure 1c with a more detailed view of the striatal projections for three projection density thresholds (inset). (**m**) The coronal section corresponding to panel **l**, showing only the binary segmented projections in the striatum with the approximate outline of the dense, moderate, and diffuse projections (green lines) defined for the voxelized projection data. **DOI:** http://dx.doi.org/10.7554/eLife.19103.005

**Figure 1—figure supplement 3.**
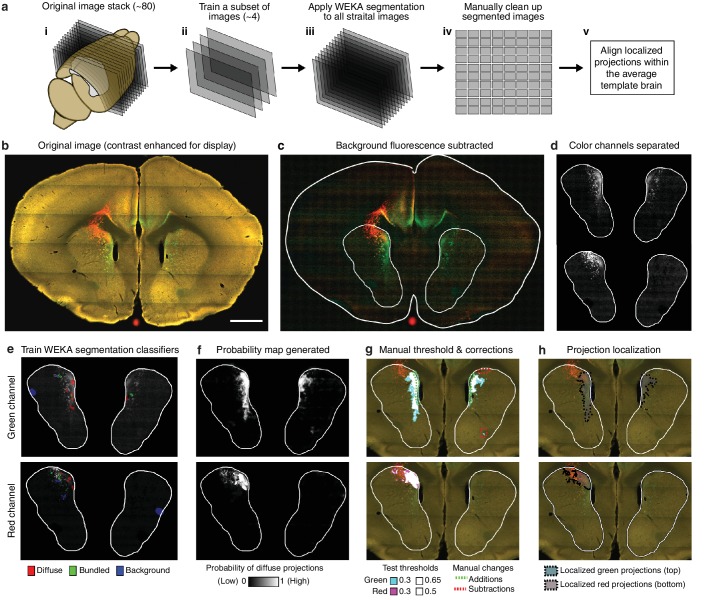
Overview of semi-automated image segmentation method for thalamostriatal projections. (**a**) Schematic overview of image segmentation method, wherein: (**i**) all coronal sections for each experimental brain containing striatum were analyzed (~80 sections per brain), (**ii**) a subset of four images were selected from the full image sets to train the Advanced WEKA Image Segmentation plugin in ImageJ to identify defasciculated projections, fasciculated projections, and background fluorescence, (**iii**) the trained Advanced WEKA Image Segmentation plugin was applied to the remaining images, (**iv**) segmented images were manually corrected, and (**v**) localized defasciculated projections were aligned within the average template brain (see Materials and methods). (**b–h**) More detailed example of the method described in **ii-iv** of panel a. (**b**) A fluorescent image of a coronal section through a mouse brain with fluorescent thalamic axons in the striatum (scale bar, 1 mm). (**c**) Image from panel b with background fluorescence subtracted. (**d**) Red and green color channels of the image are separated. (**e**) Advanced WEKA Image Segmentation is trained to identify defasciculated projections, fasciculated projections, and background fluorescence for each color channel separately. (**f**) The result of the segmentation method is a probability image displaying the probability that each pixel contains defasciculated projections; white represents the highest probability of 1 (note that the bright, fasciculated red axons on the left are excluded). (**g**) A probability threshold is manually chosen to encompass all defasciculated projections, and minor errors in the projection localization method are corrected manually. (**h**) The threshold was applied to the probability map and the manual changes were incorporated to determine the full distribution of defasciculated thalamic axons in the striatum for each color. **DOI:** http://dx.doi.org/10.7554/eLife.19103.006

**Figure 1—figure supplement 4.**
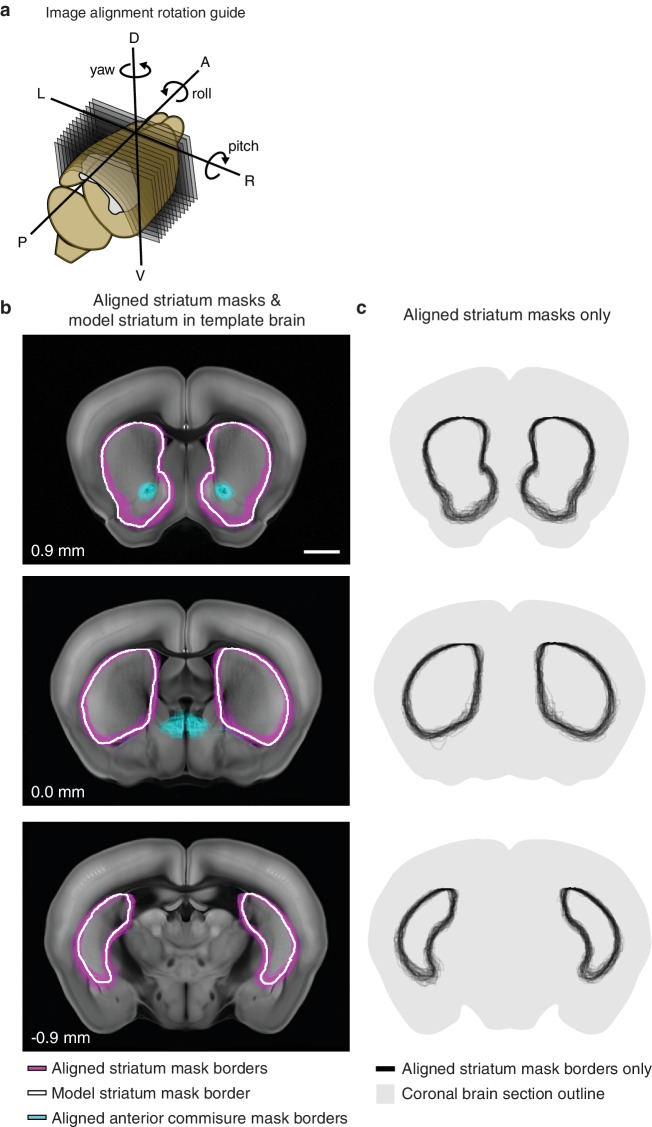
Striatum alignment for thalamic dataset. (**a**) Illustration depicting the rotations to be applied to striatum masks to align each experimental striatum in the thalamic dataset to the striatum of the AIBS average template brain. (**b**) Example coronal sections showing the outline of each experimental striatum after alignment (magenta), the outline of the striatum in the AIBS average template brain (white), and the outline of each experimental anterior commissure (acc) after alignment. Section positions indicated in mm relative to bregma. Scale bar 1 mm. (**c**) Coronal sections shown in panel B, showing all experimental striatum outlines. **DOI:** http://dx.doi.org/10.7554/eLife.19103.007

The localized dataset used in the current study includes a median of seven injections per subregion for 15 cortical subregions (Figure 1b and Supplementary file 1; see Table 1 for the list of all cortical subregions and their abbreviations). The projection distribution datasets for selected injections, which were aligned to the AIBS averaged template brain (Kuan et al., 2015), were acquired from AIBS as downsampled projection maps with a voxel size of 100 µm X 100 µm X 100 µm. Fluorescence signal in the striatum derived from fasciculated traveling axons, which did not form synaptic connections (Figure 1—figure supplement 1), was manually subtracted (Figure 1—figure supplement 2). The resulting dataset describes the full distribution of axonal projections in the striatum that originate from defined cortical subregions (Figure 1c–e). In addition to neocortical and mesocortical subregions, allocortical areas, including the amygdala (Amyg) and the hippocampal subiculum (Sub), were also included to obtain a comprehensive excitatory input map to the striatum (Figure 1a).

**Table 1.** Abbreviations. **DOI:** http://dx.doi.org/10.7554/eLife.19103.008

**Cortical plate derived subregions**			
**Abbreviation**	**Expanded name**	**AMBA Location**	**PMBA Location**
AI/GI/DI	agranular, granular, dysgranularinsular cortex	AI (all subregions) + GU + VISC	AI + DI + GI
Aud	auditory cortex	AUD (all subregions)	Au1 + AuD + AuV
Amyg	amygdala	BLA+ BMA	BLA + BMP
dACC	dorsal anterior cingulate cortex	ACAd	Cg1
FrA	frontal association cortex	FRP + MOs (bregma 2.4 to 3.1 mm)	FrA
IL	infralimbic cortex	ILA	IL
LO	lateral orbital cortex	ORBl	LO + DLO
M1/2	motor cortex	MO (all subregions, excluding FrA)	M1 +M2
MO	medial orbital cortex	ORBm	MO
Piri	piriform cortex	PIR	Pir
PrL	prelimbic cortex	PL	PrL
Ptl	parietal association cortex	PTL (all subregions)	MPtA + LPtA + PtPR + PtPD
Rhi	ecto-/peri-/ento-rhinal cortex	ECT + ENT + PERI	Ect + Ent + PRh + Lent
Rsp	retrosplenial cortex	RSP (all subregions)	RSA + RSG
S1/2	somatosensory cortex	SS (all subregions)	S1 (all subregions) + S2
Sub	hippocampal subiculum	SUB (all subregions) + CA1	S (all subregions)
Tem	temporal association cortex	TEa	TeA
vACC	ventral anterior cingulate cortex	ACAv	Cg2
Vis	visual cortex	VIS (all subregions)	V1 + All visual subregions
VO	ventral orbital cortex	ORBvl	VO
**Thalamic nuclei**			
**Abbreviation**	**Expanded name**	**AMBA Location**	**PMBA Location**
AD	anterodorsal nucleus	AD	AD
AM	anteromedial nucleus	AMd + AMv	AM + AMV
AV	anteroventral nucleus	AV	AV + AVDM + AVVL
CL	centrolateral nucleus	CL	CL
CM	centromedial nucleus	CM	CM
IAD	interanterodorsal nucleus	IAD	IAD
IAM	interanteromedial nucleus	IAM	IAM
IMD	intermediodorsal nucleus	IMD	IMD
LD	laterodorsal nucleus	LD	LD + LDVL + LDDM
LG	lateral geniculate nucleus	LG (LGd + LGv + IGL)	VLG + DLG + VLG + IGL
LP	lateral posterior nucleus	LP	LP + LPLR + LPMP + LPMC
MD	mediodorsal nucleus	MD (MDl + MDm + MDc)	MDC + MDL + MDM
MG	medial geniculate nucleus	MG (MGm + MGv + MGd)	MGD + MGV + MGM
PCN	paracentral nucleus	PCN	PC + OPC
Pf	parafascicular nucleus	PF	Pf
Po	posterior nucleus	PO	Po
PR	perireuniens nucleus	PR	vRe
PT	parataenial nucleus	PT	PT
PVT	paraventricular nucleus	PVT	PVA + PV
Re	reuniens nucleus	RE	Re
RH	rhomboid nucleus	RH	Rh
RT	reticular nucleus	RT	Rt
SM	submedius nucleus	SMT	Sub
VAL	ventral anterior-lateral complex	VAL	VA + VL
VM	ventromedial nucleus	VM	VM
VPL	ventral posterolateral nucleus	VPL + VPLpc	VPL + VPLpc
VPM	ventral posteromedial nucleus	VPM + VPMpc	VPM + VPMpc
**Other**	
**Abbreviation**	**Expanded name**
AIBS	Allen Institute for Brain Science
PMBA	Paxinos Mouse Brain Atlas
AMBA	AIBS Mouse Brain Atlas
DM	dorsomedial
D-V	dorsal to ventral
A-P	anterior to posterior
M-L	medial to lateral
NAc	nucleus accumbens
VL	lateral ventricle
Gpi	globus pallidus internal segment
SNr	substantia nigra pars reticulata
Gpe	globus pallidus external segment
cc	corpus callosum
MSN	medium spiny neurons

In contrast to the cortical subregions, certain thalamic nuclei are smaller than the typical size of a viral injection (Hunnicutt et al., 2014) and many of them have complex boundaries (Jones, 2007). To overcome this problem, we used a whole brain image dataset produced from 218 highly overlapping viral injections that covered >93% of the thalamic volume (Figure 1f) (Hunnicutt et al., 2014). The overlapping injections allow for the determination of the thalamic origins of projections in the striatum (Figure 1f–g) with finer resolution than the viral injection volume (Hunnicutt et al., 2014). Strong fluorescent signal derived from fasciculated axons that originate from the thalamus and travel through the striatum to reach their cortical targets was presented in the striatum (Figure 1g and Figure 1—figure supplement 1a). These axons do not form synaptic connections in the striatum, as confirmed by channelrhodopsin (ChR2)-mediated photostimulation experiments (Figure 1—figure supplement 1b–d), and therefore, their fluorescent signal needed to be removed. The fasciculated axons have distinct morphological features compared to the defasciculated axons which do form synaptic connections with medium spiny neurons (MSNs) in the striatum (Figure 1—figure supplement 1b–d). We applied a supervised machine learning algorithm to identify these morphological features and remove the fluorescent signal from fasciculated axons (Figure 1—figure supplement 3). The resulting striatal input maps were aligned to the AIBS averaged template brain (see Materials and methods and Figure 1—figure supplement 4), and thalamostriatal projections were localized using a semi-automated image segmentation method and custom-developed algorithms (Figure 1—figure supplement 4). The resulting dataset describes the axonal projection patterns in the striatum that originate from individual thalamic injections (Figure 1g–i).

### Corticostriatal input distribution patterns

Corticostriatal projections are known to have two functionally distinct types of innervation patterns: a core projection field of densely packed terminals and a larger diffuse (i.e. sparse) projection field (Haber et al., 2006; Mailly et al., 2013). To examine these different patterns, cortical projections within each downsampled striatal voxel were classified as one of three graded densities: dense, moderate, and diffuse projections, which were defined as over 20%, 5%, and 0.5%, respectively, of original imaging voxels containing fluorescent axons (Figure 1c–d and Materials and methods).

**Figure 2.**
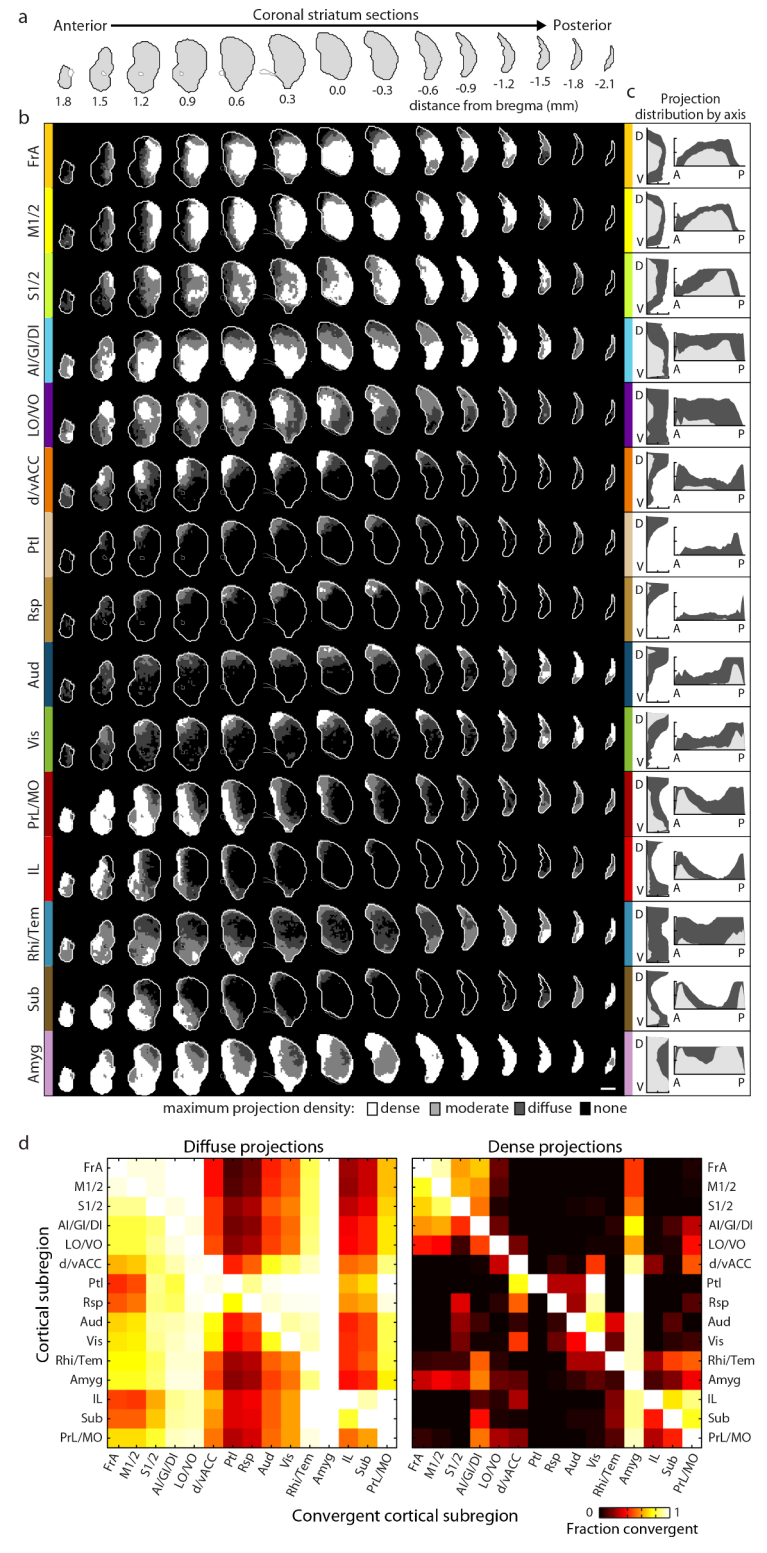
Comprehensive mapping of cortical inputs to the striatum. (**a**) Coronal section outlines for one hemisphere of the striatum (starting 1.8 mm anterior to bregma and continuing posterior in 300 µm steps, AIBS atlas). (**b**) Striatal projection distributions for all cortical subregions (rows). The maximum projection densities (dense (white), moderate (light grey), diffuse (dark grey), or none (black)) are indicated for the sum of all injections within each cortical subregion. (**c**) Projection distribution plots in the dorsal-ventral (D–V) and anterior-posterior (A–P) axes for each cortical subregion shown in b. Coverage in the striatum by dense (light gray) and diffuse (dark gray) projections were calculated in 100 µm steps as the fraction of the striatum covered in each step by either dense or diffuse projections, respectively. Striatal volumes were normalized in each 100 µm step. (**d**) Subregion-specific convergence plots for diffuse (left panel) and dense (right panel) corticostriatal projections. The color scale indicates the fraction of the projection field from a given cortical subregion (rows) covered by the projection field from another cortical subregion (columns). **DOI:** http://dx.doi.org/10.7554/eLife.19103.009

For each cortical subregion, a maximum projection density map throughout the striatum was determined by combining projection distributions from all injections within a given cortical subregion (Figure 2a–c and Figure 2—figure supplement 1). Each projection distribution was quantified in the dorsal-ventral (D-V), anterior-posterior (A-P) (Figure 2c), and medial-lateral (M-L) (data not shown) axes. Each cortical subregion gave rise to a distinct projection pattern in the striatum, forming either one or two contiguous volumes. While no two projection maps were identical, some were similar. For example, the somatosensory and motor subregions, including FrA, M1/2, and S1/2, exhibit similar projection patterns, producing dense, highly overlapping projection fields in a large volume of the central portion of the striatum in the A-P axis. These projections were biased toward the lateral striatum, likely including the traditionally-termed dorsolateral domain (Figure 2b–c). In contrast, frontal subregions, including LO/VO, IL, and MO/PrL, have smaller, largely non-overlapping dense projections in the anterior, medial striatum and diffuse projections that span a larger striatal volume (Figure 2b–c). When injections were further grouped according to their locations in either the A-P or M-L axis, we observed a moderate trend for topographic organization in the A-P and M-L axes for the dense projections, but this was not seen for diffuse projections (Figure 2—figure supplement 2). Nevertheless, even the dense projections from such grouping often resulted in several discrete, non-contiguous projection fields (Figure 2—figure supplement 2b and e), which are not as well defined as the cortical subregion-specific projection fields, as shown in Figure 2b.

**Figure 2—figure supplement 1.**
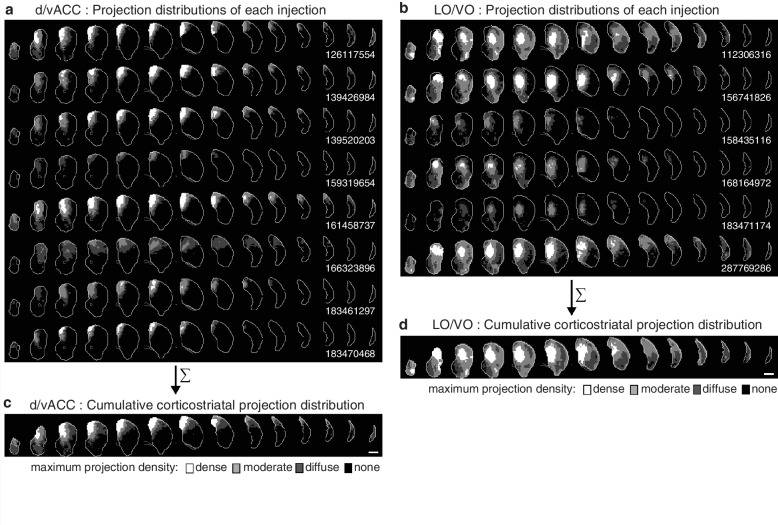
Cumulative corticostriatal projection distributions. (**a–b**) Projection distributions in the striatum from viral injections in two example cortical subregions: (**a**) dv/ACC and (**b**) LO/VO, respectively. (**c–d**) The final cumulative projection distributions for (**c**) d/vACC and (**d**) LO/VO, created by maximum projection of the density at each striatal voxel from all individual injections in each cortical subregion. **DOI:** http://dx.doi.org/10.7554/eLife.19103.010

**Figure 2—figure supplement 2.**
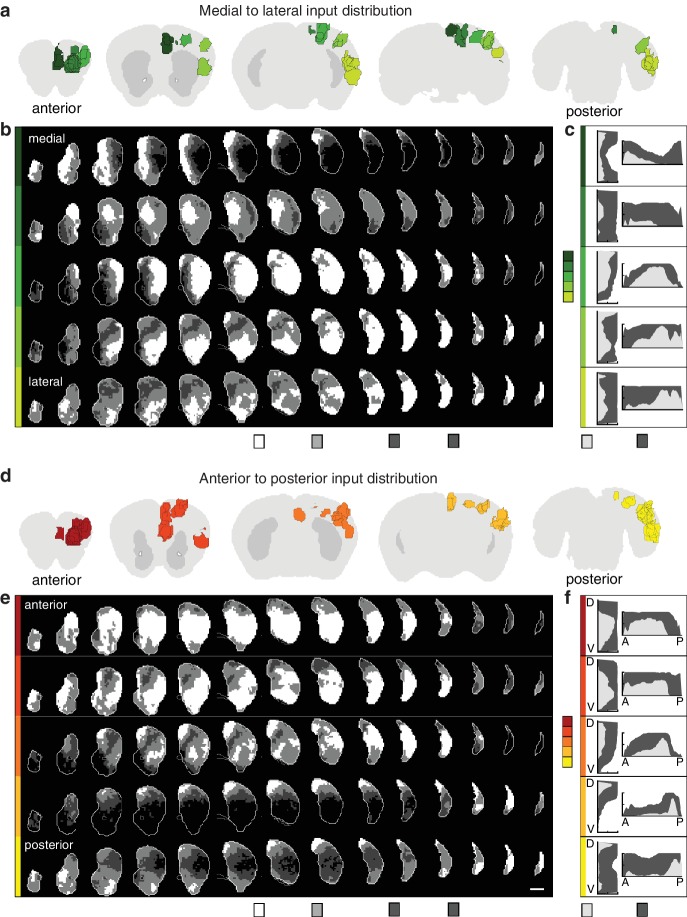
Topographic organization of corticostriatal inputs. (**a**) Coronal sections (2.7, 0.9, –0.9, −3.3, and −4.5 mm relative to bregma) through the AIBS averaged template brain showing the relative locations of injections in each of five medial to lateral (M–L) groups (dark to light green). (**b**) Coronal sections (starting 1.8 mm anterior to bregma and continuing posterior in 300 µm steps) through the ipsilateral striatum showing the striatal projection distributions for the M-L injection groups shown in panel **a** (rows). The maximum projection densities (dense (white), moderate (light grey), diffuse (dark grey), or none (black)) are indicated for the sum of all injections within each group. (**c**) Distribution plots for the dense (light gray) and diffuse (dark gray) projections of each M-L cortical group shown in panel **b** (calculated as described in Figure 2). (**d**) Coronal sections (starting at 2.7, 1.5,–0.3, −2.1, and −4.5 mm relative to bregma) of the AIBS averaged template brain showing the relative locations of injections in each of five anterior to posterior (A–P) groups (red to yellow). (**e**) Coronal sections (as described in panel **b**) of the ipsilateral (to the injection side) striatum showing the striatal projection distributions for the A-P injection groups (rows, marked in colors corresponding to panel **d**). The maximum projection densities (dense (white), moderate (light grey), diffuse (dark grey), or none (black)) are indicated for the sum of all injections within each group. (**f**) Distribution plots for the dense (light gray) and diffuse (dark gray) projections of each cortical group shown in panel **e** (calculated as described in Figure 2, shown in D-V and A-P axes). **DOI:** http://dx.doi.org/10.7554/eLife.19103.011

Next, to reveal whether information from different cortical subregions may interact in the striatum, we calculated pairwise projection convergence between cortical subregions for diffuse and dense projections (Figure 2d). As expected, the diffuse projections have a higher degree of convergence than the dense projections; however, we identified cortical areas that showed very little convergence, even for diffuse projections. For example, Ptl, Rsp, IL, and Sub have very little projection overlap with the motor areas M1/2 or FrA. In contrast, several cortical subregions, such as the motor (FrA and M1/2) and select sensory (S1/2 and AI/GI/DI) subregions, have a high level of convergence for both diffuse and dense projections (Figure 2d).

### Thalamostriatal inputs and their convergence with corticostriatal inputs

**Figure 3.**
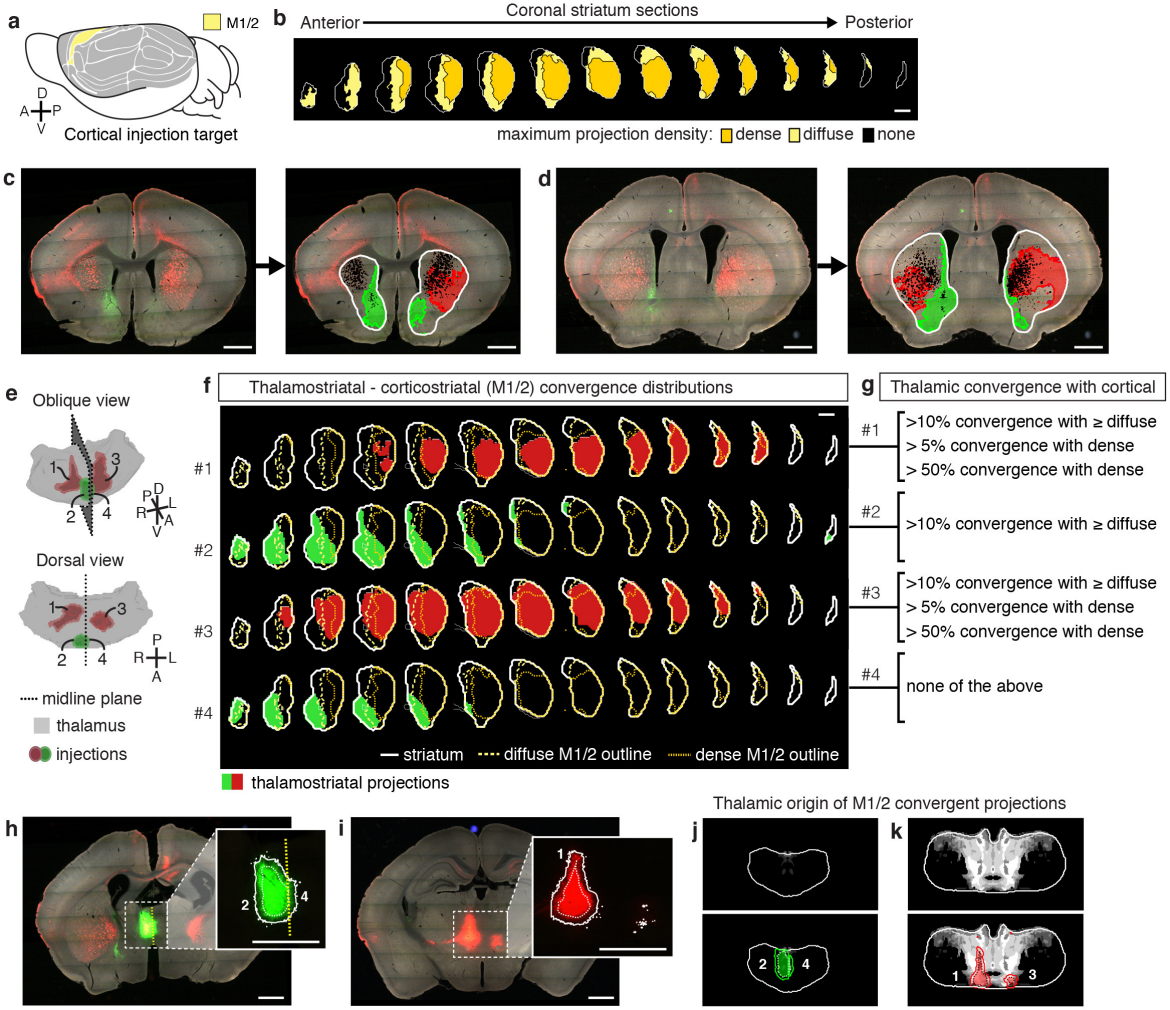
Localization of the origins of thalamostriatal projections that converge with a corticostriatal projection in the striatum. (**a**) Schematic sagittal view of the mouse brain, adapted from (Watson et al., 2012), indicating the location of M1/2. (**b**) Distribution of dense (dark yellow) and diffuse (light yellow) corticostriatal projections from M1/2. (**c–d**) Representative images of two coronal sections through the striatum of one example brain (left panels in **c** and **d**) showing the fluorescent thalamic axons in the striatum from injections described in panel **e**. Original images are on the left and segmented striatum and axon projection fields are on the right, with traveling axon bundles subtracted (black in right images). (**e**) Two views of a model thalamus (gray) showing the four thalamic viral injections that produced projections shown in panels **c** and **d**. Note that since thalamic projections do not cross the midline in mouse, a single injection spanning the midline was treated as two independent injections (injections 2 and 4). A darker center of each injection site represents the eroded ‘core’ of each injection defined previously for the thalamic injection dataset (Hunnicutt et al., 2014). (**f**) Projection distributions in the striatum for each of the injections shown in panel **e** (red and green) aligned and overlaid with the outlines of M1/2 projections in the striatum (yellow) delineated in panel **b**. (**g**) Injections were assigned to one or more of four categories based on quantification of the convergent volumes of thalamostriatal and corticostriatal projection fields (see Materials and methods). Inclusion in each category is used, as described in Figure 3—figure supplement 1, to localize the thalamic origins of convergence. (**h–i**) Fluorescent images of coronal sections through the thalamus, showing injection sites 1, 2, and 4. Insets show the segmented injection sites (solid white line) and the injection site core (dashed white line) (Hunnicutt et al., 2014). The dashed yellow line in panel **h** insert shows the brain midline. (**j–k**) Two example coronal sections, approximately corresponding to the position of panels **h** and **i**, respectively, of the thalamostriatal confidence maps for M1/2 convergence in panels **h** and **i**, respectively (top panels in **j** and **k**). The segmented injection sites are overlaid on their corresponding confidence maps (bottom panels in **j** and **k**). All scale bars, 1 mm. **DOI:** http://dx.doi.org/10.7554/eLife.19103.012

**Figure 4.**
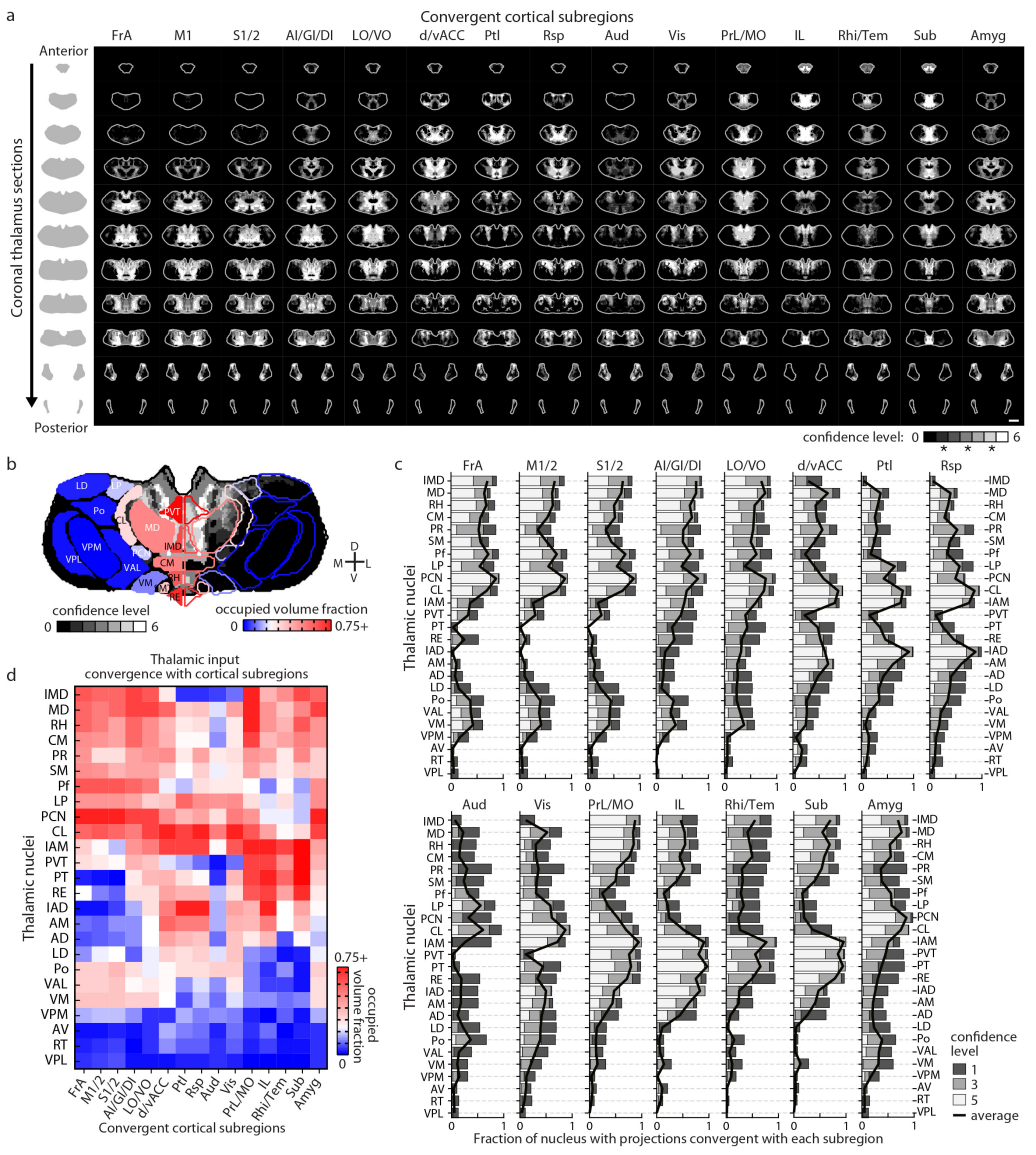
Thalamostriatal projections that converge with subregion-specific corticostriatal projections. (**a**) Example coronal sections through our model thalamus from anterior to posterior (starting at −0.155 mm relative to bregma and continuing in 250 µm steps posterior). Confidence maps identifying the complete thalamic origins of thalamostriatal projections that converge with subregion-specific corticostriatal projections (columns). Projection origins indicated for six confidence levels (see Materials and methods, and also see [Hunnicutt et al., 2014]). (**b**) An example coronal section of the thalamostriatal confidence map converging with corticostriatal inputs of Sub (gray scale), overlaid with thalamic nuclear demarcations from the AMBA. The atlas is colored on the left to indicate the fraction of each thalamic nucleus covered by the average of confidence levels 1, 3, and 5. Coverage values were calculated for the PMBA and AMBA, and their average is shown. The color scale minimum is 0% (blue), inflection point is 25% (white), and the peak coverage is 75% (red). (**c**) The fraction of each thalamic nucleus covered by confidence levels 1, 3, and 5 (dark, medium and light gray bars, respectively), with their average (black line). (**d**) Aggregate nucleus coverage map indicating the nuclear origins of the thalamostriatal projections that converge with subregion-specific corticostriatal projections. Nuclei (rows) and cortical subregions (columns) are hierarchically clustered on the basis of output and input similarity, respectively. Color scale is the same as in panel **b**. **DOI:** http://dx.doi.org/10.7554/eLife.19103.014

To localize the projection distribution for each thalamic injection, we developed a semi-automated image segmentation method to identify axonal projections in the striatum (Figure 1f–h, Figure 3c–d, Figure 1—figure supplement 3, and Materials and methods). To compare thalamostriatal projection patterns across animals, as well as to corticostriatal projections, the segmented striatum for each experiment was computationally aligned to the AIBS averaged template brain (Figure 1d and Figure 1—figure supplement 4). Similar to our previous study of thalamocortical projections (Hunnicutt et al., 2014), the thalamic injections were individually categorized based on their projections to a given striatal volume, such as a striatal volume innervated by a specific cortical subregion (Figure 3g and Figure 3—figure supplement 1a). Figure 3 shows a representative example, wherein four thalamic injections are categorized based on their projection convergence with M1/2 projections in the striatum. Injections found to fulfill each category were combined and then used to derive the thalamic confidence map for the striatal subregion innervated by M1/2 (Figure 3g–k, Figure 4 and Figure 3—figure supplement 1; see Materials and methods for details). Each thalamic confidence map describes the likelihood that a given thalamic volume projects to a given striatal volume with a resolution finer than the size of individual injections (Figure 3—figure supplement 1). This process was repeated for all cortical subregions, producing a complete map of striatal convergence for corticostriatal and thalamostriatal projections (Figure 4a).

**Figure 3—figure supplement 1.**
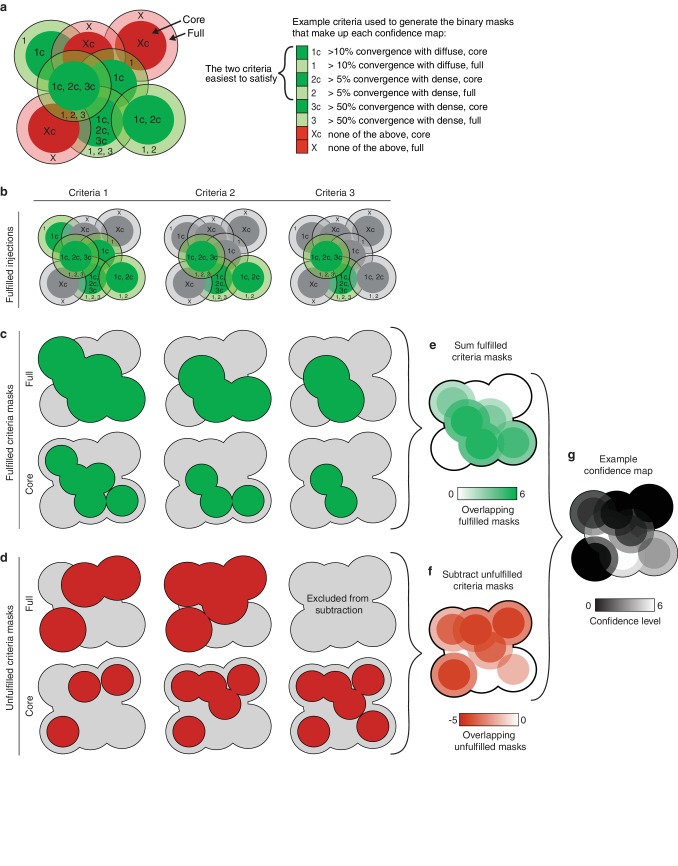
Illustration of the method used to generate thalamic confidence maps. (**a**) Illustration of eight hypothetical injection volumes labeled to indicate whether or not they individually satisfy each of three criteria for their projections in the striatum (right). Each injection indicates the borders of the full injection (1, 2, 3, and X) and the injection core (1c, 2c, 3c, and Xc), which is generated by eroding the full injection volume by 100 µm. (**b**) Injections shown in panel a, indicating the injections that fulfill each of the three criteria (green). (**c**) Binary injection masks representing the area covered by either the full injection (top) or injection cores (bottom) that satisfy each of the three criteria. (**d**) Binary injection masks representing the area covered by either the full injection (top) or injection cores (bottom) that do not satisfy each of the three criteria. Binary mask for the full injections that do not meet the hardest to satisfy criteria are not included. (**e**) Sum of binary masks generated in panel **c**. (**f**) Subtraction of the binary masks generated in panel d. (**g**) Example confidence map, generated by combining the summed binary masks from panel E and the subtracted binary masks from panel f. Values below zero set to zero. **DOI:** http://dx.doi.org/10.7554/eLife.19103.013

We further determined the thalamic nuclear origins of the thalamostriatal projections by overlaying confidence maps with the two widely used mouse atlases (the AMBA and the Paxinos Mouse Brain Atlas (PMBA) [Paxinos and Franklin, 2001]). The coverage of each atlas-outlined nucleus was calculated for each confidence level (Figure 4b–d). Of the thalamic subregions covered in this dataset, all thalamic nuclei, except the anteroventral nucleus (AV), reticular nucleus (RT), ventral posteromedial nucleus (VPM), and ventral posterolateral nucleus (VPL), project to the striatum (Figure 4c–d) (Hunnicutt et al., 2014). Overall, overlapping, yet distinct, thalamic subregions converge in the striatum with each cortical subregion (Figure 4a and d).

### Distinct input convergence between striatal subdivisions

To determine if and how different portions of the striatum exhibit heterogeneity in the excitatory inputs they receive, the dense and diffuse corticostriatal projections (as illustrated in Figure 2a–c) were summed, respectively, across all cortical subregions (Figure 5—figure supplement 1). The results indicate that distinct striatal subdivisions receive different numbers of converging cortical inputs and that there are distinct differences between dense and diffuse projection convergence (Figure 5—figure supplement 1a–b). Nearly all striatal voxels receive diffuse projection from at least five cortical subregions, with an average of 8.3 cortical inputs converging per voxel. When the striatal voxels were subdivided based on the average convergence level, two distinct subdivisions formed. A large, contiguous subdivision, constituting ~63% of the ipsilateral, is innervated by diffuse projections from a high number of cortical subregions (10.7 ± 1.1 inputs per voxel, mean ± s.d.), and a second subdivision receiving diffuse projections from a low number of cortical subregions (6.6 ± 0.84 inputs per voxel, mean ± s.d.) (Figure 5—figure supplement 1a–b). Interestingly, when we constructed thalamic confidence maps to localize the thalamic subregions innervating the ipsilateral striatum receiving either a high (>8.3 inputs) or a low (≤8.3 inputs) level of cortical convergence, the striatal subdivision with high cortical input convergence was found to receive inputs from every thalamic nucleus shown to project to the striatum (Figure 5—figure supplement 1c). In contrast, the striatal subdivision with low input convergence does not receive any input from the anterior thalamic nuclei (Figure 5—figure supplement 1c). For dense corticostriatal projections, a lower level of convergence was observed (2.7 ± 0.4 inputs per voxel, mean ± s.d.), as expected since dense projections cover a smaller volume. However, their convergence exhibits a different distribution pattern from that of the diffuse projections (Figure 5—figure supplement 1a). For example, a higher level of convergence of the diffuse projections is observed in the dorsal striatum, whereas dense projection convergence is biased toward the ventral striatum (Figure 5—figure supplement 1a).

To investigate whether the striatal subdivisions with either high or low cortical input convergence (Figure 5—figure supplement 1a) could be attributed to evolutionary differences in the cortical inputs, we mapped the projection distributions for the evolutionarily distinct classes of the cortical plate: neocortex, mesocortex, and allocortex (Figure 5—figure supplement 2), which carry predominantly sensory/motor, associative, and limbic information, respectively (McGeorge and Faull, 1989). We found that, instead of a single class, the striatal subdivision with high cortical convergence always received input from multiple cortical classes (Figure 5—figure supplement 2a). Additionally, the thalamostriatal inputs that converge with each cortical class (Figure 5—figure supplement 2b–c) did not mimic the thalamostriatal inputs that converge with striatal subdivisions based on high/low input convergence (Figure 5—figure supplement 1b–c). These results provide evidence for multimodal input integration throughout the striatum and functional heterogeneity between striatal areas having distinct diffuse and dense input convergence.

### Defining striatal subdivisions based on excitatory input patterns

**Figure 5.**
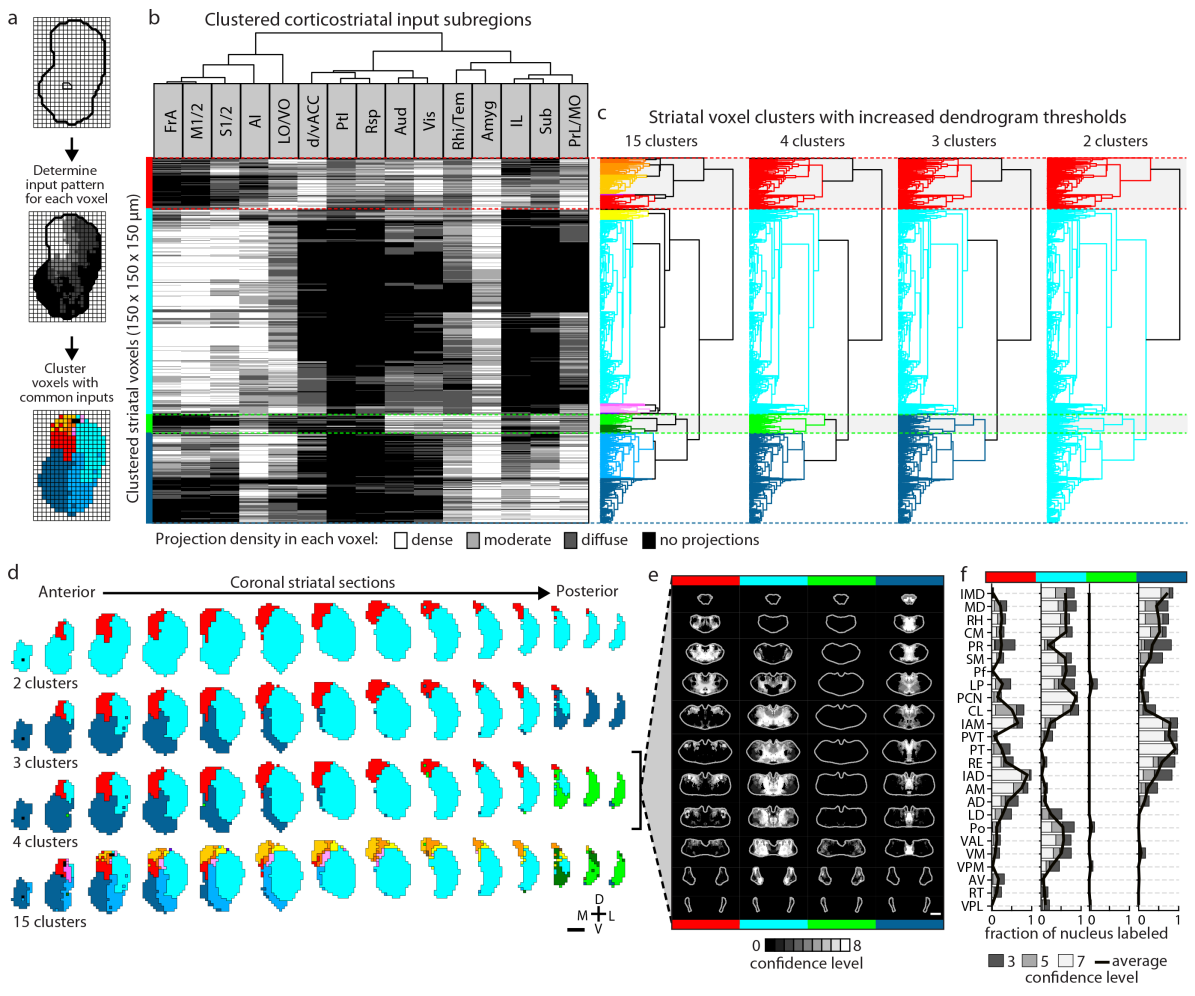
Striatal subdivisions based on cortical input convergence. (**a**) Schematic of voxel clustering method. The striatum was downsampled into 150 µm × 150 µm × 150 µm voxels (top panel), the projection density (dense, moderate, or diffuse) to each voxel was determined for inputs from each cortical subregion (middle panel), and the sum of this information was used to cluster voxels with common inputs (bottom panel). (**b**) All striatal voxels (rows) were hierarchically clustered based on their cortical input patterns, and cortical subregions (columns) were clustered based on common innervation patterns to the striatum. The projection densities in each voxel are indicated in gray scale, as determined in Figure 2b. (**c**) Four separate thresholds were applied to the voxel dendrogram to produce 2, 3, 4, and 15 clusters. The cluster boundaries (dotted color lines) for the threshold producing four clusters are carried across the clustered voxels in panel **b**. Clusters containing only one voxel were ignored in our analyses. (**d**) Coronal sections outline the ipsilateral (according to the injection hemisphere) striatum, starting 1.8 mm anterior to bregma and continuing posterior in 300 µm steps, showing the spatial location of the clusters determined in panel **c**. (**e**) Thalamic confidence maps indicating the thalamic origins of thalamostriatal projections to the four striatal subdivisions defined by cluster analysis in panel **d** (thalamic section positions are the same as in Figure 4a). The method used to localize the origin of thalamic projections was similar to that described for Figures 3 and 4, except that differences in the data resulted in an eight level confidence maps based on the inclusion of each injection in each of four groups (see Materials and methods). (**f**) The fraction of each thalamic nucleus covered by confidence levels 3, 5, and 7 (dark, medium and light gray bars, respectively), with their average (black line) shown for the confidence maps in panel **e** (see Figure 5—figure supplement 3 for full dataset, and Materials and methods for details). **DOI:** http://dx.doi.org/10.7554/eLife.19103.015

The striatum is the largest part of the telencephalon without clearly demarcated subdivisions. Since the above analyses indicate heterogeneity in excitatory input integration across the striatum (Figure 2 and Figure 5—figure supplement 1), and cortical input patterns are thought to be stereotypic across animals, we sought to subdivide the striatum using an objective and functionally relevant approach based on corticostriatal projection patterns. The striatum was downsampled to a voxel size of 150 µm x 150 µm x 150 µm, and the projection density within each voxel was calculated for all cortical inputs (Figure 5a). The voxels, each treated independently, were clustered based on the input density (none, diffuse, moderate, or dense, as illustrated in Figure 2) they received from all cortical subregions (Figure 5b–c and Materials and methods). Cortical subregions were analogously clustered based on their projections to individual striatal voxels (Figure 5b). To identify striatal subdivisions in an unbiased manner, four increasingly lower thresholds were applied to the voxel clustering dendrogram to generate voxel groups (Figure 5c). Each voxel group was then mapped back onto the striatum (Figure 5d). Notably, although no positional information was used in the clustering analysis, the resulting voxel clusters form largely contiguous volumes (Figure 5d), suggesting that these voxel clusters may represent functionally distinct subdivisions.

**Figure 5—figure supplement 1.**
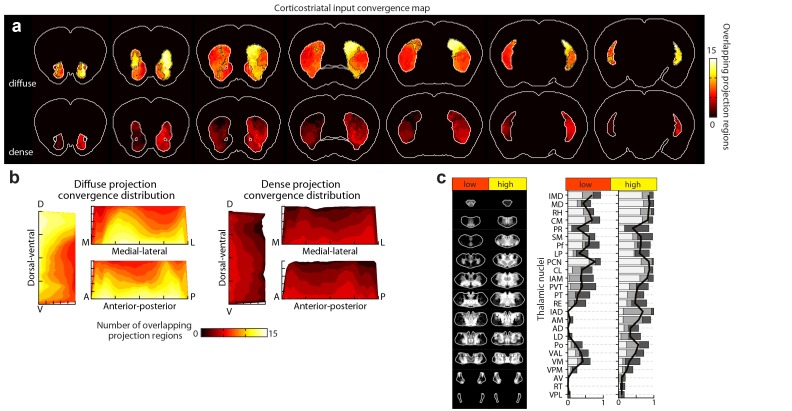
Distribution of cortical input convergence in the striatum. (**a**) Coronal sections of the average template brain showing the cumulative bilateral convergence of diffuse (top) and dense (bottom) projections from all cortical subregions (heat map). The striatal areas with convergent inputs from nine or more cortical subregions are indicated (black dashed line). Sections start 1.8 mm anterior to bregma, the second slice is 300 µm posterior, and the rest continue in 600 µm steps. (**b**) Projection distribution plots in the dorsal-ventral (D–V), medial-lateral (M–L) and anterior-posterior (A–P) axes for diffuse (left panel) and dense (right panel) input convergence. Coverage of cortical inputs in the striatum by the indicated number of cortical subregions was calculated in 100 µm steps along each axis. The fraction of the striatum covered in each step by each number of converging projections is shown as a heat map, where each plot is collapsed to show only the dimension indicated (i.e. the D-V plot does not contain any M-L or A-P information). Striatal volumes were normalized in each 100 µm step. (**c**) Left panel: summary of thalamic confidence maps for the origins of thalamostriatal projections that target striatal volumes with high- and low-diffuse cortical projection convergence, as determined in panel **a** (thalamic section positions are the same as in Figure 4a). Right panel: the fraction of each thalamic nucleus covered by confidence levels 3, 5, and 7 (dark, medium and light gray bars, respectively), with their average (black line) (see Materials and methods and Figure 5—figure supplement 1). **DOI:** http://dx.doi.org/10.7554/eLife.19103.016

**Figure 5—figure supplement 2.**
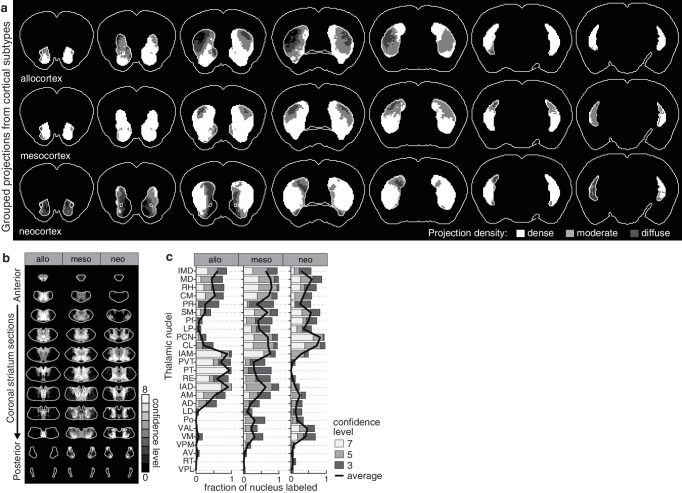
Projection distribution and thalamic input convergence for cortical subtypes. (**a**) Coronal sections of the model brain showing the bilateral distributions of dense, moderate and diffuse projections from all allocortical (top), mesocortical (middle), and neocortical (bottom) subregions. The second slice is 300 µm posterior to the first slice, continuing in 600 µm steps. (**b**) Thalamic confidence maps for the origins of thalamostriatal projections that converge in the striatum with projections from each cortical subtype, as determined in panel **a** (section positions are the same as in Figure 4a). (**c**) The fraction of each thalamic nucleus covered by confidence levels 3, 5 and 7 (dark, mid and light gray bars, respectively), and their average (black line). **DOI:** http://dx.doi.org/10.7554/eLife.19103.017

**Figure 5—figure supplement 3.**
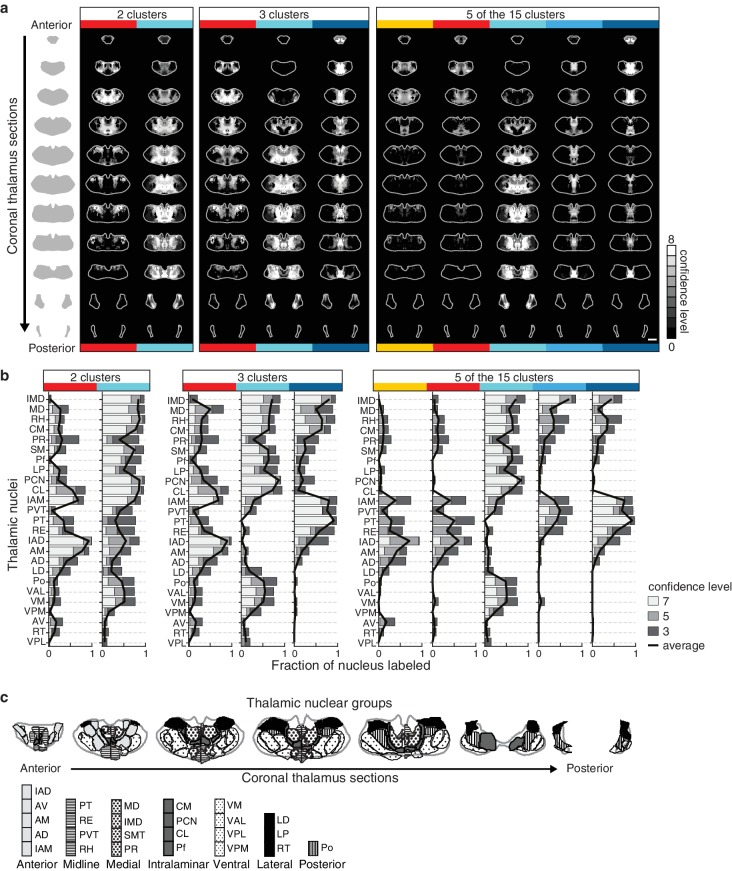
Thalamic origins of inputs to striatal clusters. (**a**) Coronal sections through the thalamus from anterior to posterior. Thalamic confidence maps indicating the origins of thalamostriatal projections to the striatal voxel clusters shown in Figure 5. Confidence maps are shown for the origins of all projections to each of the two clusters (left), three clusters (middle), and the 5 largest of the 15 clusters (right) thresholds (grayscale, section positions are the same as in Figure 4a). (**b**) The fraction of each thalamic nucleus covered by confidence levels 3, 5, and 7 (dark, mid, and light gray bars, respectively), with their average (black line) is shown for the confidence maps in panel a (see Materials and methods for details). (**c**) Coronal sections of the thalamus showing the boundaries of the canonical thalamic nuclear groups (anterior, midline, medial, intralaminar, ventral, lateral, and posterior). In each section, the nuclear boundaries are shown for both the PMBA (left) and the AMBA (right), which were previously aligned to the thalamic dataset used there (Hunnicutt et al., 2014). **DOI:** http://dx.doi.org/10.7554/eLife.19103.018

**Figure 5—figure supplement 4.**
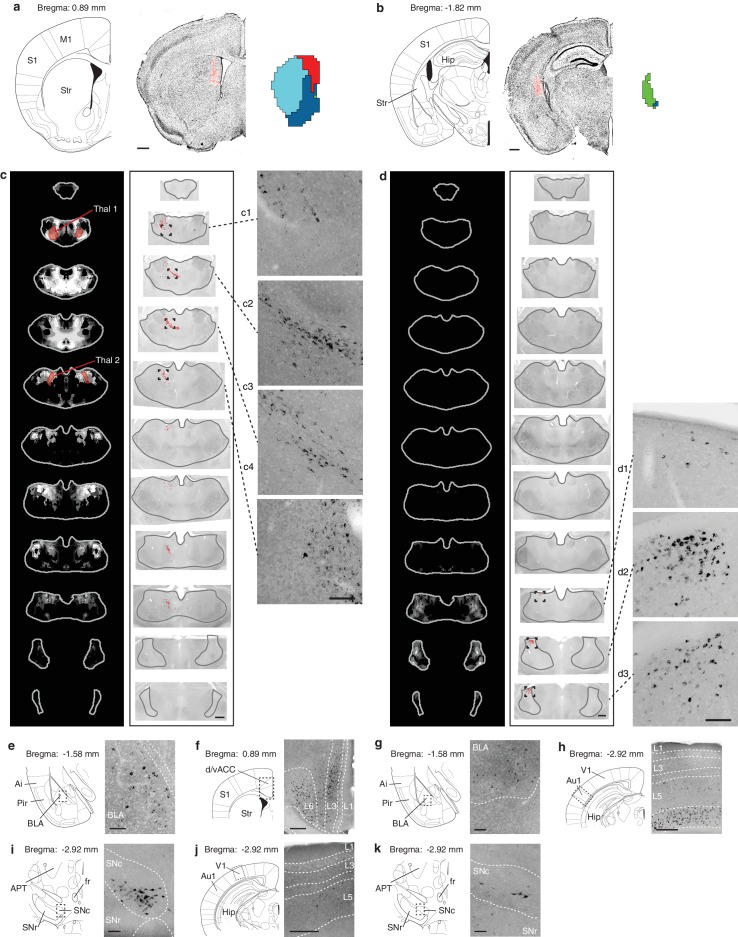
Retrograde verification of anterograde projection maps. (**a–b**) Representative injection sites of Lumafluor retrograde beads in the dorsomedial (DMS, **a**) and posterior striatum (PS, **b**). Left, reference coronal section from PMBA; center, immunostained sections (gray, inverted lookup table) with Lumafluor beads (red); and right, the striatal subdivisions (DMS, red; PS, green) based on Figure 5. (**c–k**) Retrograde-labeled cells in the thalamus, midbrain, and several other subcortical regions, as indicated after injection of DMS (**c, e, f, i, j**) and PS (**d, g, h, k**). (**c**–**d**) Left, thalamic confidence maps indicating the thalamic origins of the thalamostriatal projections to DMS and PS, as shown in Figure 5. Thal1 and Thal2, red, were used for optogenetic stimulation of thalamostriatal projections to the DMS (Figure 8); center, corresponding coronal sections (gray, inverted lookup table) of the thalamus with retrograde-labeled cells (red dots) and thalamus outline (grey line); right, enlarged raw images corresponding to the boxed areas in center. (**e**–**k**) Retrograde labeled observed in the basolateral amygdala (BLA, **e, g**), dorsal/ventral anterior cingulate cortex (d/vACC, **f**), primary auditory cortex (Au1, **h**), *substantia nigra pars compacta* (SNc, **i, k**), and primary visual cortex (V1, **j**). Left, reference sections from PMBA, right, immunostaining of retrograde-labeled cells (gray, inverted lookup table). Ai, agranular insular cortex; APT, anterior pretectal nucleus; fr, *fasciculus retroflexus*; Hip, hippocampus; L1-6, cortical layer 1–6; Pir, piriform cortex; S1, primary somatosensory cortex; SNr, *substanta nigra reticulare*; Str, striatum. Scale bars: **a**–**d**, 500 µm; **c1**–**c4** and **d1**–**d3**, 100 µm; **e**, **g**, **i**, **k**, 100 µm; **f**, **h**, **j**, 250 µm. **DOI:** http://dx.doi.org/10.7554/eLife.19103.019

The highest dendrogram threshold divided the striatum into two clusters, separating a small dorsomedial subdivision from the rest of the striatal volume (Figure 5c–d). A slightly lower threshold produced three clusters that are highly reminiscent of the three traditional striatal domains: a dorsomedial subdivision with highly convergent inputs, a lateral subdivision receiving dense sensorimotor innervation, and a ventral subdivision receiving several limbic inputs (Figure 5b–d). Notably, the ventral subdivision contains two non-contiguous segments: a ventral segment in the anterior striatum and the most posterior segment of the striatum, suggesting that they may represent two different domains. Indeed, when the threshold was lowered to create a fourth cluster, the posterior segment became a distinct cluster (Figure 5d). Although this posterior subdivision shares similarities with the limbic domain, it also receives strong auditory and visual innervation (Figure 5b). This posterior cluster may constitute a previously unappreciated functional subdivision of the striatum in mice. Further lowering the threshold to produce 13 clusters divided the dorsomedial subdivision, as well as a small portion of the lateral subdivision immediately adjacent to the dorsomedial subdivision, into many smaller clusters without dividing the remaining three subdivisions (see the small clusters at the dorsomedial striatum in the bottom row of Figure 5d), indicating a high degree of input heterogeneity in this region. When the threshold was lowered to produce 15 clusters, the posterior and ventral subdivisions are each separated into two clusters, where one cluster receives motor and somatosensory information and the other cluster does not (Figure 5b–d). Importantly, even with the low threshold generating 15 clusters, the majority of the lateral subdivision, likely corresponding to the traditional sensorimotor domain, remained as a single large cluster, suggesting a highly homogeneous functional role for this region.

We also determined the origins of thalamic inputs to each cluster-defined striatal subdivision (Figure 5e–f, and Figure 5—figure supplement 3). Each striatal cluster, although defined by cortical inputs, receives innervations from distinct thalamic subregions (Figure 5e and Figure 5—figure supplement 3a). The thalamic inputs largely project to striatal clusters in accordance with the known thalamic nuclear groups (Figure 5f and Figure 5—figure supplement 3b–c). For example, when the striatum is divided into four clusters (Figure 5e–f), the dorsomedial subdivision receives input primarily from the anterior nuclear group, the ventral subdivision receives most of its inputs from the midline and medial nuclear groups, the lateral subdivision receives inputs from the ventral, intralaminar, posterior, and medial nuclear groups, while the posterior subdivision receives only weak thalamic input from the lateral posterior nucleus (LP) (Figure 5f and Figure 5—figure supplement 3b–c). To verify that the convergent inputs to each subdivision were accurately localized, retrograde bead injections were performed in portions of the dorsomedial and posterior subdivisions (Figure 5—figure supplement 4a–b). All cortical and thalamic subregions labeled by the retrograde injections were predicted by our dataset (Figure 5—figure supplement 4c–h). The unique cortical and thalamic input patterns to different striatal clusters suggest that each cluster may serve distinct functions.

### Circuit properties of the cortico-thalamo-basal ganglia loop

**Figure 6.**
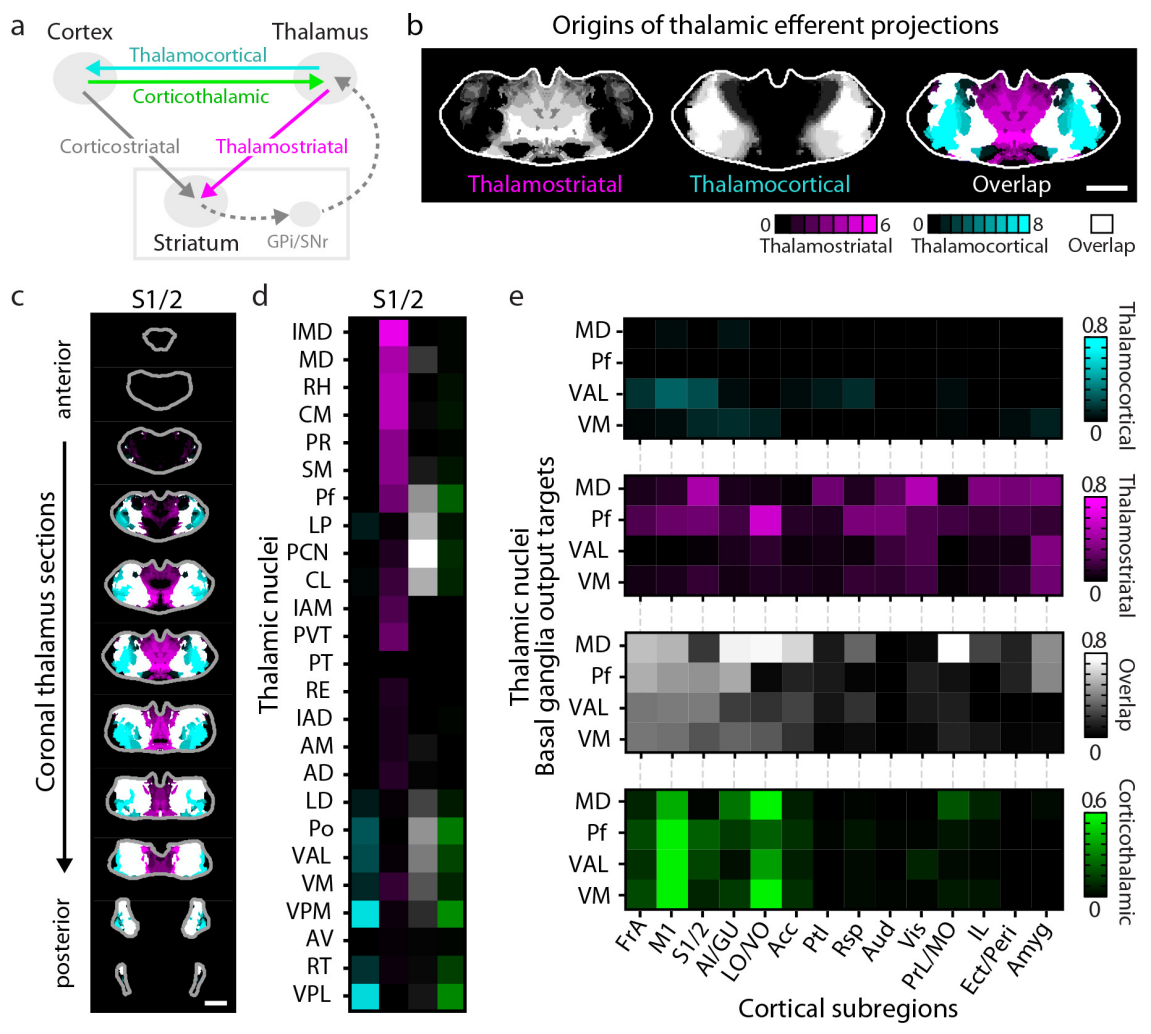
Connectivity of excitatory projections in the cortico-thalamo-basal ganglia circuit. (**a**) Schematic of the excitatory connections between the cortex, thalamus, striatum, and the output nuclei of the basal ganglia, globus pallidus internal segment (GPi) and substantia nigra pars reticulata (SNr), which collectively make up the cortico-thalamo-basal ganglia circuit (gray box indicates the basal ganglia). (**b–d**) Example connectivity matrix for one part of the cortico-thalamo-basal ganglia circuit. (**b**) Confidence map showing the origins of thalamostriatal projections that converge with projections from somatosensory cortex (S1/2) (left), and confidence maps for the origins of thalamocortical projections that terminate in S1/2 (center, previously published data, [Hunnicutt et al., 2014]), with their overlay shown on the right (thalamostriatal: magenta; thalamocortical: cyan; overlap: white). (**c**) Overlaid thalamocortical and thalamostriatal confidence maps, as described in panel **b** (thalamic section positions are the same as in Figure 4a). (**d**) Thalamic nuclear localization for the confidence maps shown in panel **c**. Values are represented as the fraction of each thalamic nucleus covered by the average of confidence levels 1, 3, and 5 for thalamostriatal projections (magenta), the average of confidence levels 1, 4, and 7 for thalamocortical projections (cyan) and the average of confidence levels 1, 3, and 5 for thalamostriatal projections that lie within the white overlapping volume shown in panel **c**. The density of subregion-specific corticothalamic projections within each nucleus is shown in green. (**e**) The nuclear localization data, as described in panel **d**, are grouped by projection type (thalamocortical, thalamostriatal, overlap, and corticothalamic). As examples, only the thalamic targets of basal ganglia output (MD, Pf, VAL, and VM) are shown (see Figure 6—figure supplement 1 for full dataset). **DOI:** http://dx.doi.org/10.7554/eLife.19103.020

In addition to being a major input source to the striatum, the thalamus is also one of the primary output targets of the basal ganglia (Haber and Calzavara, 2009; Parent and Hazrati, 1995). Furthermore, the thalamus extensively interconnects with the cortex, thereby creating a cortico-thalamo-basal ganglia circuit loop (Figure 6a). To obtain a complete picture of the organization of this circuit, we overlaid the thalamic confidence map for thalamocortical projections to a given cortical subregion (Hunnicutt et al., 2014) with the thalamic confidence map for thalamostriatal projections that target the striatal field innervated by the same cortical subregion (Figure 4a). Figure 6c shows a representative example of this overlay process corresponding to the somatosensory cortices (S1/2) (see Figure 6—figure supplement 1 for all cortical subregions). When further aligning these confidence maps to the atlases, we observed that projection patterns varied across thalamic nuclei (Figure 6d). Of interest, VPM and VPL target S1/2 without projecting to the corresponding cortical projection field in the striatum (Figure 6c–d and Figure 6—figure supplement 1a–b, cyan); the intermediodorsal nucleus (IMD), mediodorsal nucleus (MD), rhomboid nucleus (RH), perireuniens nucleus (PR), submedius nucleus (SM), paraventricular nucleus (PVT), and CM send projections to the S1/2 projection field in the striatum without innervating S1/2 directly (Figure 6c–d and Figure 6—figure supplement 1a–b, magenta), whereas the posterior thalamic nucleus (Po), Pf, LP, paracentral nucleus (PCN), and centrolateral nucleus (CL) project to both targets (Figure 6c–d and Figure 6—figure supplement 1a–b, white).

**Figure 6—figure supplement 1.**
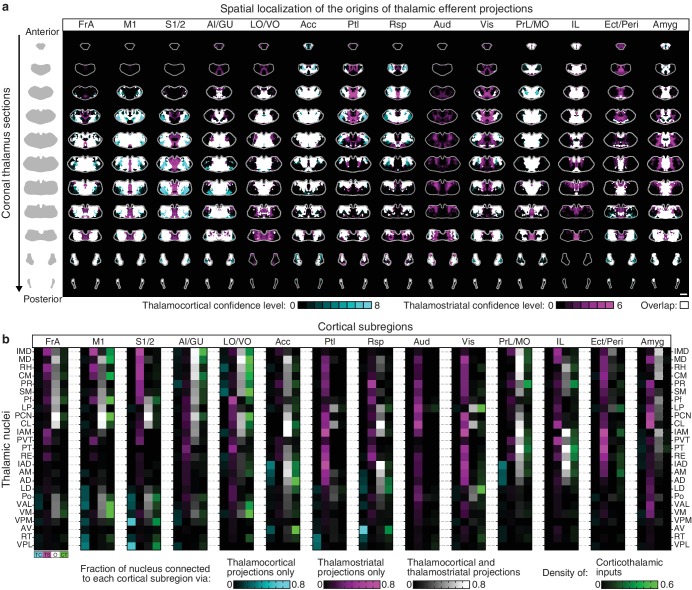
Organization of the thalamus in cortico-thalamo-basal ganglia loops. (**a**) Example coronal sections through the thalamus from anterior to posterior. Overlaid thalamocortical and thalamostriatal confidence maps as described in Figure 6b. Each column shows the origin of thalamic efferent projections associated with the 14 analyzed cortical subregions (section positions are the same as in Figure 4a). (**b**) Nuclear localization for the confidence maps shown in panel **a**. Values are represented as the fraction of each thalamic nucleus covered by the average of confidence levels 1, 3, and 5 for thalamostriatal projections (magenta), the average of confidence levels 1, 4, and 7 for thalamocortical projections (cyan) and the average of confidence levels 1, 3, and 5 for thalamostriatal projections that lie within the white overlapping volume shown in panel **a**. The density of subregion-specific corticostriatal projections within each nucleus is shown in green (See Materials and methods for details). **DOI:** http://dx.doi.org/10.7554/eLife.19103.021

**Figure 6—figure supplement 2.**
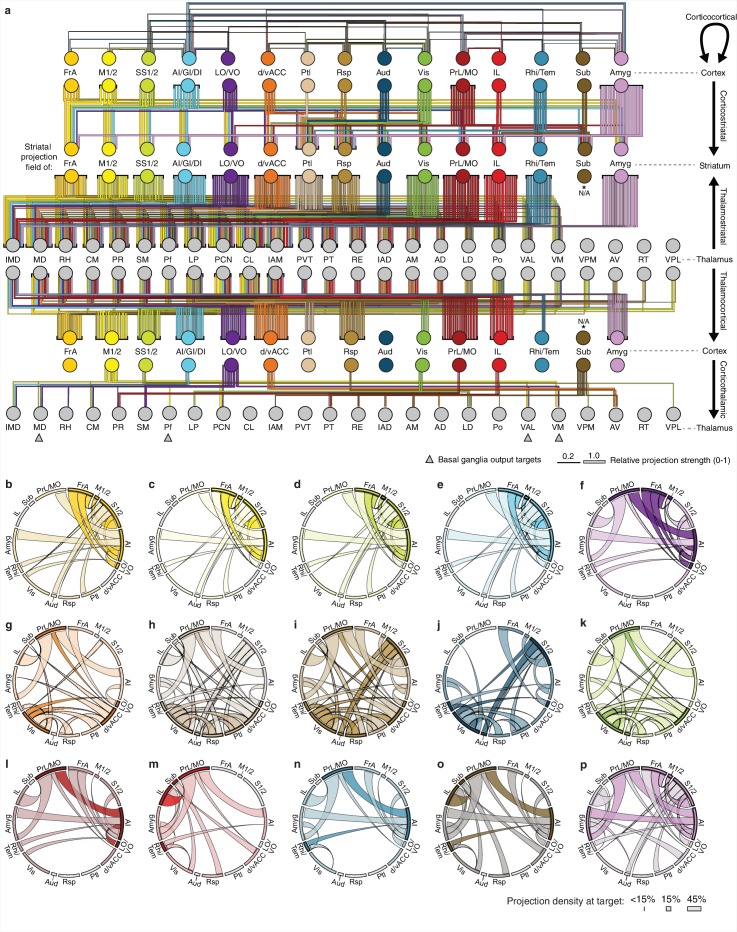
Overview of network interactions throughout the cortico-thalamo-basal ganglia circuit by subregion. (**a**) Network diagram of all corticocortical, corticostriatal, thalamostriatal, thalamocortical, and corticothalamic connections (top to bottom) associated with each cortical subregion. Corticocortical connections indicate a projection density >15% in the target area with the line color indicating the projection source. Corticostriatal projections are shown for primary convergent subregions, i.e. subregions whose projection fields converge with >50% the target projection field of each other cortical subregion (see panels **b–p** for details). Thalamostriatal projections (i.e. those that converge in the striatum with the projection field of each cortical subregion) are indicated for each thalamic nucleus with >20% of its volume contributing to the convergent projections (see Figure 4c–d). Thalamocortical projections are also shown for each thalamic nucleus with >20% of its volume contributing projections to the indicated cortical subregion (See Figure 6—figure supplement 1b, and [Hunnicutt et al., 2014]). Corticothalamic projections are indicated for projections where > 20% of the thalamic nucleus received projections from the corresponding cortical subregion (Figure 6—figure supplement 1b). Arrows indicate the thalamic targets of basal ganglia output. All line widths indicate either the relative density in target area (corticocortical and corticothalamic projections), fraction of nucleus covered (thalamocortical and thalamostriatal projections), or fraction convergent (corticostriatal) for all source-target characterizations, as described in Figure 6. (**b–p**) Chord diagrams highlighting the relationships between the cortical subregions that form the primary convergent inputs to the striatal projection fields of each other cortical subregion, as described for corticostriatal projections in panel **a**. Individual intra-cortical relationship maps shown for (**b**) FrA, (**c**) M1/2, (**d**) S1/2, (**e**) AI/GI/DI, (**f**) LO/VO, (**g**) d/vACC, (**h**) Ptl, (**i**) Rsp, (**j**) Aud, (**k**) Vis, (**l**) PrL/MO, (**m**) IL, (**n**) Rhi/Tem, (**o**) Sub, and (**p**) Amyg. The relative projection density at the target is indicated by the width of the arc at the target. Corticocortical connections are shown between two primary inputs (darker colored arcs) and for a primary input to or from a secondary cortical subregion that is not a primary input (lighter colored arcs). Corticocortical connections are indicated for projections with a density >15% in the target area. **DOI:** http://dx.doi.org/10.7554/eLife.19103.022

**Figure 6—figure supplement 3.**
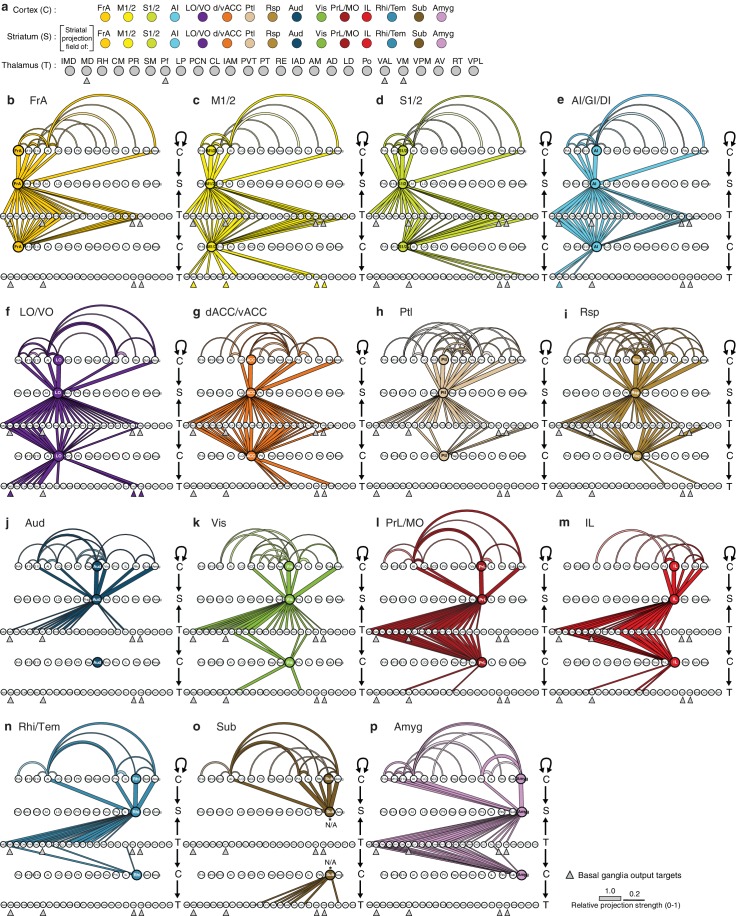
Network interactions throughout the cortico-thalamo-basal ganglia circuit by subregion, organized according to the cortical subregions. (**a**) Order of cortical (C), striatal (S), and thalamic (T) subregions depicted in panels **b–p**, with arrows below MD, Pf, VAL, and VM indicating the thalamic targets of basal ganglia output. (**b–o**) Network diagrams, as described in Figure 6—figure supplement 2, for individual subregions, indicating all corticocortical, corticostriatal, thalamostriatal, thalamocortical, and corticothalamic connections associated with each cortical subregion. Individual network interaction maps are shown for (**b**) FrA, (**c**) M1/2, (**d**) S1/2, (**e**) AI/GI/DI, (**f**) LO/VO, (**g**) d/vACC, (**h**) Ptl, (**i**) Rsp, (**j**) Aud, (**k**) Vis, (**l**) Rhi/Tem, (**m**) Amyg, (**n**) IL, (**o**) Sub, and (**p**) PrL/MO. Letters to the right of each panel indicate whether each row is corresponds to C, S, or T, and arrows indicate the direction of each projection. Corticocortical connections (projection density >15%) are shown for projections from all cortical subregions forming primary convergent inputs with the indicated cortical subregion. Corticostriatal projections are shown for the cortical subregions that form primary convergent projections with the projection field of the indicated cortical subregion. Thalamostriatal projections that converge in the striatum with the projection field of the indicated cortical subregion are shown for each thalamic nucleus having >20% of its volume contributing to the convergent projections (see Figure 4c–d). Thalamocortical projections are shown for each thalamic nucleus with >20% of its volume contributing to projections to the indicated cortical subregion (See Figure 6—figure supplement 1b, and [Hunnicutt et al., 2014]). Corticothalamic projections are shown if >20% of the thalamic nucleus received projections from the corresponding cortical subregion. Since Amyg constituted a primary convergent input to all corticostriatal projection fields, projections from Amyg were not included in corticocortical maps individually. However, the primary input to the striatal projection field is still indicated. **DOI:** http://dx.doi.org/10.7554/eLife.19103.023

Cortical feedback to the thalamus is an important component of the cortico-thalamo-basal loop (Figure 6a). To include corticothalamic connections, preprocessed data describing the density of projections in the thalamus for each cortical injection examined herein were downloaded from the AIBS application programming interface (API) (http://connectivity.brain-map.org/, see Materials and methods). These corticothalamic data were integrated into our analysis (Figure 6d–e, Figure 6—figure supplement 1a–b, green), providing a crucial feedback pathway necessary to fully understand excitatory connectivity within the cortico-thalamo-basal ganglia circuit.

Corticocortical connections provide another possible path for information integration within this circuit. It has been proposed that cortical subregions whose projections converge in the striatum are more strongly interconnected than subregions that do not converge and non-converging cortical subregions are less interconnected (Yeterian and Van Hoesen, 1978), which may maintain information segregation. To test the hypothesis that cortical subregions that converge in the striatum are more strongly cortically connected, the same preprocessed AIBS API datasets that were used to map the corticothalamic projections were also used to determine the density of projections between each cortical subregion. Cortical subregions whose projections converge >20% within the striatal projection fields of each other are shown in Figure 6—figure supplement 2b–p. The primary convergent subregions are indicated with a darker color, and projections are shown as ribbons between subregions, where dark ribbons indicate connections between two primary convergent subregions (Figure 6—figure supplement 2b–p). As shown, primary convergent inputs with most cortical subregions form distributed cortical networks, for example frontal subregions IL and d/vACC are more interconnected with cortical subregions that they do not converge with in the striatum. However, some areas, such as FrA, M1/2, S1/2, and AI form highly recurrent networks with convergent subregions (Figure 6—figure supplement 2b–p). These varied connectivity patterns suggest that different pathways through the cortico-thalamo-basal ganglia circuit may have different levels of information integration, supporting the existence of both open- and closed-loop circuits.

To complete the investigation of the cortico-thalamo-basal ganglia circuit, the thalamocortical, thalamostriatal, corticothalamic, and corticocortical data were compared for MD, ventral anterior-lateral complex (VAL), ventromedial nucleus (VM), and Pf (Figure 6e, Figure 6—figure supplement 2a and Figure 6—figure supplement 3), which are the main thalamic targets of the basal ganglia output (Deniau and Chevalier, 1992; McFarland and Haber, 2002; Smith et al., 2014). A full circuit map shows the relative levels of input convergence between cortical and thalamic subregions, as well as with the basal ganglia output targets (Figure 6—figure supplement 2a), and by focusing on connectivity related to specific subregions, information flow can be traced through the circuit (Figure 6e and Figure 6—figure supplement 3). For example, these comparisons reveal that the motor cortex (M1/2) directly innervates, receives projections from, and converges in the striatum with all of thalamic nuclei that receive basal ganglia output. This extensive interconnectivity of the thalamic nuclei innervated by the basal ganglia with motor-related cortical and striatal subregions, particularly M1/2 and FrA (Figure 6e and Figure 6—figure supplement 3), suggests the importance of cortical motor information for basal ganglia function. In contrast, the orbital cortices (LO/VO) are highly interconnected with MD, VM, and, to a lesser extent, with VAL, but there are no direct corticothalamic or thalamocortical interactions between LO/VO and Pf. Thus, although LO/VO plays an important role in this circuit, it does not display the ubiquitous connectivity pattern seen with M1/2 (Figure 6e and Figure 6—figure supplement 3). Similarly, whereas S1/2 is interconnected with VM, Pf, and VAL, it does not send or receive MD projections directly, even though both S1/2 and MD send converging axons in the striatum (Figure 6e and Figure 6—figure supplement 3). Together, these data provide a comprehensive picture of information flow through the cortico-thalamo-basal ganglia circuit.

### The cortico-thalamo-basal ganglia loop organization for clustered striatal subdivisions

Taking advantage of the extensive cortico-thalamo-basal ganglia circuit data described above, we examined whether the information flow is segregated with respect to the four major striatal subdivisions described in Figure 5. First, the primary cortical inputs to each striatal subdivision were identified as either (1) cortical subregions whose dense projection fields occupy >20% of voxels in the striatal subdivision (Figure 7—figure supplement 1a–b), or (2) cortical subregions with >50% of their dense projections within the striatal subdivision (Figure 7—figure supplement 1c). The amygdala was excluded from this analysis because it met the criteria for primary inputs for all striatal subdivisions. The primary inputs identified for each subdivision were: dorsomedial (red): d/vACC, Ptl, Rsp, Vis, PrL/MO, and LO/VO; posterior (green): Aud, Vis, and Rhi/Tem; dorsolateral (cyan): FrA, M1/2, S1/2, and AI; and ventral (dark blue): PrL, Sub, IL, Rhi/Tem, and AI (Figure 7—figure supplement 1d).

**Figure 7.**
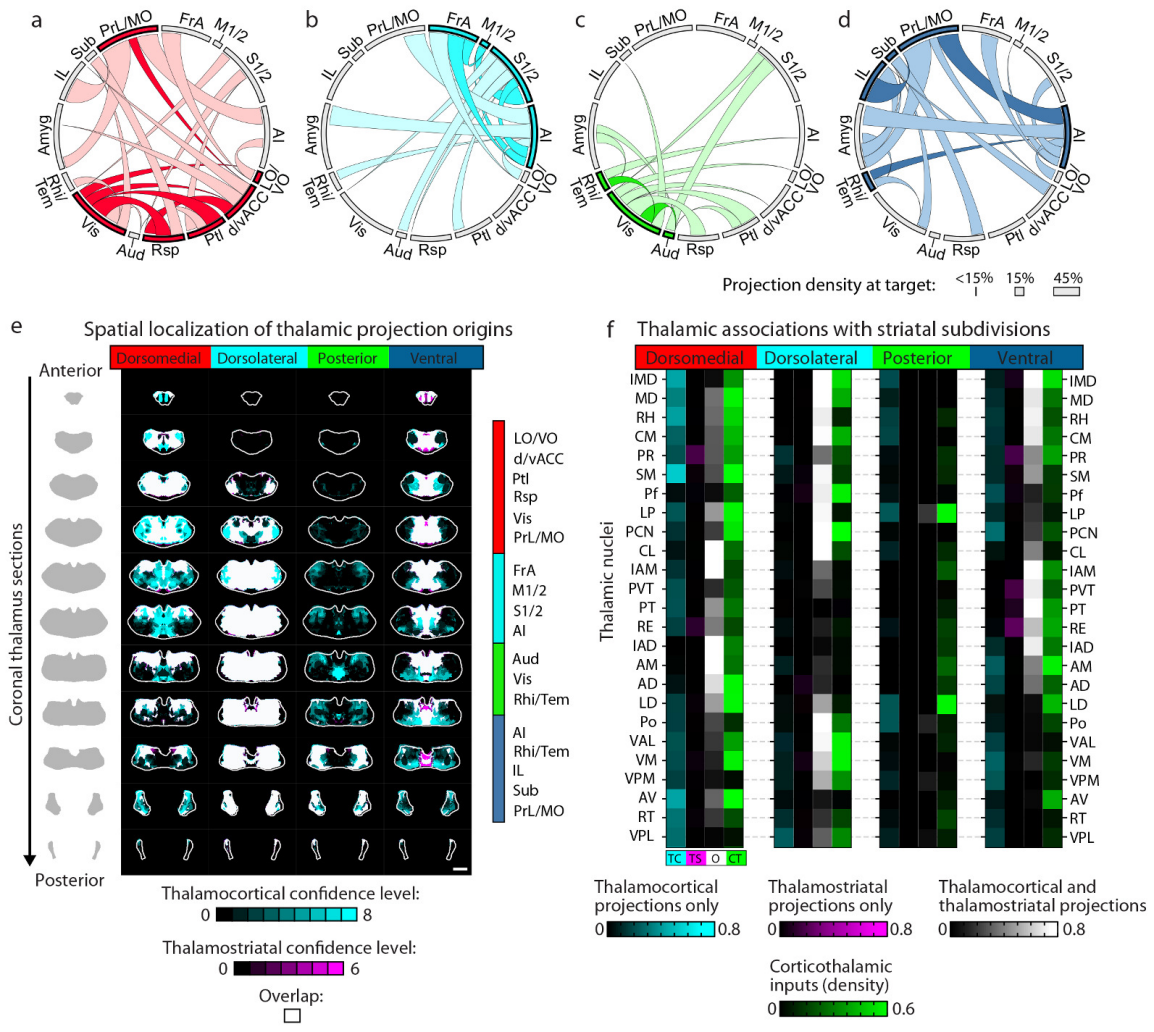
Cortico-thalamo-basal ganglia circuit organization for striatal subdivisions. (**a–d**) Chord diagrams highlighting the relationships between the cortical subregions forming the primary inputs to the (**a**) dorsomedial, (**b**) dorsolateral, (**c**) posterior, and (**d**) ventral striatal subdivisions respectively. The projection density at the target subregion is indicated by the width of the arc at the target. Corticocortical connections are shown for the afferent and efferent projections of subregions that form the primary input to each striatal subdivision. Primary input regions are shown in darker colors. Darker colored ribbons indicate connections between two primary input subregions, and lighter colored ribbons indicate the connections of a primary input subregion with secondary cortical subregions that do not project to the corresponding striatal subdivision. Connections are shown for projections with a density >15% in the target area. (**e**) Example coronal sections through the thalamus from anterior to posterior with overlaid thalamocortical and thalamostriatal confidence maps, as described in Figure 5. Each column shows the origin of thalamic projections associated with the four striatal subdivisions shown in Figure 5. Thalamocortical and corticothalamic projections are grouped across the cortical subregions that form the primary inputs of each striatal subdivision, as determined in Figure 7—figure supplement 1 (section positions are the same as in Figure 4a). (**f**) Nuclear localization for the convergence confidence maps shown in panel **e**. Values are represented as the fraction of each thalamic nucleus covered by the average of confidence levels 1, 3, and 5 for thalamostriatal projections (magenta), the average of confidence levels 1, 4, and 7 for thalamocortical projections (cyan) and the average of confidence levels 1, 3, and 5 for thalamostriatal projections that lie within the white overlapping volume shown in panel **e**. The density of subregion-specific corticostriatal projections within each nucleus is shown in green (See Materials and methods for details). TC: thalamocortical confidence maps; TS: thalamostriatal confidence maps; O: overlay of thalamocortical and thalamostriatal confidence maps; CT: corticothalamic projections. **DOI:** http://dx.doi.org/10.7554/eLife.19103.024

The above information allows us to further investigate the cortical and thalamic connections with respect to each striatal subdivision (Figure 7—figure supplement 1). The thalamocortical, thalamostriatal, corticothalamic, and corticocortical data were compared for each striatal subdivision (Figure 7) using an approach analogous to that used to evaluate information flow through the cortico-thalamo-basal ganglia circuit related to individual cortical subregions (Figure 6 and Figure 6—figure supplement 2). As seen with the cortical subregion-based analysis (Figure 6—figure supplement 2), the number and strength of corticocortical connections varied across networks (Figure 7a–d). Cortical areas associated with the dorsolateral striatal subdivision are the most recurrently connected having each primary dorsolateral input connected to at least two other primary dorsolateral inputs (Figure 7b). Interestingly, nearly all cortical subregions (except Sub) are connected to at least one other primary striatal subdivision input in their respective networks (Figure 7a–d).

**Figure 7—figure supplement 1.**
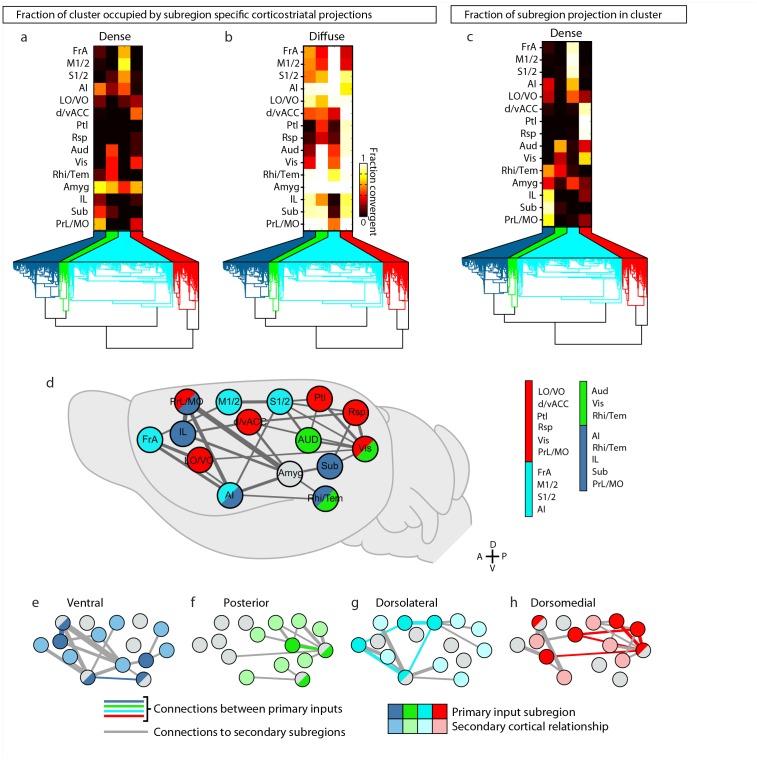
Intracortical interactions across cortical subregions that innervate the four striatal subdivisions. (**a–b**) Coverage plots indicating the fraction of each striatal cluster (columns) occupied by either diffuse or dense corticostriatal projections from each cortical subregion (rows), shown here with the striatal voxel dendrogram (bottom) for the four cluster threshold, as determined in Figure 5. (**c**) Coverage plot indicating the fraction of each subregion specific corticostriatal projection (rows) in each of the striatal clusters for the four cluster threshold (columns), with the striatal voxel dendrogram (bottom). (**d–h**) Schematic network diagrams indicating the intra-cortical relationships for the cortical subregions that make up the primary inputs to each striatal subdivisions defined by the four cluster threshold. (**d**) Overview schematic showing the relative locations of cortical subregions on a collapsed sagittal view of the mouse brain (gray). Primary inputs were as follows: ventral subdivision (dark blue): PrL, Sub, IL, Rhi/Tem, and AI; posterior subdivision (green): Aud, Vis, and Rhi/Tem; dorsolateral subdivision (cyan): FrA, M1/2, S1/2, and AI; dorsomedial subdivision (red): d/vACC, Ptl, Rsp, Vis, PrL/MO, and LO/VO. Cortical subregions with that target multiple striatal targets are indicated with multiple colors. Amyg projections met the criteria for primary inputs to all striatal subdivisions and is thus shown in gray. Lines connecting subregions indicate projections having a density >15% in the target area. (**e–h**) Schematic corticocortical network diagrams highlighting the spatial relationships between the cortical subregions forming the primary inputs to the (**g**) ventral, (**h**) posterior, (**i**) dorsolateral, and (**j**) dorsomedial subdivision, respectively. Intra-cortical connections are shown for the afferent and efferent projections of subregions that form the primary input to each in striatal subdivision. Connections between two primary inputs are indicated with a colored line, and connections between a primary input subregions and other cortical subregion are indicated with gray lines. **DOI:** http://dx.doi.org/10.7554/eLife.19103.025

Next, the thalamic relationships with the striatal subdivisions in the cortico-thalamo-basal ganglia circuit were examined (Figure 7e–f). The thalamic origins of projections to each striatal subdivision and the thalamocortical projections to the cortical subregions associated with the same striatal subdivision largely overlap. Thus, nearly all thalamic nuclei that target a given striatal subdivision also send projections to at least one of the cortical subregions that forms a primary input to that striatal subdivision (Figure 7e–f, white). This suggests a strong relationship between the thalamus and the cortex for subdivision-specific input integration in the striatum. In contrast, the projection field of corticothalamic feedback within each network at the thalamus only partially overlaps with the thalamocortical or thalamostriatal projecting nuclei (Figure 7f, cf. green and white/cyan). These data provide further evidence that the striatal clusters identified in the present study (Figure 5) represent functionally relevant striatal subdivisions, and give evidence for robust integration of cortical and thalamic information within each subdivision-associated cortico-thalamo-basal ganglia circuit.

### Anatomical inputs to the striatum are functional

**Figure 8.**
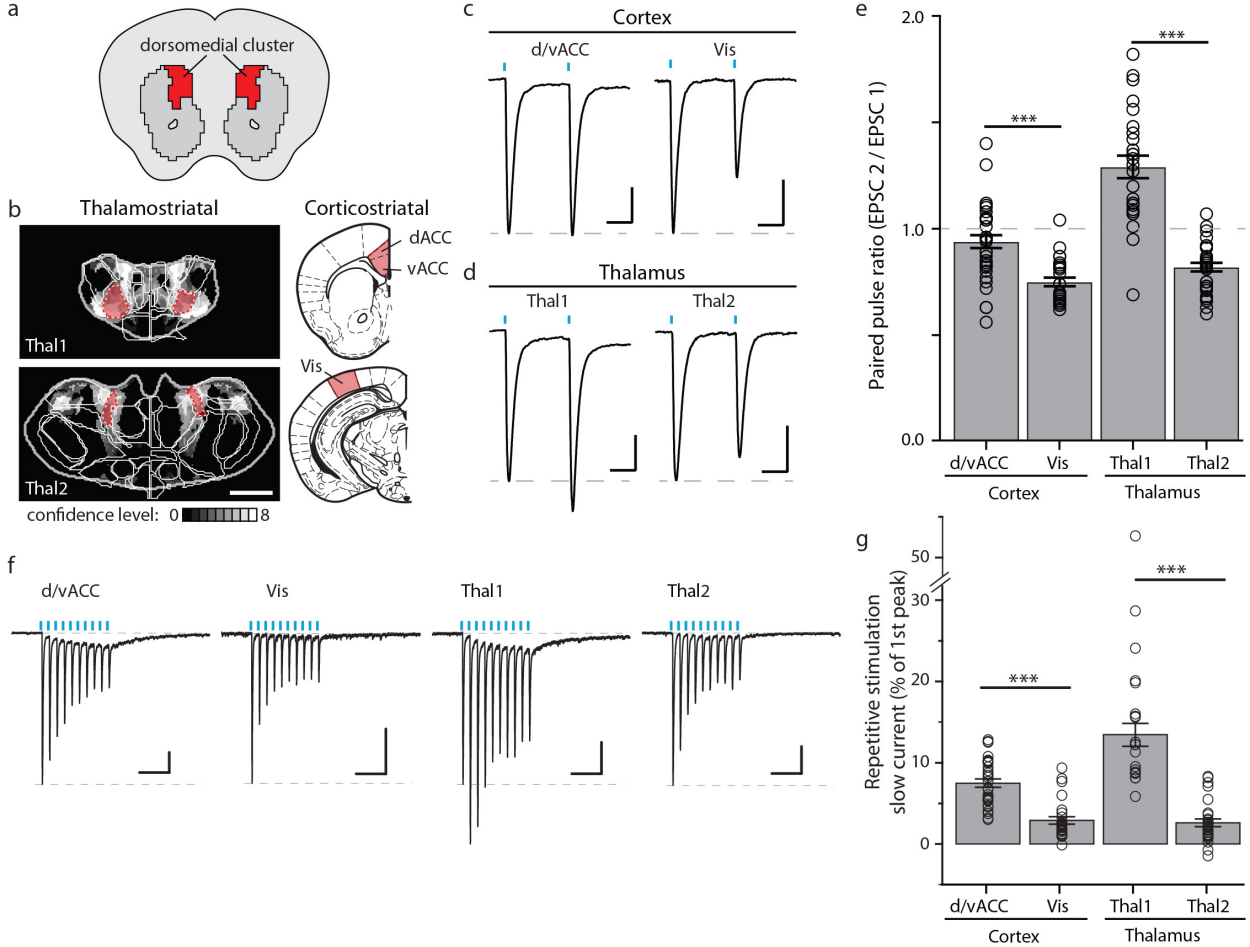
Optogenetic stimulation of cortico- and thalamo-striatal inputs converging on the DM striatal subdivision reveals functional heterogeneity. (**a**) Schematic representation of the DM striatal subdivision shown in red, as presented in Figure 5d. (**b**) The DM subdivision was identified by the convergence of thalamostriatal inputs originating from thalamic centers 1 (Thal1) and 2 (Thal2) (two left panels, respectively), based on the thalamostriatal confidence maps with the thalamic nucleus (white lines) fully encompassed by each center, shaded red (gray scale shows the confidence level as determined in Figure 4), and corticostriatal inputs originating from the d/vACC and Vis (shaded red, two right panels, PMBA). The red areas indicate the targets for viral injections. (**c**) Example traces of paired-pulse EPSCs recorded in MSNs within the DM striatal subdivision, elicited by photostimulation of specific corticostriatal inputs. (**d**) Example traces of paired-pulse EPSCs recorded in MSNs within the DM striatal subdivision, elicited by photostimulation of specific thalamostriatal inputs. (**e**) Quantification of paired-pulse ratio (PPR) evoked by photostimulation of specific cortico- and thalamo-striatal inputs reveals strong differences in PPR (*n_(d/vACC)_* = 34, *n_(Vis)_* = 26, *n_(Thal1)_* = 25, *n_(Thal2)_* = 32 cells, Kruskal-Wallis test, H = 60.8699, df = 3, p<0.0001; *post-hoc* Dunn’s test, Bonferonni-corrected p=0.0002) between distinct thalamic nuclei and distinct cortical subregions (*post-hoc* Dunn’s test, Bonferroni-corrected, ***p<0.001). (**f**) Example traces of repetitive photostimulation (20 Hz, 10 stimuli represented by blue lines) of the four cortico- and thalamo-striatal afferents. (**h**) Quantification of the slow current during repetitive photostimulation, relative to EPSC peak evoked by the first stimulus (***p<0.0001). Thal1, thalamic center 1; Thal2, thalamic center 2. Group data are presented as mean ± SEM. **DOI:** http://dx.doi.org/10.7554/eLife.19103.026

The striatal subdivisions described here were defined by their excitatory input patterns, leading us to investigate the functional differences between individual cortical and thalamic inputs to the striatum. Guided by our comprehensive striatal input maps, we examined functional properties of inputs to the dorsomedial (DM) striatal subdivision (Figure 8a and Materials and methods). The DM striatal subdivision receives robust innervation from two distinct thalamic areas, with the first area (Thal1) primarily encompassing the anteromedial thalamic nucleus (AM), and the second area (Thal2) including mainly the CL, the lateral portion of MD, and a portion of Po (Figure 5e–f, Figure 8a and Figure 8—figure supplement 1a). In addition, although the DM striatal subdivision receives input from many cortical subregions, dense innervations in this area originate primarily from the d/vACC and Vis (Figure 5b–c, Figure 8a, and Figure 8—figure supplement 1a, and also see [Berendse et al., 1992; Khibnik et al., 2014; McGeorge and Faull, 1989]). We performed localized injections of recombinant adeno-associated virus (AAV) (serotype 2) expressing channelrhodopsin (CsChR-GFP) (Klapoetke et al., 2014) individually into the four cortical and thalamic subregions (d/vACC, Vis, Thal1 and Thal2), and confirmed the presence of projections in the DM striatal subdivision (Figure 8—figure supplement 1a–b). Photostimulation of the CsChR-positive axons in the DM striatal subdivision triggered excitatory postsynaptic currents (EPSCs) recorded from medium spiny neurons (MSNs), confirming functional connectivity between each input source and the DM striatal subdivision (Figure 8c–d and Figure 8—figure supplement 1b).

**Figure 8—figure supplement 1.**
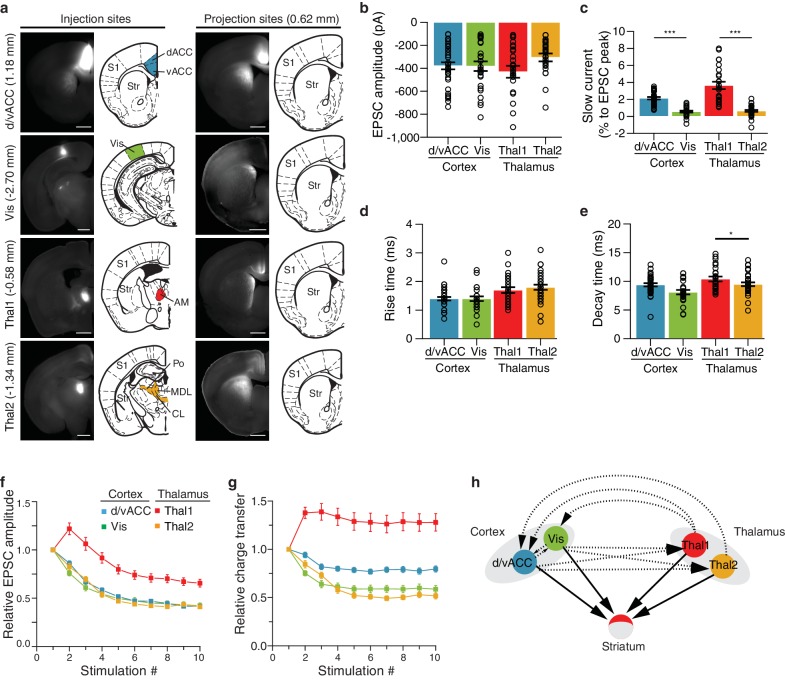
Characterization of functional differences between and within individual cortico- and thalamostriatal inputs to the dorsomedial (DM) striatum. (**a**) Example injection sites (left column) in dorsal and ventral anterior cingulate cortex (d/vACC), visual cortex (Vis), thalamic center 1 (Thal1), and thalamic center 2 (Thal2); example projection sites (right column) within the DM striatum, shown with corresponding sections from Paxinos Mouse Brain Atlas (PMBA, [Paxinos and Franklin, 2001]). Injection of channelrhodopsin CsChR2-GFP of Thal1 infected the anteromedial thalamic nucleus (AM), whereas injection of Thal2 mainly infected centrolateral thalamic nucleus (CL) together with a portion of the posterior thalamic nucleus (Po), and the lateral part of the mediodorsal thalamic nucleus (MDL). The value in parentheses represents the distance from bregma, with positive value anterior to bregma. S1, primary somatosensory cortex; Str, striatum. Scale bars represent 1 mm. (**b–e**) Quantification of excitatory postsynaptic current (EPSC) amplitude (**b**, p>0.05), slow current relative to EPSC peak (**c**, p<0.0001), rise time (**d**, p<0.05), and decay time (**e**, p<0.0001). (**f**) Quantification of relative EPSC amplitude over 10 consecutive stimuli, normalized to EPSC amplitude evoked by the first stimulus (p<0.0001, main effect of injection site: Thal1 vs. Thal2, p<0.0001; d/vACC vs. Vis, p>0.05). (**g**) Quantification of the EPSC charge transfer evoked by each stimulus, normalized to the charge transfer evoked by the first stimulus (p<0.0001, main effect of injection site: Thal1 vs. Thal2, p<0.001; vACC vs. Vis, p<0.01). (**h**) Schematic representation of the two cortical subregions and two thalamic centers converging to the DM (arrows with solid lines) with suggested region specific connectivity based on previous work (arrows with dotted lines). Statistical comparisons between cortical or thalamic afferents are marked *p<0.05, ***p<0.001. **DOI:** http://dx.doi.org/10.7554/eLife.19103.027

### Synaptic heterogeneity originating from individual cortical or thalamic inputs

Recent studies have identified functional differences between corticostriatal and thalamostriatal inputs with respect to their synaptic properties (Ding et al., 2008; Ellender et al., 2013; Smeal et al., 2007). However, the precise synaptic properties of corticostriatal and thalamostriatal inputs differed qualitatively across studies. To determine if these discrepancies were due to a lack of subregion specificity when stimulating cortical or thalamic inputs (Kreitzer and Malenka, 2008), we examined the synaptic properties of Thal1, Thal2, d/vACC, and Vis inputs to MSNs in the DM striatal subdivision.

By using a paired-pulse ratio (PPR) experiment to examine the presynaptic release probability, we found that paired-pulse photostimulation of Thal1 axons resulted in facilitation of synaptic transmission onto MSNs, whereas Thal2 axons showed synaptic depression (Figure 8d–e). Consistently, repetitive photostimulation (10 stimuli, 20 Hz) of the two thalamic inputs resulted in sustained Thal1 EPSCs with larger relative magnitude than those evoked by Thal2 axons (Figure 8f–g and Figure 8—figure supplement 1f–g). Moreover, a sustained slow current, which is evident even in singly evoked EPSCs (Figure 8—figure supplement 1c) in Thal1 inputs, but not in Thal2 inputs, contributed to an overall increased charge transfer during the consecutive photostimuli (Figure 8—figure supplement 1g). Similarly, different cortical projections to the DM striatal subdivision also exhibited heterogeneity (Figure 8c–g and Figure 8—figure supplement 1b–h). The PPR of Vis inputs exhibited strong synaptic depression, which was not observed in d/vACC inputs (Figure 8c and e). Repetitive photostimulation of Vis or d/vACC inputs resulted in similar levels of synaptic depression (Figure 8f, and Figure 8—figure supplement 1f). However, repetitive stimulation d/vACC, but not Vis inputs, resulted in a prominent slow sustained current that led to increased charge transfer at d/vACC–DM synapses relative to Vis–DM synapses over consecutive photostimuli (Figure 8g and Figure 8—figure supplement 1g). Thus, the discrepancies observed across previous studies may be due to electrical stimulation of cortical or thalamic inputs to the striatum lacking sufficient subregion specificity. These data provide, to our knowledge, the first examples of intra-thalamic and intra-cortical heterogeneity among striatal excitatory inputs, suggesting subregion-dependent integration in the striatum.

## Discussion

To our knowledge, the present study provides the first comprehensive excitatory input map of the mouse striatum. Given the broad roles of the striatum in action selection, motor execution, and reward, understanding how individual inputs precisely project to the striatum and how such inputs may interact with one another is a step forward in dissecting the circuit mechanisms underlying striatal function.

An unbiased cluster analysis of the corticostriatal input patterns reveals that the striatum can be divided into four large subdivisions with clear boundaries (Figure 5). Three of these subdivisions most likely correspond to the traditional dorsomedial, dorsolateral, and ventral domains thought to play critical roles in goal-directed behaviors, habitual behaviors, and affective control of behaviors, respectively. The fourth subdivision at the posterior end of the striatum may represent a previously unappreciated functional domain, and illustrates the existence of heterogeneity along the A-P axis. Recent evidence has suggested that the posterior part of the striatum receives inputs from anatomically distinct populations of dopamine neurons (Menegas et al., 2015), bears a unique MSN subpopulation composition (Gangarossa et al., 2013), and has been shown in primates to mediate specific behavioral functions (Yamamoto et al., 2013). Our data identify and describe the distinct connectivity of the posterior striatum in mice, showing that this posterior subdivision receives strong inputs from the auditory, visual, and rhinal cortices, as well as from the amygdala, suggesting that this area may process multi-modality sensory inputs in the context of emotional information (Figure 5b). We also found that the associative striatum, consistent with its proposed function, receives extremely heterogeneous inputs (Figure 5d). The comprehensive input map presented here may also guide future experiments aimed at understanding the function of individual cortical, thalamic, or striatal subdivisions by allowing for a systematic evaluation of all locations to perform imaging and recording experiments.

An orthogonal approach to spatially subdividing the striatum involves the separation of the patch and the matrix compartments via neurochemical markers (Gerfen, 1992; Graybiel and Ragsdale, 1978). These subdivisions have been shown, mainly in primates, to receive distinct patterns of cortical inputs (Gerfen, 1992). In the future, it will be interesting to examine how the patch/matrix subdivisions interact with the subdivisions described herein to orchestrate striatal function. Besides geometric subdivisions, brain circuitry is also organized based on different neuronal cell types. Future studies combining cell-type-specific and subregion-specific circuit analyses to examine how subregion-specific inputs differentially innervate different cell types, for example, the D1 and D2 MSNs, in striatal subdivisions will provide additional insights into the striatal circuitry in normal and diseased brains.

The work presented here was achieved by integrating two large-scale viral-tracing datasets and vigorous data analyses. Recent technical advances have made it possible to systematically generate whole brain projection data at mesoscopic resolution (Hunnicutt et al., 2014; Oh et al., 2014; Pinskiy et al., 2015; Zingg et al., 2014). However, it remains challenging to integrate such large datasets (typically >50 terabytes) obtained from different research teams under different conditions, and with various forms of metadata. To our knowledge, our study represents the first example of combining two different large mesoscopic imaging datasets (Figure 1). Our efforts were fruitful for several reasons. First, similar viral infection reagents were used, which standardized many properties of the imaging data, including comparable injection sites and high-imaging sensitivity. Second, our analyses utilize the different advantages of each dataset. For the thalamic dataset (Hunnicutt et al., 2014), because the thalamic nuclei can be smaller than the size of an individual injection, high-density, overlapping injections are necessary to achieve adequate mapping resolution (Hunnicutt et al., 2014). In contrast, the injections in the AIBS Mouse Connectivity Atlas dataset are sparse and mostly non-overlapping, but they are spread across many brain regions (Kuan et al., 2015; Oh et al., 2014), making them suited for mapping projections from cortical areas, which are larger, more widely spread, and better demarcated than the mouse thalamic nuclei.

Although most current efforts at mesoscopic circuit mapping focus on illustrating the connections between two macroscopic brain regions (Mitra, 2014), information processing in the brain often involves several brain regions. We were able to expand our systematic circuit analyses to include three main brain regions that form a complete loop. Specifically, we examined how subregion-specific projections from the thalamus and the cortex converge in the striatum, and how the thalamus is interconnected with the cortex and basal ganglia (Figure 2 and Figure 6). To do this, we carried out several analyses. First, we mapped the thalamic origins of thalamostriatal projections and identified the converging subregion-specific corticostriatal inputs (Figure 2 and Figure 5—figure supplement 1). Second, we illustrated the relationships between the thalamic subregions that directly project to a cortical subregion and the thalamic subregions that converge with the same cortical subregion in the striatum (Figure 6 and Figure 6—figure supplement 1). It is worth noting that the current thalamic dataset does not include the medial and lateral geniculate nuclei (MGN and LGN, respectively) (Hunnicutt et al., 2014), although reports in rat (LeDoux et al., 1984; Veening et al., 1980), as well as our visual inspection of AIBS thalamic injections (data not shown), suggest that the MGN, but not the LGN, projects to the posterior striatum. Third, since the thalamus is the major target of basal ganglia output, and only specific thalamic subregions receive basal ganglia innervation (Deniau and Chevalier, 1992; Gerfen and Bolam, 2010; McFarland and Haber, 2002; Smith et al., 2014), we examined how these basal ganglia-innervated thalamic subregions differ in connectivity patterns as compared to other thalamic subregions (Figure 6). We found that these thalamic subregions have strong ties with motor cortical subregions, and converge in the same striatal subdivisions with corticostriatal projections from those motor cortical subregions, consistent with the notion that the basal ganglia play a critical role in movement controls and are in close coordination with the cortical motor processing.

Regarding the corticostriatal inputs, the results of the present study are largely consistent with related literature in rat and primate, although several sources could potentially contribute to any discrepancy in isolated cases. First, the relative small size of mouse brain allows the systematic tracing coverage of all cortical subregions and >93% volume of the thalamus with individual injections of small (500–600 µm) sizes, and the imaging of the entire projections of every injection. The level of completeness has not been previously achieved in any mammalian species. The comprehensiveness of the datasets allows us to perform quantitative analyses that are difficult with a few example images. On the other hand, because of the relative small size of mouse brain, and the lack of anatomical landmarks for demarcating certain subregions, accurately assigning cortical subregions can be challenging (e.g., for M1 and M2, see [Mao et al., 2011]). For cortical injections, we applied stringent criteria (see Materials and methods) in the selection process and, as a result, only <10% of AIBS injections was included. Even with great care, the lack of clear landmarks for certain mouse cortical subregion definition may still be a source of variability. Second, it is important to note that there are two distinct projection patterns of corticostriatal axons, a localized dense core projection and a diffuse projection that generally spans a wider area than the dense projections (Mailly et al., 2013). Many previous mapping studies preferentially focused on the dense projections, particularly when reporting a summary result of several tracing experiments. In our data, we mapped both the dense and diffuse projections, and this revealed some previously underappreciated convergence patterns, such as the diffuse somatosensory-motor inputs to a portion of the limbic striatum (mid-dark blue, Figures 5, 15 clusters) (Draganski et al., 2008), and the widespread diffuse projections of LO/VO to nearly the entire striatal volume (Figure 2). Our dense projection results are highly consistent with the corticostriatal projection distributions reported in the literature (Gruber and McDonald, 2012), and studies that separate the dense and diffuse projections describe similarly widespread diffuse projections (Haber et al., 2006; Mahan and Ressler, 2012). Finally, there might be circuit differences at mesoscopic resolution across species due to parallel evolution and it will be interesting to systematically compare them in the future when similar type of data become available in other mammalian species.

Recent work from Hintiryan and colleagues uses an anterograde tracing dataset from cortical injections to illustrate the corticostriatal circuits and demonstrate the usefulness of large scale mesoscopic projection mapping to study ‘circuitry-specific connectopathies’ (Hintiryan et al., 2016). Although Hintiryan et al. and our studies both use comprehensive mesoscopic cortical projections in the striatum to understand striatal circuit logic, these two studies are also complementary. In addition to cortico-dorsal striatal projections, our study also includes cortico-ventral striatal projections, thalamostriatal projections, as well as corticocortical and thalamocortical connectivity. Our thalamostriatal dataset is of particular interest because thalamostriatal data for mouse is scarce in the literature and the circuits are much less understood compared to the corticostriatal pathways. The completeness of our dataset allows us to illustrate the features of the cortico-thalamo-basal ganglia loop (Figure 6, Figure 7, Figure 6—figure supplement 2, Figure 6—figure supplement 3, and Figure 7—figure supplement 1).

The anatomical axonal projection map suggests, but does not guarantee, synaptic connections (e.g., see [Dantzker and Callaway, 2000; Mao et al., 2011; Shepherd and Svoboda, 2005]), especially in the striatum where many fasciculated axons pass through without forming synapses. Therefore, we examined the existence of synaptic connections using optogenetic stimulation and physiological recording for the anatomically described corticostriatal and thalamostriatal projections (Figure 8 and Figure 8—figure supplement 1). Our results indicate that, for the projections identified after computer-assisted exclusion of passing fasciculated axons, the mapped axonal projections do form functional synapses in the striatum (Figure 8 and data not shown). Furthermore, taking advantage of our comprehensive anatomical input map, we examined functional heterogeneity of synaptic connections in the striatum. A series of recent studies have shown that cortical and thalamic inputs form functionally unique synapses in the striatum, although their synaptic properties remain controversial. In addition, little was known about whether different subregions within the cortex or the thalamus form functionally unique synapses in the striatum. We found that distinct cortical and thalamic subregions each give rise to synapses in the striatum with unique synaptic properties (Figure 8), providing a potential explanation for the discrepancies reported previously (Ding et al., 2008; Smeal et al., 2007) when the thalamic or cortical inputs were stimulated in a non-subregion-specific manner. Taken together, these results presented here demonstrate the value of creating comprehensive input maps, and their utility in guiding the effective design of functional studies.

## Materials and methods

All animal experiments were conducted according to National Institutes of Health guidelines for animal research and were approved by the Institutional Animal Care and Use Committee (IACUC protocol number: IS00003542). All mice were housed in a vivarium with 12 hr light/dark cycle (lights on at six am). All calculations were performed in MATLAB (MathWorks). Related custom software is available at Github: https://github.com/BJHunnicutt/anatomy.

### Thalamostriatal projectome data overview

Thalamic injection and imaging data were generated as described previously (Hunnicutt et al., 2014). In brief, viral injections were performed in male and female wild-type C57BL/6J mice at postnatal days 14–18 using a hydraulic apparatus to stereotaxically inject ~10 nl of rAAV (serotype 2/1) encoding either eGFP or tdTomato. Two weeks post-injection, each brain was fixed, cryostat-sectioned at 50 µm, and imaged using a Hamamatsu Nanozoomer imaging system (Japan), resulting in 0.5 µm/pixel lateral resolution for the full-brain fluorescence images of all injections and their cortical and striatal projections. Injection sites were then re-imaged at a lower exposure time on either the Nanozoomer or a Zeiss Axio Imager to avoid overexposure. Injection site images were matched to their corresponding full brain Nanozoomer images through rigid translation and rotation using manually selected anatomical landmarks visible in both images. The thalamus was manually segmented from the full brain images, and injection sites were segmented from background fluorescence in the green and red channels using a supervised custom MATLAB routine. The alignment of injection sites and thalami, and the generation of the model thalamus were described previously (Hunnicutt et al., 2014). Each injection and image was manually inspected for quality control.

### Corticostriatal projectome data overview

The raw data for cortical viral injection and projection were obtained from the AIBS Mouse Connectivity Atlas (http://connectivity.brain-map.org/) (Research Resource Identifier (RRID): SCR_008848) (Oh et al., 2014). The data generation pipeline was analogous to that used in the thalamostriatal projectome dataset, with a few differences. Briefly, a single iontophoretic injection of AAV2/1 encoding eGFP was performed per animal at postnatal day 56 (Oh et al., 2014). Both male and female wild-type and Cre-expressing C57BL/6J mice were used. At two weeks post-infection, the animals were fixed and imaged using a TissueCyte 1000 serial two-photon tomography system, with a lateral resolution of 0.35 µm/pixel and a z-resolution of 100 µm. The AIBS Mouse Connectivity Atlas contains >1000 cortical injections. We manually inspected each injection, and selected 127 injections specifically targeting 15 cortical subregions (See Supplementary file 1 for selection details).

Specifically, at the time our analyses were performed, the AIBS Mouse Brain Connectivity Atlas contained 1029 cerebral cortex injections (Oh et al., 2014) which sampled the telencephalon. Here subregions of the isocortex, hippocampus, and amygdala are all broadly defined as telencephalic cortical areas that originate developmentally from the cortical plate, and separated into neocortex (FrA, M1/2, S1/2, Vis, Ptl, and Aud), mesocortex (AI/GI/DI, Rhi/Tem, LO/VO, PrL/MO, IL, dACC/vACC, and Rsp) and allocortex (Sub and Amyg) classes. Neocortex is primarily six-layered and comprised of the primary sensory and motor cortices. Mesocortex, also called the paralimbic cortex, is generally three-layered and is made up of associative subregions in frontal cortex as well as subregions at the interface between allocortex and neocortex, such as insular and perirhinal cortices. Allocortex is the evolutionarily oldest part of cortex, and comprised of piriform cortex, hippocampus and the subiculum (McGeorge and Faull, 1989). Although the amygdaloid complex has both telencephalic (pallial) and subpallial origins, it is situated within allocortex, between piriform cortex and the subiculum (Pabba, 2013). Being functionally related to both the hippocampus through the limbic system and the piriform cortex with olfactory processing (Novejarque et al., 2011), it was grouped here as part of the allocortex. Since that olfactory information does not project directly to the dorsal striatum and only very weakly to the ventral striatum and with olfactory tubercle not considered, olfactory areas and the piriform cortex were not included (McGeorge and Faull, 1989), leaving 957 injections. This was also checked through a search for olfactory to striatal projections in the AIBS Mouse Brain Connectivity Atlas (data not shown). These 957 injections include both wildtype and cell-type specific cre lines, 177 of these injections are in wildtype C57BL/6J animals. However, many of the wildtype injections spanned multiple cortical subregions and had insufficient subregion specificity to map projections. Therefore, three primary sets of cre lines were also included in the search: A930038C07Rik-Tg1-Cre, Rbp4-Cre_KL100, and Cux2-IRES-Cre. The cre lines were chosen to span cortical layers 2/3 (L2/3) and 5 (L5), so as not to bias the dataset towards intratelencephalic (IT) or pyramidal-tract (PT)-type corticostriatal projections (Harris and Shepherd, 2015; Kress et al., 2013), and contain injections in all of the cortical subregions analyzed. This added another 177 injections to the 177 wildtype injections, totaling 354 to choose from. No cortical layer 4 (L4) or layer 6 (L6) lines were chosen because they do not project to the striatum (Briggs, 2010). One injection each from Etv1-CreERT2, Gpr26-Cre_KO250, and Grp-Cre_KH288 mouse lines in auditory and insular cortices were used to supplement the lack of specific L5 or L2/3 injections in the AIBS connectivity atlas for the three primary cre lines described above (Supplementary file 1). The amygdala and hippocampus were primarily targeted by wildtype injections, but also required a different set of injections from cre lines since they have different gene expression patterns from neocortex and mesocortex. The metadata for each injection identifies the primary and secondary brain areas infected, which was used as a first screening process for subregion specificity before each brain was manually evaluated for injection targeting accuracy and specificity. Some small subregions were grouped with functionally similar areas if few or no specific injections could be identified. This includes the following grouping: LO/VO, dACC/vACC, Rhi/Tem, and AI/GI/DI (Figure 1a). Injections specific to multiple areas within a single large subregion, such as visual and somatosensory cortices were selected to insure full coverage of the entire volume, and were analyzed as a single group (e.g., injections in VISp, VISal, VISl, and VISam for visual cortex). In the end 127 injections were found to specifically target 15 subregions that spanned all striatal projecting subregions originating from the cortical plate. All areas contain at least one wildtype, one L2/3, and one L5 injection and contain eight injections on average, with considerable variability depending on the size of the subregion, with the fewest being infralimbic (IL) with three injections and most being somatosensory cortex (S1/2) with 21 injections (Supplementary file 1).

For hippocampal areas, while some injections included in this dataset had CA1 or CA3 as a primary target, only injections that at least partially covered the subiculum sent projections to the striatum (data not shown). For amygdalar areas, the primary volumes of the amygdala injections in this dataset are in the basolateral amygdalar nucleus (BLA), and basomedial amygdalar nucleus (BMA), but they also cover parts of the central nucleus of amygdala (CEA), posterior amygdalar nucleus (PA), medial amygdalar nucleus (MEA), and piriform-amygdalar area (PAA), areas which span both pallial and subpallial parts of the amygdaloid complex (Supplementary file 1).

The raw data processing methods used to generate the voxelized corticostriatal projection data and AIBS averaged template brain were described previously (Kuan et al., 2015).

### Photostimulation of defasciculated and fasciculated axons in the striatum to examine functional connectivity

Mice were injected at P14–16 with 10–20 nl of an AAV2/1 virus encoding ChR2-H134R-TdTomato (Addgene: 28017). Coronal brain slices were prepared 14 days later from mice anesthetized with an intraperitoneal injection of ketamine (13 mg/ml) /xylazine (1 mg/ml) (~0.01 ml/g body weight solution was injected) and perfused transcardially with ice cold ACSF containing (in mM): 127 NaCl, 25 NaHCO_3_, 25 D-glucose, 2.5 KCl, 1 MgCl_2_, 2 CaCl_2_, and 1.25 NaH_2_PO_4_, pH 7.25–7.35, ~310 mOsm, and bubbled with 95% O_2_/5% CO_2_. The brain was removed and placed into ice-cold cutting solution containing (in mM): 110 choline chloride, 25 NaHCO_3_, 25 D-glucose, 11.5 sodium ascorbate, 7 MgCl_2_, 3 sodium pyruvate, 2.5 KCl, 1.25 NaH_2_PO_4_, and 0.5 CaCl_2_. 300-μm-thick coronal slices were vibratome sectioned (Leica, Germany 1200 s). Slices were incubated in oxygenated ACSF for 45 min at 34°C, and then maintained in an oxygenated holding chamber at room temperature.

Electrophysiology recordings were performed during ChR2 photostimulation, as previously described (Hunnicutt et al., 2014; Mao et al., 2011). The excitatory postsynaptic currents (EPSC_sCRACM_) were recorded in voltage clamp (holding potentials were –70 mV or –75 mV) while blue light was stimulated the thalamic axons transfected with ChR2. Each map was repeated two to four times. The maps were averaged and a cell was counted as a positive responder if there was any excitatory postsynaptic current amplitude >6x the standard deviation of the baseline (Figure 1—figure supplement 1).

### Image integration and image analysis

The outline of the striatum was manually traced in each image set to generate a striatum mask. The front of the striatum was defined as the first slice containing the nucleus accumbens (NAc), where the anterior commissure (ac) separates from the rostral migratory stream. The border of the dorsal striatum was determined by the lateral ventricle (VL) and corpus callosum (cc). The ac was included in the striatum mask until it became medial of the VL. Posterior to the commissural part of ac, the ac formed the ventral border of the striatum. In posterior sections containing the globus pallidus extrenal segment (GPe) and the internal capsule, they were considered the medial border of the striatum.

To facilitate comparison across experiments and datasets, each experimental striatum mask was aligned to the striatum of the AIBS average template brain (Kuan et al., 2015). First, each section image was rotated about the anterior posterior (A-P) axis so that it was oriented vertically (i.e., roll rotation) based on manually selected midpoints and down-sampled to 25 µm per pixel (Figure 1—figure supplement 4a). Rotation of the images caused by an aberrant sectioning angle about the left-right (L-R) axis (i.e. a pitch rotation) was estimated using manually selected landmarks, and the rotation due to an aberrant sectioning angle about the dorsal-ventral (D-V) axis (i.e. a yaw rotation) was estimated using the center of mass of each hemisphere (Figure 1—figure supplement 4). The average template brain was rotated using these estimated angles to mimic the aberrant sectioning angle of the experimental brain. A center of mass curve was then generated from the striatum mask of this rotated average template brain, and the experimental brain sections were aligned to the rotated average template brain in the M-L and D-V axes. In the M-L axis, only the top half of the striatum was used to calculate the center of mass due to the variability in the ventral striatum masks. Additionally, the first several sections of the striatum (a variable number depending on D-V rotation angle) were aligned using the center of mass of the anterior commissure because the range of D-V sectioning angles made the shape of these sections too variable to implement a striatum center of mass alignment. In a case where a section displayed significant tissue damage, the section was skipped, and the sections before and after the damaged section were averaged to replace the damaged section for both the striatum mask, as well as the projection masks.

The full experimental striatum was scaled in the anterior-posterior (A-P) axis to fit the rotated average template brain based on the first and last section containing the corpus callosum crossing the midline. A linear scaling in the D-V axis was applied based on the average distance from the top of the striatum to the center of mass of the anterior commissure in the front several sections, and this scaling for sections posterior to the anterior commissure crossing the midline was based on the average distance from the top to the bottom of the striatum. Scaling in the M-L axis was determined by an average width of the dorsal striatum above the center of mass. The section images are then iteratively aligned to the rotated average template brain in the D-V axis using the anterior commissure for the first several sections, and the dorsal border of the striatum for posterior sections, and realigned in the M-L axis based on the center of mass of the top half of the striatum.

After these alignments, the experimental brains were rotated in all axes to align with the original coordinates of the average template brain, and then subjected to one more round of iterative alignment in each axis as described above. Finally, after visual inspection, if manual adjustments to the alignment were necessary, they were fed back to a point just before the average template brain is rotated to mimic the aberrant sectioning angle of the experimental brain, and the process is repeated. The corresponding thalamic projection masks were aligned concurrently with the striatum masks. The final result is the alignment of each experimental brain to the average template brain (Figure 1h and Figure 1—figure supplement 4b–c). Figure 1—figure supplement 4b–c show all of the aligned striatum masks overlaid at several coronal sections in the A-P axis for all experimental striatum masks.

Corticostriatal projections were identified in the AIBS images based on an AIBS custom image segmentation algorithm that identifies all fluorescent pixels and produced a full-resolution (0.35 µm/pixels) binary mask of positive pixels (Figure 1d). The images were then binned into 100 µm x100 µm x100 µm voxels, where the value of each voxel represents the fraction that contained positive fluorescence within that voxel. This data was used for the analysis of corticostriatal projections in the present study. Guided by the original images, we applied thresholds of 0.2, 0.05, and 0.005 to the voxelized data to localize the dense, moderate, and diffuse projections, respectively. Corticostriatal projection data were manually corrected to remove fluorescence resulting from fasciculated traveling axons that do not make synapses in the striatum, since the AIBS analysis did not vigorously distinguish traveling axons from axon terminals (Figure 1—figure supplement 2). The contaminating traveling axons were removed manually based on their stereotypic bundled and fasciculated morphology (similar to fasciculated thalamostriatal axons that are functionally evaluated in Figure 1—figure supplement 1) using custom MATLAB software.

For all injections in AIBS Mouse Brain Connectivity Atlas, the voxelized data was obtained from the AIBS and the preprocessed projection density data was obtained from the AIBS API (http://www.brain-map.org/api/index.html) (Research Recourse Identifier (RRID): SCR_005984) which contained the volume and density of projections to all brain regions defined in the AMBA ontology. This data was utilized in the present study to identify the density of corticothalamic projection in specific thalamic nuclei (Figure 6 and Figure 6—figure supplement 1, green) and corticocortical projections (Figure 7, Figure 6—figure supplement 2, Figure 6—figure supplement 3 and Figure 7—figure supplement 1). These data describe the density of projections in each cortical subregion and each thalamic nucleus. Since these cortical and thalamic subregions are well demarcated and do not contain bundled axons requiring manual removal, as in the striatum (Figure 1—figure supplement 2a–k), this data accurately represents the corticothalamic and corticocortical connectivity of each injection.

### Machine learning and human supervised process for excluding fasciculated traveling axons

To localize thalamostriatal projections and distinguish them from traveling thalamocortical axons, a machine-learning plugin for ImageJ, Trainable WEKA Segmentation (http://fiji.sc/Trainable_Weka_Segmentation) was used (Figure 1—figure supplement 3). To prepare the image sets for training, each image section containing striatum was background subtracted, a 12-pixel Gaussian filter was applied, and the striatum mask was used to limit the region of interest to only the striatal volume. The images were then split into single channels (red or green) and converted to an 8-bit grayscale format. The WEKA Segmentation program was manually trained to distinguish between three categories: (1) defasciculated axons that make synapses in the striatum, (2) fasciculated, or bundled axons that travel through the striatum to reach their final targets in the cortex, or (3) residual background fluorescence (Figure 2 and Figure 1—figure supplement 3e). Visually, fasciculated traveling axons could be identified as being highly directionally oriented and generally brighter than the defasciculated thalamostriatal projections, which have a diffuse, spidery appearance (Figure 1—figure supplement 1a–c). Since these morphological distinctions varied slightly for projections from different thalamic nuclei, separate training was required for each brain. For each channel, 3–6 sections (an average of 4) were used for training. The Trainable Weka Segmentation parameters were as follows; six image filters were selected, Entropy, Membrane Projections, Neighbors, Structure, and Variance. Classes were homogenized, and the other settings were left on their default values (membrane thickness: 1, membrane patch size: 19, minimum sigma: 1.0, maximum sigma: 16.0, classifier: fast random forest of 200 trees with two features per tree). Once the training was complete, the classifier was applied to the remaining ~80 sections of the brain containing the striatum, generating a probability map for each of the three features listed above, which conveys the certainty that a given pixel belonged to each of the three features. Only the defasciculated projection probability map was utilized (Figure 1—figure supplement 3f).

A threshold was selected for the defasciculated projection probability map and applied to the full probability map stack. This single-level threshold was chosen to encompass the largest possible region of correctly trained defasciculated projections throughout the striatum (Figure 2 and Figure 1—figure supplement 3g). Individual images were manually inspected for accuracies in projection identification during the Trainable Weka Segmentation process, and any inaccuracy was manually corrected in MATLAB using custom programs. The final output was a binary projection mask encompassing the full thalamostriatal projection for each injection.

### Confidence map generation

Confidence maps, which define the thalamic origin of projections, were created to determine the likelihood that regions of the thalamus sent projections to: (1) striatal volumes that contained corticostriatal projections originating from cortical subregions (Figure 4a), (2) striatal volumes that contained high- or low-diffuse corticostriatal input convergence (Figure 5—figure supplement 1c), and (3) striatal subdivisions generated by clustering voxels with common cortical input patterns (Figure 5e–f and Figure 5—figure supplement 3a). To control for alignment variability (~100 µm) across thalamus masks (Hunnicutt et al., 2014), an injection ‘core’ was produced by eroding the ‘full’ injection for each three-dimensional injection mask by 100 µm (Figure 2 and Figure 3—figure supplement 1). For each injection, a positive injection core adds one to the confidence level and a positive full injection adds one (Figure 3—figure supplement 1b–c,e). Similarly, negative injection cores subtract one from the confidence level, and a negative full injection subtracts one. Exception: full injections were only subtracted for the two easiest to meet criteria in each grouping method (Figure 3—figure supplement 1a, arrows, and 1d), and subsequent criteria only subtract negative injection cores as one. Figure 3—figure supplement 1 shows a simplified schematic of this process for case (1) listed above. A six level confidence map was generated by determining the inclusion of each injection in the following three groups; 10% of the diffuse target volume covered by the projection, 5% of the dense target volume covered by the projection, and 50% of the dense target volume covered by the projection (Figure 3—figure supplement 1g). Thalamic volumes occupied by the cores of injections that did not meet any of these criteria were set to zero. For cases (2) and (3), there was not projection density data, but instead binary volumes targeted by the thalamic projections, so the injection grouping was adjusted accordingly. For these groups, eight level confidence maps were created by determining the inclusion of each injection in the following four groups; 10% of the target volume covered by the projection, 10% of the projection volume within the target, 25% of the target volume covered by the projection, and 25% of the projection volume within the target. Thalamic volumes occupied by the cores of injections that did not meet any of these criteria were set to zero, and values of the final confidence maps below zero are also set to zero. The overall method was similar to that for case (1), as shown in Figure 6—figure supplement 1, except each injection is categorized based on the inclusion in each of the four groups listed above instead of the three groups shown in Figure 3—figure supplement 1.

### Voxel cluster analysis for striatal segmentation

Each voxel was assigned a point in a 15-dimensional space corresponding to the density of projections from each cortical subregion (Figure 5). The optimum distance metric was determined by comparing the cophenetic correlation coefficient across methods, and Spearman’s rank correlation metric was selected with a cophenetic coefficient of 0.78. This distance metric and an average linkage were used to perform cluster analysis on the striatal voxels. The maximum number of voxel clusters was determined by applying a threshold to the resulting dendrogram. The projection regions were similarly assigned a point in 25-dimensional space corresponding to the 25 nuclei, and clustered using the same method.

### Network diagrams of circuit convergence and connectivity

The chord diagrams illustrating corticocortical connectivity (Figure 7 and Figure 6—figure supplement 2b–p) were generated using a Circos plot with a ratio layout (Krzywinski et al., 2009). Since corticocortical connections may be either reciprocal or unilateral, ribbons joining them may have widths on one or both ends. Corticocortical connections are shown only for connections to or from a cortical subregion included in the indicated network, i.e. a primary convergent input to either the corticostriatal projection field (Figure 6—figure supplement 2) or the striatal subregion (Figure 7). For the corticostriatal projection fields, the convergence of one cortical subregion with one other cortical subregion was averaged across projection densities, i.e. the fraction of dense projections in the dense projection field, moderate projections in the moderate projection field, and diffuse projections in the diffuse projection for one cortical subregion with the corticostriatal projection field of each other cortical subregion. Corticocortical connections are indicated for projections with a density >15% in the target area, and primary convergent subregions are those where their projection fields converge with >50% the target projection field. Since the Amyg has broad projections throughout the striatum, it constituted a primary convergent input to all corticostriatal projection fields. However, in order to highlight unique interactions, the Amyg connections were left out of the corticocortical maps.

The network relationship diagrams shown in Figure 6—figure supplement 3 were created using an open source network analysis software program, Gephi (Bastian et al., 2009). The summary network diagram shown in Figure 6—figure supplement 2a is a manually modified version of a Gephi network diagram. The order of cortical nodes in each network diagram was based on the cortical subregion clustering shown in Figure 5b, the order of the striatal nodes was the same as for the cortical nodes, and the order of the thalamic nodes were based on their projection similarity, as shown in Figure 4d. Edges are shown for connections that are above a cutoff for each projection type: corticostriatal: projection density >15% in the target area; corticostriatal: projection fields converge with >50% the target projection field (as described for the chord diagrams above); thalamostriatal: thalamic nucleus with >20% of its volume contributing to the convergent projections; thalamocortical: thalamic nucleus with >20% of its volume contributing to projections to the indicated cortical subregion; corticothalamic: projections where >20% of the thalamic nucleus received projections from the corresponding cortical subregion.

For the cortico-thalamo-basal ganglia circuit, it is also worth noting that since the cortical subregions used to localize the thalamocortical projections may send corticostriatal projections to more than just the associated striatal subdivision, the thalamocortical data may over-represent the association with the striatal subdivision. However, this does not diminish the relationship seen between the thalamus and cortex for subdivision-specific networks in the circuit, since the thalamocortical inputs are going to a primary input to the striatal subdivision, but it may account for the excess of thalamocortical projections not associated with corresponding thalamostriatal projections in these networks (Figure 7e–f). Furthermore, the thalamostriatal confidence maps for each striatal subdivision are unrelated to the thalamostriatal confidence maps for cortical subregions since the striatal subdivisions may be either larger or smaller than the full projection fields of their corresponding primary cortical inputs.

### Electrophysiological recording and photostimulation

Brain slices were obtained from mice that were stereotaxically injected using methods similar to those used for the anatomical injections at postnatal day 16 with 10–20 nl of AAV serotype two expressing synapsin-CsChR-GFP, purchased from the University of North Carolina viral core (titer 4*10^12^ particles/ml) (Klapoetke et al., 2014). Injection coordinates were deduced from Figure 2a and Figure 4a (relative to bregma; along the anterior – posterior axis, with positive values anterior to bregma, along the medial – lateral axis relative to the midline, and along the dorsal – ventral axis relative to bregma in µm): d/vACC, 850, 200, 1750 and 1450; Vis, -3000, 2200, 600 and 300; Thal1, -50, 500, 3400; Thal2, -1000, 750, 3000. Batches of 4–6 mice were injected within one day, and care was taken to include all four subregions in each batch.

Coronal brain slices (300 µm) were prepared 14–21 days post-injection with ice cold KREBS buffer containing (in mM) 125 NaCl, 21.4 NaHCO_3_, 11.1 D-glucose, 2.5 KCl, 1.2 MgCl_2_, 2.4 CaCl_2_, 1.2 NaH_2_PO_4_, ~305 mOsm, supplemented with 5 µM MK-801 and oxygenated with 95% O_2_/5% CO_2_. Slices were incubated in oxygenated KREBS buffer supplemented with 10 µM MK-801 for 30 min at 33°C and then maintained in a holding chamber at 22–24°C. Recordings were performed at 32–33°C with oxygenated KREBS buffer containing GABA_A_- and GABA_B_-receptor antagonists, nicotinic and muscarinic acetylcholine receptor antagonists, a metabotropic glutamate receptor five antagonist, and an NMDA receptor antagonist. Two experimenters (BCJ and WTB) using two electrophysiology rigs performed whole-cell recordings; experimenters’ initials below note differences between experimental setups. There was no difference in results between experimenters, therefore all data were pooled. Oxygenated KREBS was supplemented with (in µM, purchased from Tocris unless noted): BCJ, GABA_B_-receptor antagonist CGP 52432 (10), GABA_A_-receptor antagonist SR 95531 (10), nicotinic acetylcholine receptor Mecamylamine (10), muscarinic acetylcholine receptor antagonist Scopolamine (10), metabotropic glutamate receptor five antagonist MPEP (0.3), NMDA receptor antagonist MK-801 (5); WTB, GABA_B_-receptor antagonist CGP 55845 (0.2), GABA_A_-receptor antagonist Picrotoxin (10, Sigma Aldrich), mecamylamine (1), muscarinic acetylcholine receptor antagonist atropine (0.1) and MPEP (0.3), pre-incubated in MK-801 (5).

Borosilicate pipettes (2.8–4 MΩ; Warner Instruments) were filled with potassium gluconate-based internal solution (in mM: 110 potassium gluconate, 10 KCl, 15 NaCl, 1.5 MgCl_2_, 10 Hepes, 1 EGTA, 1.8 Na_2_ATP, 0.38 Na_2_GTP, 7.8 phosphocreatine; pH 7.35–7.40; 290 mOsm). Putative MSNs were identified by their morphology and stereotypic physiological properties. Evoked excitatory postsynaptic currents (EPSCs) were recorded in whole-cell voltage-clamp mode at −75 mV holding potential. Recordings for Figure 8 and Figure 8—figure supplement 1 were targeted to a non-striated portion of the dorsomedial striatum between the lateral ventricle and the portion of the striatum containing fasciculated traveling axons.

Photostimulation was performed using a custom-made LED system, consisting of a 470 nm LED mounted on Olympus BX51WI microscopes, tuned to deliver between 0.1 and 2 mW (measured after 60x objective) 1 ms duration light pulses. For paired-pulse stimulation, two consecutive pulses at an interval of 50 ms were given and repeated every 20–40 s for at least five times. Repetitive stimulation consisted of 10 pulses at 20 Hz and was repeated every 20–40 s for at least seven times. Putative MSN with evoked EPSC of ≤−100 pA were included.

Data were acquired at 10 kHz using an Multiclamp 700B (BCJ) with an online 2 kHz low-pass filter (Molecular Devices) and *Ephus* software (www.ephus.org) or using an Axopatch 200A amplifier (Molecular Devices) and AxoGraph X software sampled at 20 kHz and filtered online with a 5 kHz low-pass filter (WTB). Data analysis was performed in Matlab, R (http://cran.r-project.org), Igor Pro (Wavemetrics), Excel (Microsoft), Axograph X, Origin7 (OriginLab) and Prizm 6 (GraphPad). Rise- and decay time were calculated based on 10% to 90% of EPSC peak value. For decay time calculation, the presence of a slow current was taken into account. Slow currents of single evoked EPSCs were calculated as the change in mean current (10 ms episode) at 40 ms post-stimulus relative to 10 ms pre-stimulus or, in the case of repetitive stimulation, 10 ms prior to the tenth stimulus over 10 ms before the first stimulus, and normalized to EPSC peak value. Charge transfer was calculated per stimulus over a 50 ms episode starting from the stimulus onset and normalized to the charge transfer evoked by the first stimulus. For data analysis, numbers of observations represent recorded cells from (# cells / # mice): d/vACC, 34/6; V1, 26/4; Thal1, 25/5; Thal2, 32/5.

Due to the injection site-specific innervation patterns to the striatum, injection sites were first inspected by the experimenters. Injected animals were excluded from analysis, when thal1 or thal2 injection produced tail-contamination in the d/vACC. Statistical comparisons were performed using Kruskal-Wallis test followed by *post-hoc* Dunn’s test with Bonferonni correction for multiple testing (Figure 8e,g, and Figure 8—figure supplement 1b–e) and two-way repeated measures ANOVA with *post-hoc* Tukey’s multiple comparisons test (Figure 8—figure supplement 1f–g). The results presented here do not show correlations with the light power used for photostimulation (data not shown).

### Retrobeads injections

Mice (P21) were injected with LumaFluor red or green beads (1:1 diluted in sterile PBS) in the dorsomedial (DMS) and posterior striatum (PS). Each animal received one injection in the DMS and two in the PS. Bead color – injection region combination was assigned randomly per animal. Injection coordinates were based on Figures 5, 15 nl bead volume per position was deposited at (relative to bregma; along the anterior – posterior axis, with positive values anterior to bregma, along the medial – lateral axis relative to the midline, and along the dorsal – ventral axis relative to bregma in µm): DMS, 1000, 1000, 3100; PS, −1600, 3250, 3700 and 3400. Mice were perfused with ice-cold 4% PFA in PBS 3 days post-injection. Brains were resected, post-fixed in 4% PFA in PBS overnight and subsequently stored in PBS at 4°. Coronal brain sections of 50 µm were produced on a vibratome and stained with 1:5000 Hoechst. Epifluorescent tiled images were made on an AxioImager N2 (Zeiss).

## Additional files

Supplementary file 1. AIBS viral injections included. The file includes the list of the cortical injections from AIBS that have passed our manual inspection using the criteria described in Materials and methods. **DOI:** http://dx.doi.org/10.7554/eLife.19103.028

### Major datasets

The following previously published datasets were used:

Hunnicutt B,Long B,Kusefoglu D,Gertz K,Zhong H,Mao T,2014,Thalamus-whole brain projection dataset,http://digitalcollections.ohsu.edu/projectionmap,Downsized images are freely available for download at OHSU Digital Collections (http://digitalcollections.ohsu.edu/). Full size images are available from T Mao lab upon request.
