# Supplementary material for: A comprehensive excitatory input map of the striatum reveals novel functional organization
Source: eLife. 2016 Nov 28;5:e19103. doi: 10.7554/eLife.19103 (PMC5207773; doi:10.7554/eLife.19103)
Supplement: Related texts

# Article commentary: Decision letter

**DOI:** 10.7554/eLife.19103.032

**Affiliations:** Washington University in St Louis, United States

In the interests of transparency, eLife includes the editorial decision letter and accompanying author responses. A lightly edited version of the letter sent to the authors after peer review is shown, indicating the most substantive concerns; minor comments are not usually included.

Thank you for submitting your article "A Comprehensive Excitatory Input Map of the Striatum Reveals Novel Functional Organization" for consideration by *eLife*. Your article has been reviewed by 3 peer reviewers, and the evaluation has been overseen by David Van Essen as the Senior Editor and Reviewing Editor. The following individuals involved in review of your submission have agreed to reveal their identity: David M Lovinger (Reviewer #1).

The reviewers have discussed the reviews with one another and the editor has drafted this decision to help you prepare a revised submission.

The reviewers recognized many major strengths of this study. They also identified a number of issues that will entail substantive revisions.

Essential revisions:

1) Insert a new figure to show the thalamostriatal projection in more detail. This is clearly a key point in the paper, but it seems like a logical step is missing.

2) Expand the first part of Figure 5 to show the relationship of thalamostriatal and thalamocortical maps in much more detail. Ideally, this would include examples from the 4 main sub-regions of striatum identified in Figure 4.

3) Split the last part of Figure 5 into a new figure in order to focus on the broader cortico-thalamo-basal ganglia loops in more detail. This is an interesting idea, but is quite underdeveloped.

4) Address the substantial differences between the present results and those of older corticostriatal studies (including those in rat as well as primate).

5) Examine the existing dataset to see if it supports or denies the Yeterian and Van Hoesen, (Brain Res, 139:43-63, 1978) hypothesis that cortical areas strongly connected with each other converge in the striatum.

6) Cite and discuss the recent paper by Hintiryan et al. (Nature Neuroscience, 2016). Many of the corticostriatal projection subdomains identified by these investigators show good overlap with the data in this manuscript, but there may be some areas of disagreement (e.g. the ectorhinal projections into dorsal striatum seem to differ a bit), and if so these should be discussed.

Encouraged but not required:

1) A small number of retrograde injections labeling select sub-regions of striatum in order to validate key claims about convergence of cortical and thalamic inputs.

2) As suggested by Reviewer #1, show that the oEPSC results are robust.

3) Consider including data on entorhinal projections to striatum. This is a highly interesting cortical area for a number of reasons, so it is not clear why it was not included.

Reviewer #1:

Hunnicutt and coworkers have developed a comprehensive map of the mouse cortico- and thalamostriatal projections (and even some data on thalamocortical and corticothalamic projections), and the overlap of the connections. This was accomplished using a combination of existing databases and new experiments. The authors combined this anatomical connectivity work with detailed computer based analyses of injection volumes, averaging of striatal volumes, and effective subtraction of fasciculated fibers of passage. These approaches were supplemented with high quality optogenetic/electrophysiogical experiments showing the location and properties of intriguing selected cortical and thalamic inputs. Overall this work represents a tour-de-force of structural and functional connectivity analysis that will be valuable to everyone studying the cortico-basal ganglia-thalamic circuits and their functions. The mapping of overlapping cortical and thalamic inputs to striatum is especially valuable. There are a few areas where data presentation might be improved, and I strongly urge the authors to think about ways that input pathways and especially convergent pathways could be represented in schematic or 3d formats that would be easier for readers to understand. There are also a few questions about some aspects of the data and some small corrections that are needed.

1) It would be helpful to have at least one figure or panel that included schematic drawings of a section of cortex, a section of striatum and a section of thalamus that would illustrate the convergence of inputs to the striatum, for at least one good example case. Ultimately, it would be great to have several such schematics, maybe posted online. A 3-dimensional plot of some sort would be even more useful, but admittedly difficult to put into the manuscript itself. All the necessary information is certainly present in the figures, it is just very hard to appreciate the overlap without some sort of direct visual representation.

2) There did not seem to be any data on entorhinal projections to striatum. This is a highly interesting cortical area for a number of reasons, so it is not clear why it was not included.

3) Why were the injections, tracing and recordings performed using such young animals? Is there reason to believe that no anatomical or physiological differences would be observed in adult brain?

4) Regarding the oEPSCs, did the paired-pulse and frequency responses and the sustained current vary with light stimulus intensity, duration or the amplitude of the initial oEPSC? The data certainly suggest differences in synaptic properties, but care should be taken to be sure that these differences are not due to differences in the level of channel rhodopsin expression or activation in different projections (also, are the scale bar values in panel C the same as in panel D?).

5) The authors need to cite and discuss the recent paper by Hintiryan et al. (Nature Neuroscience, 2016). Many of the corticostriatal projection subdomains identified by these investigators show good overlap with the data in this manuscript, but there may be some areas of disagreement (e.g. the ectorhinal projections into dorsal striatum seem to differ a bit), and if so these should be discussed.

6) In the second paragraph of the subsection “Corticostriatal projectome data overview”, the authors note that the striatum does not receive olfactory information, and cite a previous publication and the AIBS site. However, it is not clear if they include the olfactory tubercle in their definition of the striatum. It might be worth revising this statement to indicate that olfactory information does not go to dorsal striatum and only weakly to ventral striatum/accumbens, but that the olfactory tubercle that has many striatal-like properties does receive this input, but will not be the subject of this study. On a related subject, Hintiryan et al. report that piriform cortex projects to ventral/ventromedial striatum, and the Allen Brain connectivity site shows a weak projection to parts of the nucleus accumbens, so perhaps statements regarding this cortical subregion should be revised.

Reviewer #2:

This paper by Mao et al. examines how cortical and thalamic inputs innervate and define functional regions in the striatum. The authors combine a meta-analysis of a cortex data set from the Allen Institute (Oh et al. 2014) with further analysis of a thalamus data set from their own lab (Hunnicutt et al. 2014). They first establish that different parts of cortex and thalamus make unique projections into the striatum. They then show how the thalamic nuclei associated with thalamostriatal projections relate to the corticostriatal projections. Similarly, they establish how these thalamic nuclei relate to striatal subdivisions defined by corticostriatal inputs. They then show how these thalamostriatal maps relate to their published thalamocortical maps, in an attempt to begin to establish the presence and extent of cortico-thalamo-striatal loops. Finally, they also perform some slice physiology experiments to confirm cortico- and thalamo-striatal connections.

While I think there is a lot of interesting information in the paper, the presentation was often confusing, and more example data, analysis, and explanation are needed. It should be noted that a recent study also studied the cortico-striatal projectome in great detail (Hintiryan et al., 2016). In my view, the current paper is complementary, as it focuses on thalamostriatal projections and larger circuits. I think it has the potential to be a valuable resource, but several major points first need to be addressed.

1) The authors jump from mapping corticostriatal inputs in Figure 2 to a complex analysis of thalamic nuclei in Figure 3. Before making that jump, I would like to see more examples of the thalamostriatal projections and how they were analyzed.

2) Figure 4 shows how corticostriatal inputs define sub-regions of striatum, and how different thalamic nuclei project onto these different sub-regions. I think it would be valuable to experimentally validate this result by performing retrograde injections into striatum and showing which cortical and thalamic areas are labeled. For example, retrograde injections into the 4 clusters in Figure 4D should presumably label the same areas of cortex shown in Figure 4B/C and thalamus in Figure 4E/F.

3) The first part of Figure 5 is potentially interesting, but currently underdeveloped. I would like to see more examples from the different clusters that receive cortical and thalamic input shown in Figure 4D. For example, it would be interesting to see examples for dorsomedial, dorsolateral, ventromedial and very caudal striatum.

4) The last part of Figure 5, examining corticothalamic feedback connections, is very underdeveloped. It is important to show much more data on the corticothalamic projection, how it was processed, and how it fits into the paper. As it currently stands, I cannot agree that "these data provide a comprehensive picture of cortical and thalamic input integration in the cortico-thalamo-basal ganglia circuit".

5) In Figure 6, it seems like it would be much more interesting to validate some of the previously unexplored areas of the striatum that were identified in Figure 4D, and see examples for dorsomedial, dorsolateral, ventromedial and caudal striatum. Although it is not a big part of the paper, the authors should also provide some explanation for the slow EPSC, which could be an artifact due to excessive input. Ideally, they should show that this data is relatively invariant with light intensity.

6) The authors should discuss in detail how their work relates to Hintiryan et al., 2016.

Reviewer #3:

This paper shows how far we have come technically. The future of connectivity studies looks a lot like this paper. It is exciting to see a comprehensive approach to a large and diverse projection like the corticostriatal pathway.

However, the results make a stark contrast with the results from traditional methods of mapping the corticostriatal projection, and I have more confidence in the previous results. The results here indicate much less structure in the corticostriatal projection than expected, and it may be the projection is less structured in mice than in larger animals. Both the old and new methods (and primates and mice) have their strong and weak points. It is great to cover the entire cortex. In the previous generation of this kind of work it was never possible to do more than a fragment of the cortex in any one study. The positive side of the old primate work is that cortical regions were much better defined, and the projections were seen at higher resolution. Not only are functional regions larger in primates, but it was often possible to define regions functionally, rather than only by position on the cortex. This allowed injections to be placed far from functional boundaries, and even in functional subregions (e.g. hand area of somatosensory cortex). A lesson of those old papers was that cortical regions that are close to each other might be functionally disparate and have disparate projections in the striatum. That is, although they projected to the same general area of the striatum (e.g. the somatosensory cortex recipient region), injections in the hand and foot somatosensory cortex did not have convergent projections at the microscopic level, and so did not converge on single striatal neurons. My first concern about this study is that all of those principles learned from the primate work have been jettisoned, and a strictly spatial scheme is used. Because functional areas of the cortex in the mouse are so small, it is likely that most of these injections involve very different functional cortical regions with different projections in the striatum, and are being superimposed to make the projection look less specific than it is. The same could be said about the thalamic projections. Stated differently, if the cortex or thalamus contained an affine map of function, the lost resolution in the injections could be recovered by comparing nearby injections. If it is not, then this will not work. I think the previous work suggests that it is not.

One method that has served well in the past when functional mapping was unavailable was to look at the projections in the thalamus. Often, the precise set of cortical areas involved in a tracer injection can be inferred from its thalamic projections, as these have such sharp boundaries and corticothalamic relations are so well known.

One test of whether the results are showing us something really new or are just a blurred version of what we already know would be to find some new principle emerge, or to test an old one. I do not see any clear statement of a new principle. I think in this the authors may have missed an opportunity. One principle proposed by previous authors was that cortical areas that are strongly connected with each other converge in the striatum (Yeterian and Van Hoesen, Brain Res, 139:43-63, 1978). It seems to me that the information required to test this idea may be already available in the authors' data set, just by including corticocortical projections.

Declaring there to be a sensorimotor domain in the striatum is misleading. The somatosensory and motor systems are very closely connected and in the past many studies have shown that somatosensory and motor function may even overlap spatially in the cortex (of rodents). However, this does not extend to other sensory modalities. For example, visual cortex or its projections to the striatum do not go to the region called sensory by the authors. Throughout the paper, the word sensory seems specifically associated with the somatosensory cortex. I think the authors should replace the word 'sensory' in all cases in which it appears with 'somatosensory'. One thing we have learned from previous studies is that there is very little overlap between visual and somatosensory cortical projections to the striatum, and very little between visual and primary motor cortex. This is evident in Figure 2, but still, there is no recognition that Aud and Vis are sensory cortices. Their projections do not overlap much with motor cortex at all. What does this mean about the idea that all sensory input is 'sensorimotor'?

# Reply: Author response

**DOI:** 10.7554/eLife.19103.033

Essential revisions:

1) Insert a new figure to show the thalamostriatal projection in more detail. This is clearly a key point in the paper, but it seems like a logical step is missing.

We have now, in a new figure (Figure 3), added example images of the thalamostriatal projections and illustrated the steps of how the convergence of the corticostriatal and the thalamostriatal pathways was quantified. Briefly, the cortical inputs (Figure 2A_C) were first used to define a striatal subdivision of interest (Figure 3A-B). Then we localized the thalamic origins that project to this part of the striatum in the following steps. The thalamostriatal projection in individual sections were analyzed (Figure 3C-D) by using the method described in Figure 1—figure supplement 3. Thalamostriatal projections in the striatum were aligned across experimental mice as described in Figure 1—figure supplement 4 and their axonal innervation within the striatal subdivision, as defined by the cortical inputs, were determined (Figure 3E-G). The aligned thalamic projections in the striatum from individual injections were then categorized (Figure 3G and Figure 3—figure supplement 1). All injection-projection information for a given striatal subdivision was combined to derive a “thalamic confidence map”, which describes the thalamic subregions that innervate this striatal subdivision (example results shown in Figure 3H-K, and methods detailed in Figure 3—figure supplement 1). The confidence map for each striatal subdivision is analogous to a comprehensive retrograde tracing analysis from the striatal subdivision. Our method has the advantage that it is efficient (only computational efforts are involved after producing the original thalamic projection dataset), and allows the user to dynamically change the striatal subdivision of interests (e.g., the striatal subdivision receiving M1/2 inputs versus the subdivision receiving d/vACC inputs).

Along the same spirit of illustrating the key steps in our analyses using example images, we have also added Figure 2—figure supplement 1 for how the corticostriatal projection maps shown in Figure 2 were derived.

Overall, we hope these illustrations will help general readers to better understand our analyses.

2) Expand the first part of Figure 5 to show the relationship of thalamostriatal and thalamocortical maps in much more detail. Ideally, this would include examples from the 4 main sub-regions of striatum identified in Figure 4.

Due to the intertwining nature of individual injections/projections within the cortico-thalamo-basal ganglia loops, we find it difficult to completely separate the discussion and presentation of thalamostriatal and thalamocortical projections in the original Figure 5 with other projections in the loop without sacrificing clarity and accuracy. We have therefore taken the spirit of points 2 and 3 as a whole and added a new main figure (Figure 7) and two supplement figures (Figure 6—figure supplement 2 and Figure 6—figure supplement 3). Specifically related to the current point, greater details of thalamostriatal and thalamocortical projections related to the 4 primary striatal subdivisions identified in original Figure 4 (current Figure 5) are presented in Figure 7E and F. These new results are also described in detail in the main text (Results subsection “The cortico-thalamo-basal ganglia loop organization for clustered striatal subdivisions”; Methods subsection “Network diagrams of circuit convergence and connectivity”).

3) Split the last part of Figure 5 into a new figure in order to focus on the broader cortico-thalamo-basal ganglia loops in more detail. This is an interesting idea, but is quite underdeveloped.

As mentioned above, we have now added Figure 7 to further expand the concept of the cortico-thalamo-basal ganglia loop using our current thalamostriatal dataset, previously published thalamocortical dataset, and newly added corticocortical connectivity analysis based on data downloaded from the Allen Institute mouse connectivity atlas during the revision. We have also added Figure 6—figure supplement 2 and Figure 6—figure supplement 3 to show the network properties and the information flow in the cortico-thalamo-basal ganglia loop in the subregion-specific manner, with the highlight of thalamic nuclei innervated by GPi/SNr as the basal ganglia projection targets.

As discussed above, we did not split the previous Figure 5. Overall, we use S1/2 subregion as an example to explain the concept of the triangular loop (Figure 6C-D) and use the 4 thalamic subregions innervated by GPi/SNr as the way to tie the entire loop. The newly added Figure 7 is a natural extension of integrating now Figure 5 and Figure 6, as suggested by reviewer 2 in point 2. We hope that Figure 7, together with the full loop maps presented in Figure 6—figure supplement 1, and new Figure 6—figure supplement 2 and Figure 6—figure supplement 3 will provide readers a more detailed comprehension of the cortico-thalamo-basal ganglia loop circuit features. A detailed description of these features is also now included in the main text (Results subsection “The cortico-thalamo-basal ganglia loop organization for clustered striatal subdivisions”; Methods subsection “Network diagrams of circuit convergence and connectivity”).

4) Address the substantial differences between the present results and those of older corticostriatal studies (including those in rat as well as primate).

We have now devoted major text contents to discuss our finding in relationship to the available literature in rat and primate. As reviewer 3 accurately stated, the advantages of our study are two folds. First, because mouse brains are small, we were able to cover all cortical subregions and > 93% volume of the thalamus with individual injections of small (500-600 µm) sizes, and image the entire projections of every injection. The level of comprehensiveness in documenting thalamic projections has not been previously achieved in any mammalian species. Second, the comprehensiveness of the dataset allow us to perform quantitative analyses that are only possible with full dataset, but not with example images. For example, conventional descriptions of thalamic projections are mostly based on thalamic nucleus demarcations which, when compared to cats or primates, are less distinct and sometimes ambiguous in mice (Jones 2007, pg 52). As better stated in the textbook by Sherman and Guillery (2006), ‘The concept of the thalamic nucleus as a single structural, functional, and connectional entity has barely survived advancing techniques and new information. We stay with the thalamic nuclei as one of our prime analytical tools because, as yet, we have little to use in its place’. In our studies, we take the unique opportunity to establish nucleus-independent thalamic projection maps by using model thalamus based on averaging 70-100 mouse brains (Hunnicutt et al., 2014).

The caveats of our data and analyses were also pinpointed by reviewer 3: because the mouse brain is small, the definition of the subregion that gives the projections might be vague and ambiguous. That could be the source of the variation. This is true both for defining cortical injections and thalamic injections. For thalamic injections, as stated above, we took full advantage of the high *n* numbers we could get and used overlapping injections to increase the resolution beyond individual injection volumes (Hunnicutt et al., 2014). This algorithm is the operational expression of what reviewer 3 mentioned as ‘the lost resolution……recovered by comparing nearby injections’. Furthermore, our confidence maps are statistical by nature and therefore, are difficult to be compared to the traditional anatomical tracing just by numbers. For cortical injections, we integrated Allen Institute data but with strict criteria for selecting the injections within a given subregion (Methods). However, unlike in the primate literature, the definition of certain cortical subregions themselves can be controversial in mice (e.g., for M1 and M2, see Mao et al., 2011) and even with the ones that have clear functional definitions, the physical landmarks are often lacking to clearly demarcate them. We did the best practice possible by sticking to the existing mouse atlas using visible landmarks and by being consistent in injection selection process. Even with great care, the lack of sufficient demarcating landmarks may contribute to variability. We are also limited to what is available in AIBS database for cortical injections which is already the best effort available with current technology using over 1600 brains for establishing the model brain to assign the subregions. Because these cortical injections are mostly not-overlapping, we could not employ similar resolution improving algorithms as we did with thalamic injections (i.e., comparing to the neighbors). Despite these caveats, given that the mouse is an important model system for studying brain circuitry, our effort will provide a framework to examine the subregion-specific circuits in the mouse striatum.

Two technical notes are also important regarding why our datasets are different from certain traditional data. First, the high resolution imaging is crucial in our analyses because it allowed us to specify the portions of the striatum where there are a lack of projections, and we took consideration of the negative data (lack of projections) when localizing projection origins in the thalamus (see Methods and Figure 3—figure supplement 1). In contrast, most of traditional studies do not discuss any lack of projections due to lack of full brain images, and very few studies make full projections available. The way we categorized and quantified cortical projections requires comprehensive datasets (Figure 2, Figure 2—figure supplement 1, Figure 2—figure supplement 2, Figure 5—figure supplement 1 and Figure 7—figure supplement 1). With such a comprehensive map, any specific element of the circuits will need to be tested functionally in the future to have a thorough comparison across species. Second, that there are two distinct projection patterns of corticostriatal axons, a localized dense core projection and a diffuse projection that generally spans a wider area than the dense projections (Mailly et al., 2013). Many previous mapping studies preferentially focused on the dense projections, particularly when reporting a summary result of several tracing experiments. In our data, we mapped both the dense and diffuse projections, and this revealed some previously underappreciated convergence patterns, such as the diffuse somatosensory-motor inputs to a portion of the limbic striatum (mid-dark blue, Figure 5, 15 clusters) (Draganski et al., 2008), and the widespread diffuse projections of LO/VO to nearly the entire striatal volume (Figure 2). Our dense projection results are highly consistent with the corticostriatal projection distributions reported in the literature (Gruber and McDonald 2012), and studies that separate the dense and diffuse projections describe similarly widespread diffuse projections (Mailly et al., 2013; Haber et al., 2006).

Finally, we also cannot rule out occasional circuit differences at mesoscopic resolution across species due to parallel evolution, as has been found from time to time even between rat and mice (e.g., Schwarz et al., 2015).

We have added the above points in our Discussion.

5) Examine the existing dataset to see if it supports or denies the Yeterian and Van Hoesen, (Brain Res, 139:43-63, 1978) hypothesis that cortical areas strongly connected with each other converge in the striatum.

We have now added analyses to address the hypothesis proposed by Yeterian and Van Hoesen. We first downloaded corticocortical projection data from Allen Institute of Brain Science mouse atlas API and then analyzed projection density between cortical subregions (Figure 6—figure supplement 2). We found evidence of both types of connection patterns, i) for some cortical subregions, they interconnect with subregions that do not converge in the striatum with, ii) some are highly connected if they converge in the striatum (Figure 6—figure supplement 2B-P and Figure 7—figure supplement 1D-H). The first type seems more prevailing. Interestingly, the highly interconnected ones are FrA, M1/2, S1/2, and AI forming highly recurrent networks with convergent subregions, which may be related to highly coordinated functions of somatosensori-motor integration.

Along this line, we also further integrated cortiocortical connectivity data into our cortico-thalamo-basal ganglia loop analyses (Figure 6—figure supplement 3 and Figure 7—figure supplement 1).

6) Cite and discuss the recent paper by Hintiryan et al. (Nature Neuroscience, 2016). Many of the corticostriatal projection subdomains identified by these investigators show good overlap with the data in this manuscript, but there may be some areas of disagreement (e.g. the ectorhinal projections into dorsal striatum seem to differ a bit), and if so these should be discussed.

We have now added the citation of Hintiryan et al. and discussed the related contents. Hintiryan et al. uses an anterograde tracing dataset from cortical injections to illustrate the corticostriatal circuits and demonstrated the usefulness of large scale mesoscopic projection mapping to study ‘circuitry-specific connectopathies’. This study and ours share the same spirit in terms of using comprehensive mesoscopic cortical projections in the striatum to understand the logic of the striatum circuit. At the same time, our study also differs from Hintiryan et al. as we include the thalamostriatal pathway and eventually integrate information regarding cortico-cortical, corticothalamic and thalamocortical circuits. These two studies also emphasize the complementary aspects of the striatal circuits, as detailed below.

First, Hintiryan et al. paper focused on cortical inputs to the dorsal striatum. In addition to cortico-dorsal striatum data, our study also includes cortico-ventral striatum projections and the projections from the thalamus to the entire striatum. Our thalamostriatal dataset is of particular interest because thalamostriatal data for mouse is scarce in the literature and the circuits are much less understood compared to the corticostriatal pathways. As an example of conclusions unique to our study, the thalamic origins of the projections to the dorsal and ventral striatum, respectively, show a large degree of complementary patterns (Figure 5 and Figure 5—figure supplement 2). Conceptually, we also only use corticostriatal data as the first step, as shown in new Figure 3, to illustrate the convergence of the corticostriatal and the thalamostriatal pathways. The completeness of our dataset allows us to illustrate the complete cortico-thalamo-basal ganglia loop by further incorporating corticocortical and thalamocortical connectivity, which is not possible with a corticostriatal only dataset.

Second, Hintiryan et al. identified 29 distinct striatal domains, which are useful for understanding the sub-organizations of the striatum and studying their functions. However, whether there are hierarchical organizations between these domains is less clear. In our analysis, we described a fourth primary subdivision within the posterior striatum, which is in parallel to the conventional dorsomedial, dorsolateral and ventral striatal subdivision. By lowering the clustered dendrogram thresholds, increasing numbers (we present up to 15, but the number can be more with even lower thresholds) of subdivisions can be identified and described, but most of these smaller subdivisions are associated with one primary striatal subdivision, the dorsomedial subdivision, and this is consistent with the associative function of this subdivision (Figure 5D).

Third, while Hintiryan et al. examined two mouse lines and demonstrated the subregion-specific changes in pathological conditions, we provide physiology/optogenetics mediated functional confirmation of the anatomically defined pathways. Importantly, we showed that the subregion-specific input pathways have distinct plasticity properties and this finding could reconcile the apparent discrepancy in the field (Discussion).

Regarding the corticostriatal projections, our data is overall consistent with Hintiryan et al. As we discuss both above (Essential revisions #4) and below (Encouraged but not required #3), the caveats associated with mouse cortical injection is the difficulty in assigning precisely the subregions being injected. This in turn can make it difficult in cases to evaluate whether individual injections are in and only within a claimed cortical subregion. This applied to both work. For this reason, we were very conservative in including any injections. For example, we included <10% of all published AIBS cortical injections, and we did not take entorhinal as an individual group because almost all AIBS injections in this area also covered either entorhinal, perirhinal, or some of temporal cortex. We instead included entorhinal in Rhi/Tem group (subsection “Corticostriatal projectome data overview”, second paragraph). The small differences between the two studies in corticostriatal inputs might potentially be from jitters in assigning or grouping the cortical subregions.

These above points are now included in the Discussion.

Encouraged but not required:

1) A small number of retrograde injections labeling select sub-regions of striatum in order to validate key claims about convergence of cortical and thalamic inputs.

We have now performed retrograde injection experiments by using retrobeads to target 2 striatal subdivisions described in Figure 5, the most dorsomedial subdivision of the striatum and the posterior striatum, as suggested by reviewer 2 (Figure 5—figure supplement 3). All retrogradely labeled neurons are found within the boundaries of the cortical and thalamic subregions illustrated in the manuscript for projecting to the corresponding striatal subdivision. Please note that the beads do not label the whole striatal subdivision, and therefore does not retrograde label all cortical and thalamic input regions. This is expected since we intentionally used very small injections so that there would be no contaminating tails (we excluded animals that we could detect obvious injections tails).

2) As suggested by Reviewer #1, show that the oEPSC results are robust.

We have now added analysis to show that oEPSC results are robust. The discussion below also refers to Author response image 1 presented in the response to review 1 #4.

**Author response image 1.** **DOI:** http://dx.doi.org/10.7554/eLife.19103.029

Reviewer 1brought up one of the most important parameters in any channelrhodopsin-related quantitative comparison. We were fully aware of the ‘effective’ expression variations across animals. For this reason, our viral titers, infection time etc. parameters were the same for all four groups (we kept them even from the same batch of virus). Even with the best experimental effort, there could be intrinsic factors that give rise to different ‘effective’ expression levels for certain groups. Therefore, we plotted light power used for all four groups (Thal1, Thal2, Vis and d/vACC) and there is no overall difference in light power used among the four groups (panel A in Author response image 1, light power used for Thal1 = 1.157 ± 0.141 mw; Thal2 = 1.475 ± 0.136 mw; Vis = 1.589 ± 0.122 mw; d/vACC = 1.275 ± 0.075 mw, mean ± SEM, Kruskall-Wallis with post-hoc Dunn’s test, *P_(overall)_*= 0.09, *Χ2* = 6.54); we also plotted PPR over the light power and there is no correlation between the PPR and the light power (R^2^ for Thal1, Thal2, Vis and d/vACC were all < 0.045, panel C). Similarly, we also plotted EPSC over light power for single light stimulation and repetitive light stimulation (panel B and D). Our findings regarding the functional heterogeneity of striatal inputs are relative measures obtained within each recording, namely paired-pulse ratio and relative standing current to EPSC amplitude size. Since there is no correlation between PPR or relative standing current with light power, the observed heterogeneity is a feature of the biological system.

We want to emphasize that this set of experiments were carried out in two different setups and performed by two independent experimenters (pink and blue dots in the attached plot represent two independent experimenters) to minimize this types of bias in channelrhodopsin experiments, the results obtained from the two setups gave the same conclusion (the data in the manuscript was pooled data).

3) Consider including data on entorhinal projections to striatum. This is a highly interesting cortical area for a number of reasons, so it is not clear why it was not included.

Our data do include entorhinal (ENT) inputs (e.g. injection number 142656218 and 182794184 listed in Table 1). However, they were grouped as part of the Rhi/Tem group (previously named Ect/Tem group). Two AIBS injections (#113226232 and #263974698) that are claimed to be solely ENT, also hit perirhinal cortex when we examined the original data. From our inspection, injection #126116848 is the only one that is specific within ENT. Therefore, rhinal cortical subregions were grouped together. To avoid confusion, we renamed this group Rhi/Tem which includes all rhinal and temporal cortical subregions. This is now we clarified in Methods (subsection “Corticostriatal projectome data overview”, second paragraph), Table 1 and Table 2.

Reviewer #1:

Hunnicutt and coworkers have developed a comprehensive map of the mouse cortico- and thalamostriatal projections (and even some data on thalamocortical and corticothalamic projections), and the overlap of the connections. This was accomplished using a combination of existing databases and new experiments. The authors combined this anatomical connectivity work with detailed computer based analyses of injection volumes, averaging of striatal volumes, and effective subtraction of fasciculated fibers of passage. These approaches were supplemented with high quality optogenetic/electrophysiogical experiments showing the location and properties of intriguing selected cortical and thalamic inputs. Overall this work represents a tour-de-force of structural and functional connectivity analysis that will be valuable to everyone studying the cortico-basal ganglia-thalamic circuits and their functions. The mapping of overlapping cortical and thalamic inputs to striatum is especially valuable. There are a few areas where data presentation might be improved, and I strongly urge the authors to think about ways that input pathways and especially convergent pathways could be represented in schematic or 3d formats that would be easier for readers to understand. There are also a few questions about some aspects of the data and some small corrections that are needed.

1) It would be helpful to have at least one figure or panel that included schematic drawings of a section of cortex, a section of striatum and a section of thalamus that would illustrate the convergence of inputs to the striatum, for at least one good example case. Ultimately, it would be great to have several such schematics, maybe posted online. A 3-dimensional plot of some sort would be even more useful, but admittedly difficult to put into the manuscript itself. All the necessary information is certainly present in the figures, it is just very hard to appreciate the overlap without some sort of direct visual representation.

We have now added a new figure (Figure 3) that walks readers step-by-step through the method by which the convergence data are derived. Both schematic and example images of injections/projections are quantified. Briefly, the cortical inputs (Figure 2A-C) were first used to define a striatal subdivision of interest (Figure 3A-B). Then we localized the thalamic origins that project to this part of the striatum in the following steps. The thalamostriatal projection in individual sections were analyzed (Figure 3C-D) by using the method described in Figure 1—figure supplement 3. Thalamostriatal projections in the striatum were aligned across experimental mice as described in Figure 1—figure supplement 4 and their axonal innervation within the striatal subdivision, as defined by the cortical inputs, were determined (Figure 3E-G). The aligned thalamic projections in the striatum from individual injections were then categorized (Figure 3G and Figure 3—figure supplement 1). All injection-projection information for a given striatal subdivision was combined to derive a “thalamic confidence map”, which describes the thalamic subregions that innervate this striatal subdivision (example results shown in Figure 3H-K, and methods detailed in Figure 3—figure supplement 1). The confidence map for each striatal subdivision is analogous to a comprehensive retrograde tracing analysis from the striatal subdivision. Our method has the advantage that it is efficient (only computational efforts are involved after producing the original thalamic projection dataset), and allows the user to dynamically change the striatal subdivision of interests (e.g., the striatal subdivision receiving M1/2 inputs versus the subdivision receiving d/vACC inputs).

We agree with the reviewer that 3-D plots would be helpful. Data visualization at this scale is challenging and by itself is a rapidly developing field actively pursued by computer scientists and bioinformatists besides biologists. With the current resource we have, presenting the data in 3-D will be beyond the scope of this manuscript. However, along the spirit of the suggestion, we have made efforts in better presenting the data in reduced dimensionality (Figure 6—figure supplement 2, Figure 6—figure supplement 3, and Figure 7—figure supplement 1), and have added more example images to help illustrating our analysis algorithms (Figure 2—figure supplement 1). Many users of our data are used to 2D presentation and we hope these 2D plots might help them navigate specific circuits of interest.

2) There did not seem to be any data on entorhinal projections to striatum. This is a highly interesting cortical area for a number of reasons, so it is not clear why it was not included.

As discussed earlier, our data do include entorhinal (ENT) inputs (e.g. injection number 142656218 and 182794184 listed in Table 1). However, they were grouped as part of the Rhi/Tem group (previously named Ect/Tem group). Two AIBS injections (#113226232 and #263974698) that are claimed to be solely ENT, also hit perirhinal cortex when we examined the original data. From our inspection, injection #126116848 is the only one that is specific within ENT. Therefore, rhinal cortical subregions were grouped together. To avoid confusion, we renamed this group Rhi/Tem which includes all rhinal and temporal cortical subregions. This is now clarified in Methods (subsection “Corticostriatal projectome data overview”, second paragraph), Table 1 and Table 2.

3) Why were the injections, tracing and recordings performed using such young animals? Is there reason to believe that no anatomical or physiological differences would be observed in adult brain?

The thalamic injected mice were sacrificed ~1 month age. This age was chosen mainly based on our experience with physiological characterizations of the thalamocortical projections with the consideration of two aspects: the technical consideration and the biological consideration. On the technical part, the cortical slices are more robust, and pyramidal neurons, especially layer 5 pyramidal neurons, are much more healthy in young adult mice compared to older mice. So, to provide cortical recordings to validate the thalamocortical projections, as we have done in Hunnicutt et al. 2014 paper, we chose the age (P14-18) that we could do consistent stereotaxic injections and after two weeks of viral infection, we could still have high success rate of cortical neuron recordings. Although the slice health is less of an issue for the striatum, we aimed the injections to accommodate both thalamocortical and thalamostriatal pathways for physiology. On the biological part, the motor and sensory areas are considered functionally and structurally matured by our time-point (P30) and this time point is widely used to investigate sensorimotor integration (e.g., Van Eden & Uylings, 1985 and Weiler et al., 2008), although the frontal area is still dynamic around this time. In our previous work (Hunnicutt et al., 2014), we tested whether there may be large rearrangements in thalamocortical projection distributions, especially the thalamo-frontal projections in young adult mice. We found that thalamocortical projections from at least 24 nuclei have reached the frontal area and form functional synapses by P30, with all frontal subregions being contacted by at least 5 thalamic nuclei. Since the frontal subregions innervated by each nucleus are comparable to those seen in the adult rat, our data suggest that the majority of thalamocortical projections to the frontal area have reached their final targets by P30 in mouse. Therefore, the behavioral changes during young adult rodents are more likely due to local refinements and synaptic pruning, rather than larger rearrangements in thalamocortical projection distributions to frontal subregions.

The reviewer’s comment also brought up a really interesting question: would anatomical and/or physiological connectivity be different in older mouse brains at the mesoscopic resolution. This question is definitely worth testing but is beyond the scope of the current manuscript.

4) Regarding the oEPSCs, did the paired-pulse and frequency responses and the sustained current vary with light stimulus intensity, duration or the amplitude of the initial oEPSC? The data certainly suggest differences in synaptic properties, but care should be taken to be sure that these differences are not due to differences in the level of channel rhodopsin expression or activation in different projections (also, are the scale bar values in panel C the same as in panel D?).

The reviewerbrought up one of the most important parameters in any channelrhodopsin-related quantitative comparison. We were fully aware of the ‘effective’ expression variations across animals. For this reason, our viral titers, infection time etc. parameters were the same for all four groups. Even with the best effort, there could be intrinsic factors that give rise to different ‘effective’ expression levels for certain groups. Therefore, we plotted light power used for all four groups (Thal1, Thal2, Vis and d/vACC) and there is no overall difference in light power used among the four groups (panel A in Author response image 1, light power used for Thal1 = 1.157 ± 0.141 mw; Thal2 = 1.475 ± 0.136 mw; Vis = 1.589 ± 0.122 mw; d/vACC = 1.275 ± 0.075 mw, mean ± SEM, Kruskall-Wallis with post-hoc Dunn’s test, *P_(overall)_*= 0.09, *Χ2* = 6.54); we also plotted PPR over the light power and there is no correlation between the PPR and the light power (R^2^ for Thal1, Thal2, Vis and d/vACC were all < 0.045, panel C). Similarly, we also plotted EPSC over light power for single light stimulation and repetitive light stimulation (panel B and D). Our findings regarding the functional heterogeneity of striatal inputs are relative measures obtained within each recording, namely paired-pulse ratio and relative standing current to EPSC amplitude size. Since there is no correlation between PPR or relative standing current with light power, the observed heterogeneity is a feature of the biological system.

We want to emphasize that this set of experiments were carried out in two different setups and performed by two independent experimenters (pink and blue dots in the attached plot represent two independent experimenters) to minimize this types of bias in channelrhodopsin experiments, the results obtained from the two setups gave the same conclusion (the data in the manuscript was pooled data).

We decided not to put these plots as a figure in the manuscript. This is because although it is an important point, channelrhodopsin is more of a standard reagent now. We do state this point in the manuscript. We wrote: ‘The results presented here do not show correlations with the light power used for photostimulation (data not shown).’

We now change the label for scales for both panels C and D and as the reviewer indicated, they are the same labels.

5) The authors need to cite and discuss the recent paper by Hintiryan et al. (Nature Neuroscience, 2016). Many of the corticostriatal projection subdomains identified by these investigators show good overlap with the data in this manuscript, but there may be some areas of disagreement (e.g. the ectorhinal projections into dorsal striatum seem to differ a bit), and if so these should be discussed.

See our response to Essential revision #6.

6) In the second paragraph of the subsection “Corticostriatal projectome data overview”, the authors note that the striatum does not receive olfactory information, and cite a previous publication and the AIBS site. However, it is not clear if they include the olfactory tubercle in their definition of the striatum. It might be worth revising this statement to indicate that olfactory information does not go to dorsal striatum and only weakly to ventral striatum/accumbens, but that the olfactory tubercle that has many striatal-like properties does receive this input, but will not be the subject of this study. On a related subject, Hintiryan et al. report that piriform cortex projects to ventral/ventromedial striatum, and the Allen Brain connectivity site shows a weak projection to parts of the nucleus accumbens, so perhaps statements regarding this cortical subregion should be revised.

Our original statement did not mean to include olfactory tubercles. We now revised the statement in Methods regarding the olfactory processing to make this clearer.

We wrote: ‘Since that olfactory information does not project directly to the dorsal striatum and only very weakly to the ventral striatum and with olfactory tubercle not considered, olfactory areas and the piriform cortex were not included (McGeorge and Faull, 1989), leaving 957 injections’.

Reviewer #2:

[…]

While I think there is a lot of interesting information in the paper, the presentation was often confusing, and more example data, analysis, and explanation are needed. It should be noted that a recent study also studied the cortico-striatal projectome in great detail (Hintiryan et al., 2016). In my view, the current paper is complementary, as it focuses on thalamostriatal projections and larger circuits. I think it has the potential to be a valuable resource, but several major points first need to be addressed.

1) The authors jump from mapping corticostriatal inputs in Figure 2 to a complex analysis of thalamic nuclei in Figure 3. Before making that jump, I would like to see more examples of the thalamostriatal projections and how they were analyzed.

To add more example data and to improve the presentation and logic flow, we have now added the following full figures: Figure 3 and Figure 2—figure supplement 1 for example images; Figure 3 and Figure 1— figure supplement 2 for explaining the logic of how an analysis is performed; Figure 7, Figure 6— figure supplement 2, and Figure 6— figure supplement 3 to better illustrate circuit properties. We agreed with the reviewer that the original manuscript had a gap in how we went from corticostriatal pathways to the thalamostriatal convergence. We have now added a new figure (Figure 3) that walks readers step-by-step through the method by which the convergence data are derived. Briefly, the cortical inputs (Figure 2A-C) were first used to define a striatal subdivision of interest (Figure 3A-B). Then we localized the thalamic origins that project to this part of the striatum in the following steps. The thalamostriatal projection in individual sections were analyzed (Figure 3C-D) by using the method described in Figure 1—figure supplement 3. Thalamostriatal projections in the striatum were aligned across experimental mice as described in Figure 1—figure supplement 4 and their axonal innervation within the striatal subdivision, as defined by the cortical inputs, were determined (Figure 3E-G). The aligned thalamic projections in the striatum from individual injections were then categorized (Figure 3G and Figure 3—figure supplement 1). All injection-projection information for a given striatal subdivision was combined to derive a “thalamic confidence map”, which describes the thalamic subregions that innervate this striatal subdivision (example results shown in Figure 3H-K, and methods detailed in Figure 3—figure supplement 1). The confidence map for each striatal subdivision is analogous to a comprehensive retrograde tracing analysis from the striatal subdivision. Our method has the advantage that it is efficient (only computational efforts are involved after producing the original thalamic projection dataset), and allows the user to dynamically change the striatal subdivision of interests (e.g., the striatal subdivision receiving M1/2 inputs versus the subdivision receiving d/vACC inputs). We hope this closed the missing logic gap that the reviewer was concerned about.

Along the same spirit of illustrating the key steps in our analyses using example images, we have also added Figure 2—figure supplement 1 for how the corticostriatal projection maps shown in Figure 2 were derived.

2) Figure 4 shows how corticostriatal inputs define sub-regions of striatum, and how different thalamic nuclei project onto these different sub-regions. I think it would be valuable to experimentally validate this result by performing retrograde injections into striatum and showing which cortical and thalamic areas are labeled. For example, retrograde injections into the 4 clusters in Figure 4D should presumably label the same areas of cortex shown in Figure 4B/C and thalamus in Figure 4E/F.

See our response to Encouraged but not required #1.

3) The first part of Figure 5 is potentially interesting, but currently underdeveloped. I would like to see more examples from the different clusters that receive cortical and thalamic input shown in Figure 4D. For example, it would be interesting to see examples for dorsomedial, dorsolateral, ventromedial and very caudal striatum.

We have now added a new figure (Figure 7) to expand the concept of the cortico-thalamo-basal ganglia loop using our current thalamostriatal dataset, previously published thalamocortical and corticothalamic datasets, and newly added corticocortical connectivity analysis based on data downloaded from the Allen Institute mouse connectivity atlas during the revision. Greater details of thalamostriatal and thalamocortical projections related to the 4 primary striatal subdivisions identified in original Figure 4 (current Figure 5) are presented in Figure 7E and F. These new results are also described in detail in the main text (Results subsection “The cortico-thalamo-basal ganglia loop organization for clustered striatal subdivisions”; Methods subsection “Network diagrams of circuit convergence and connectivity”). Overall, we use S1/2 subregion as an example to explain the concept of the triangular loop (Figure 6C-D) and use the 4 thalamic subregions innervated by GPi/SNr as the way to tie the entire loop. The new Figure 7 is a natural extension of integrating now Figure 5 and Figure 6. The full loop maps in similar format to Figure 6B-C are presented in Figure 6—figure supplement 1. A detailed description of these features is also now included in the main text.

4) The last part of Figure 5, examining corticothalamic feedback connections, is very underdeveloped. It is important to show much more data on the corticothalamic projection, how it was processed, and how it fits into the paper. As it currently stands, I cannot agree that "these data provide a comprehensive picture of cortical and thalamic input integration in the cortico-thalamo-basal ganglia circuit".

We have now included datasets for thalamocortical, thalamostriatal, corticothalamic and corticocortical pathways. As discussed above, we have added a new main figure (Figure 7) and two supplement figures (Figure 6—figure supplement 2 and Figure 6—figure supplement 3) show the network properties and the information flow in the cortico-thalamo-basal ganglia loop in the subregion-specific manner, with the highlight of thalamic nuclei innervated by GPi/SNr as the basal ganglia projection targets.

Overall, we kept S1/2 subregion as an example to explain the concept of the triangular loop (Figure 6C-D) and use the 4 thalamic subregions innervated by GPi/SNr as the way to tie the entire loop. In these figures, corticothalamic projections are grouped across the cortical subregions that form the primary inputs of each of the 4 striatal subdivisions, as determined in Figure 7—figure supplement 1. We hope that Figure 7, together with the full loop maps presented in Figure 6—figure supplement 1, and new Figure 6—figure supplement 2 and Figure 6—figure supplement 3 will provide readers a more detailed comprehension of the cortico-thalamo-basal ganglia loop circuit features. A detailed description of these features is also now included in the main text.

5) In Figure 6, it seems like it would be much more interesting to validate some of the previously unexplored areas of the striatum that were identified in Figure 4D, and see examples for dorsomedial, dorsolateral, ventromedial and caudal striatum. Although it is not a big part of the paper, the authors should also provide some explanation for the slow EPSC, which could be an artifact due to excessive input. Ideally, they should show that this data is relatively invariant with light intensity.

Please see panel D in Author response image 1 with reviewer 1’s comments for slow EPSC over the light power for all four group (Thal1, Thal2, V1 and vACC). We found no significant correlations between the light power used and the amplitude of the current (R^2^= 0.01, 0.61, -0.01, and 0.07 respectively for Thal1, Thal2, Vis and d/vACC). Although R^2^ for Thal2 is 0.61, the intercept and the slope for the fitting are very small (-0.009 and 0.02 respectively), consistent with the idea that there is no slow current for Thal2 compared toThal1 and d/vACC (Figure 8F).

In addition, we also find no overall difference in light power used among the four groups and no correlation between the PPR and the light power. Please see the response to review 1 #3 for details.

In the current manuscript, we focus on using optogenetic/physiology experiments to demonstrate circuit convergence from 4 subregions of the thalamus and the cortex: Thal1, Thal2, Vis and d/vACC. We feel that these functional confirmations are necessary to complete the story by establishing the functional relevance of the anatomical maps we present. Examining previously under-explored striatal subregions, while extremely interesting and important, would require systematic efforts to target small thalamic sub-regions. Although we can achieve higher-than-injection-volume resolution by utilizing overlapping thalamic injections, it remains challenging to target small thalamic subregions we described with any single injection. Therefore, we feel that it is beyond the scope of current work.

In light of this discussion, we have now re-worded our summary to clearly state that only example pathways were tested for optogenetic experiments. We wrote: ‘With all the pathways tested, the anatomically described corticostriatal and thalamostriatal projections were confirmed to be functional using optogenetic approaches.’

6) The authors should discuss in detail how their work relates to Hintiryan et al., 2016.

See our response to Essential revision #6.

Reviewer #3:

This paper shows how far we have come technically. The future of connectivity studies looks a lot like this paper. It is exciting to see a comprehensive approach to a large and diverse projection like the corticostriatal pathway.

However, the results make a stark contrast with the results from traditional methods of mapping the corticostriatal projection, and I have more confidence in the previous results. The results here indicate much less structure in the corticostriatal projection than expected, and it may be the projection is less structured in mice than in larger animals. Both the old and new methods (and primates and mice) have their strong and weak points. It is great to cover the entire cortex. In the previous generation of this kind of work it was never possible to do more than a fragment of the cortex in any one study. The positive side of the old primate work is that cortical regions were much better defined, and the projections were seen at higher resolution. Not only are functional regions larger in primates, but it was often possible to define regions functionally, rather than only by position on the cortex. This allowed injections to be placed far from functional boundaries, and even in functional subregions (e.g. hand area of somatosensory cortex). A lesson of those old papers was that cortical regions that are close to each other might be functionally disparate and have disparate projections in the striatum. That is, although they projected to the same general area of the striatum (e.g. the somatosensory cortex recipient region), injections in the hand and foot somatosensory cortex did not have convergent projections at the microscopic level, and so did not converge on single striatal neurons. My first concern about this study is that all of those principles learned from the primate work have been jettisoned, and a strictly spatial scheme is used. Because functional areas of the cortex in the mouse are so small, it is likely that most of these injections involve very different functional cortical regions with different projections in the striatum, and are being superimposed to make the projection look less specific than it is. The same could be said about the thalamic projections. Stated differently, if the cortex or thalamus contained an affine map of function, the lost resolution in the injections could be recovered by comparing nearby injections. If it is not, then this will not work. I think the previous work suggests that it is not.

One method that has served well in the past when functional mapping was unavailable was to look at the projections in the thalamus. Often, the precise set of cortical areas involved in a tracer injection can be inferred from its thalamic projections, as these have such sharp boundaries and corticothalamic relations are so well known.

The reviewer has pointed out the exact advantages and the disadvantages of the mice mesoscopic mapping. The advantages of our study in mice are two folds. First, because mouse brains are small, we were able to cover all cortical subregions and > 93% volume of the thalamus with individual injections of small (500-600 µm) sizes, and image the entire projections of every injection. The level of comprehensiveness in documenting thalamic projections has not been previously achieved in any mammalian species. Second, the comprehensiveness of the dataset allow us to perform quantitative analyses that are only possible with full dataset, but not with example images. For example, conventional descriptions of thalamic projections are mostly based on thalamic nucleus demarcations which, when compared to cats or primates, are less distinct and sometimes ambiguous in mice (Jones 2007, pg 52). As better stated in the textbook by Sherman and Guillery (2006), ‘The concept of the thalamic nucleus as a single structural, functional, and connectional entity has barely survived advancing techniques and new information. We stay with the thalamic nuclei as one of our prime analytical tools because, as yet, we have little to use in its place’. In our studies, we take the unique opportunity to establish nucleus-independent thalamic projection maps by using model thalamus based on averaging 70-100 mouse brains (Hunnicutt et al., 2014). We also devoted significant effort to connect with the existing literature: for all the confidence maps presented, we overlay the confidence maps with the nucleus demarcations of both Paxinos atlas and Allen Brain atlas (Figures 4B-D, 5F, 6D-E,7F, Figure 5—figure supplement 1C, Figure 5—figure supplement 2B-C, Figure 5—figure supplement 4C and Figure 6—figure supplement 1B).But because everything is smaller, and often time, lack of anatomical landmarks, accurately assigning the cortical subregions can be might be vague and ambiguous, and not trivial. For example, unlike in primate literature, the definition of some cortical subregions themselves can be controversial in mice (e.g., for M1 and M2, see Mao et al., 2011) and even with the ones that have clear functional definitions, their physical landmarks are lacking. Unfortunately, the boundaries of the thalamic nucleus are also much less distinct compared to the ones in the primate as mentioned above. This is indeed a common challenge in the field for mesoscopic mapping in mice at large scale. For thalamic injections, we took full advantage of the high *n* numbers we could get and used overlapping injections to dramatically increase the resolution (Hunnicutt et al., 2014). This algorithm is the operational expressing of what this reviewer mentioned as ‘the lost resolution…recovered by comparing nearby injections’. For cortical injections, we did the best practice possible by sticking to the existing mouse atlas and by being consistent in selection process (that is why < 10% of AIBS injections was selected into the dataset). Even with great care, the lack of clear landmarks in mouse cortical subregion definition may still be a source of variability. We are also limited to what is available in AIBS database for cortical injections which is already the best effort available with current technology with using over 1600 brains for establishing the model brain to assign the subregions. These cortical injections are mostly not overlapping with one another, so we could not employ similar resolution-improving algorithms we did with thalamic injections.

We do not want to claim that we have solved the issue of dealing with less accurate brain region assignment (compared to primate work). Instead, we feel that our work illustrates the exact issues (e.g., injections not confined within a given thalamic nucleus, and ‘involving potentially different functional areas of the cortex’, as the reviewer pointed out). Many of our analyses were, in fact, inspired by the primate work (e.g., work cited Graybiel and colleagues for cortical inputs and Smith and colleagues for thalamic inputs) because we had to think how to take advantage of our data and combat the disadvantages. In the meantime, mouse has become an essential model in understanding the brain circuits. We reason that providing such anatomical maps, despite possible drawbacks, is important.

Two technical notes are also important regarding why our datasets are different from certain traditional data. First, the high resolution imaging is crucial in our analyses because it allowed us to specify the portions of the striatum where there are a lack of projections, and we took consideration of the negative data (lack of projections) when localizing projection origins in the thalamus (see Methods and Figure 3—figure supplement 1). In contrast, most of traditional studies do not discuss any lack of projections due to lack of full brain images, and very few studies make full projections available. The way we categorized and quantified cortical projections requires comprehensive datasets (Figure 2, Figure 2—figure supplement 1, Figure 2—figure supplement 2, Figure 5—figure supplement 1 and Figure 7—figure supplement 1). With such a comprehensive map, any specific element of the circuits will need to be tested functionally in the future to have a thorough comparison across species. Second, that there are two distinct projection patterns of corticostriatal axons, a localized dense core projection and a diffuse projection that generally spans a wider area than the dense projections (Mailly et al., 2013). Many previous mapping studies preferentially focused on the dense projections, particularly when reporting a summary result of several tracing experiments. In our data, we mapped both the dense and diffuse projections, and this revealed some previously underappreciated convergence patterns, such as the diffuse somatosensory-motor inputs to a portion of the limbic striatum (mid-dark blue, Figure 5, 15 clusters) (Draganski et al., 2008), and the widespread diffuse projections of LO/VO to nearly the entire striatal volume (Figure 2). Our dense projection results are highly consistent with the corticostriatal projection distributions reported in the literature (Gruber and McDonald 2012), and studies that separate the dense and diffuse projections describe similarly widespread diffuse projections (Mailly et al., 2013; Haber et al., 2006).

Finally, we also cannot rule out occasional circuit differences at mesoscopic resolution across species due to parallel evolution, as has been found from time to time even between rat and mice (e.g., Schwarz et al., 2015).

We have added the discussion for above points (Discussion).

One test of whether the results are showing us something really new or are just a blurred version of what we already know would be to find some new principle emerge, or to test an old one. I do not see any clear statement of a new principle. I think in this the authors may have missed an opportunity. One principle proposed by previous authors was that cortical areas that are strongly connected with each other converge in the striatum (Yeterian and Van Hoesen, Brain Res, 139:43-63, 1978). It seems to me that the information required to test this idea may be already available in the authors' data set, just by including corticocortical projections.

We have now added analyses to address the hypothesis proposed by Yeterian and Van Hoesen. We first downloaded corticocortical projection data from Allen Institute of Brain Science mouse atlas website and then analyzed projection density between cortical subregions (Figure 6—figure supplement 2). We found evidence of both types of connection patterns, i) for some cortical subregions, they interconnect with subregions that do not converge in the striatum with, ii) some are highly connected if they converge in the striatum (Figure 6—figure supplement 2B-P and Figure 7—figure supplement 1D-H). The first type seems more prevailing. Interestingly, the highly interconnected ones are FrA, M1/2, S1/2, and AI forming highly recurrent networks with convergent subregions, which may be related to highly coordinated functions of sensori-motor integration.

Along this line, we also further integrated cortiocortical connectivity data into our cortico-thalamo-basal ganglia loop analyses (Figure 6—figure supplement 3 and Figure 7—figure supplement 1).

Declaring there to be a sensorimotor domain in the striatum is misleading. The somatosensory and motor systems are very closely connected and in the past many studies have shown that somatosensory and motor function may even overlap spatially in the cortex (of rodents). However, this does not extend to other sensory modalities. For example, visual cortex or its projections to the striatum do not go to the region called sensory by the authors. Throughout the paper, the word sensory seems specifically associated with the somatosensory cortex. I think the authors should replace the word 'sensory' in all cases in which it appears with 'somatosensory'. One thing we have learned from previous studies is that there is very little overlap between visual and somatosensory cortical projections to the striatum, and very little between visual and primary motor cortex. This is evident in Figure 2, but still, there is no recognition that Aud and Vis are sensory cortices. Their projections do not overlap much with motor cortex at all. What does this mean about the idea that all sensory input is 'sensorimotor'?

We agree with the reviewer. The term meant to be used for somatosensory cortical area and it was misleading. We change ‘sensory’ to ‘somatosensory’ throughout the manuscript.
